# Effect of remote ischemic preconditioning on hemostasis and fibrinolysis in head and neck cancer surgery: A randomized controlled trial

**DOI:** 10.1371/journal.pone.0219496

**Published:** 2019-07-08

**Authors:** Andreas Engel Krag, Birgitte Jul Kiil, Christine Lodberg Hvas, Anne-Mette Hvas

**Affiliations:** 1 Thrombosis and Hemostasis Research Unit, Department of Clinical Biochemistry, Aarhus University Hospital, Aarhus, Denmark; 2 Department of Clinical Medicine, Aarhus University, Aarhus, Denmark; 3 Department of Plastic and Breast Surgery, Aarhus University Hospital, Aarhus, Denmark; 4 Department of Intensive Care Medicine, Aarhus University Hospital, Aarhus, Denmark; Maastricht University Medical Center, NETHERLANDS

## Abstract

**Introduction:**

The aim of this randomized controlled trial was to investigate if remote ischemic preconditioning (RIPC) reduced platelet aggregation and increased fibrinolysis in cancer patients undergoing surgery and thereby reduced the risk of thrombosis.

**Materials and methods:**

Head and neck cancer patients undergoing tumor resection and microsurgical reconstruction were randomized 1:1 to RIPC or sham intervention. RIPC was administered intraoperatively with an inflatable tourniquet by four cycles of 5-min upper extremity occlusion and 5-min reperfusion. The primary endpoint was collagen-induced platelet aggregation measured with Multiplate as area-under-the-curve on the first postoperative day. Secondary endpoints were markers of primary hemostasis, secondary hemostasis, and fibrinolysis. Clinical data on thromboembolic and bleeding complications were prospectively collected at 30-day follow-up. An intention-to-treat analysis was performed.

**Results:**

Sixty patients were randomized to RIPC (n = 30) or sham intervention (n = 30). No patients were lost to follow-up. The relative mean [95% confidence interval] collagen-induced platelet aggregation was 1.26 [1.11;1.40] in the RIPC group and 1.17 [1.07;1.27] in the sham group on the first postoperative day reported as ratios compared with baseline (*P* = 0.30). Median (interquartile range) 50% fibrin clot lysis time was 517 (417–660) sec in the RIPC group and 614 (468–779) sec in the sham group (*P* = 0.25). The postoperative pulmonary embolism rate did not differ between groups (*P* = 1.0).

**Conclusions:**

RIPC did not influence hemostasis and fibrinolysis in head and neck cancer patients undergoing surgery. RIPC did not reduce the rate of thromboembolic complications.

## Introduction

Venous thromboembolism is a life-threatening complication in cancer surgery [1]. Adding to the recommended thromboprophylaxis with low-molecular-weight heparins [2], the perioperative thrombosis risk may be further reduced through novel interventions targeting primary hemostasis or fibrinolysis.

Remote ischemic conditioning is a novel intervention that attenuates ischemia-reperfusion injury in acute myocardial infarction [3]. It is administered by inducing brief periods of extremity ischemia and reperfusion with an inflatable tourniquet. Administration of this intervention before an acute ischemic event is termed remote ischemic preconditioning (RIPC) [4]. RIPC attenuates platelet activation in patients undergoing heart catheterization procedures [5, 6] and long-term remote ischemic conditioning increases fibrinolysis in patients with cardiovascular disease [7] and cerebrovascular disease [8]. Correspondingly, a recent systematic review shows that remote ischemic conditioning may reduce the risk of thrombosis in patients undergoing surgery or cardiac procedures [9]. Regarding cancer surgery, RIPC has been shown to reduce the incidence of postoperative ischemic lesions in patients undergoing brain tumor resection [10]. However, no previous trials have investigated the effects of RIPC on hemostasis in cancer patients undergoing surgery.

Patients with large tumors in the head and neck region undergo tumor resection immediately followed by microsurgical reconstruction [11]. In microsurgical reconstruction, autologous tissue flaps are dissected on their vascular pedicle and transferred to the defect following tumor resection. These tissue flaps are ischemic until blood supply is restored by microvascular anastomoses to recipient vessels at the defect site. For example, the fibula bone is transferred as a vascularized tissue flap to reconstruct an excised mandible segment [12]. A meta-analysis estimates the prevalence of venous thromboembolism to 5% following head and neck cancer surgery, and it reports that administration of pharmacologic thromboprophylaxis is inconsistent in these procedures [13].

The objective of this randomized controlled trial was to investigate the effects of RIPC on hemostasis and fibrinolysis in head and neck cancer patients undergoing tumor resection and microsurgical reconstruction. We also investigated the effects of RIPC on thromboembolic and bleeding complications. We hypothesized that RIPC attenuates platelet aggregation and increases fibrinolysis in the postoperative period.

## Materials and methods

### Trial design

This was a single-center, single-blinded, randomized controlled trial comparing RIPC with sham intervention in head and neck cancer patients undergoing microsurgical reconstruction. The trial took place at the tertiary referral center for microsurgical reconstruction at the Department of Plastic and Breast Surgery, Aarhus University Hospital, Aarhus, Denmark. The Central Denmark Region Committees on Health Research Ethics (journal no. 1-10-72-140-15) and the Danish Data Protection Agency (journal no. 1-16-02-358-15) approved the study. The trial was registered at ClinicalTrials.gov (NCT02548377). Written informed consent was obtained from all patients and the Declaration of Helsinki was followed in all aspects. Data set (S1 File), CONSORT 2010 checklist (S2 File), original Danish study protocol (S3 File), and translated English study protocol (S4 File) are attached as supplementary information.

Inclusion criteria were: 1) patients aged ≥ 18 years, with 2) a histologically verified or clinically suspected malignant tumor in the oral cavity, maxillae, mandible, pharynx, larynx, and/or esophagus, scheduled for 3) tumor resection and immediate microsurgical reconstruction with a single free flap. Exclusion criteria were: 1) arterial and/or venous thromboembolism within the past 3 months and 2) microsurgical reconstruction planned with more than one free flap.

### Interventions

The patients underwent tumor resection which was immediately followed by microsurgical reconstruction with a single free flap under general anesthesia. General anesthesia was induced with propofol and remifentanil intravenously, and after tracheostomy, anesthesia was maintained using sevoflurane gas, unless inhalation anesthesia was contraindicated. Patients were randomized to RIPC or sham intervention administered intraoperatively 35 min before expected transfer of the free flap. RIPC was administered by four cycles of 5-min upper extremity occlusion and 5-min reperfusion with an automated tourniquet inflated to 200 mmHg during occlusion (autoRIC Device, CellAegis Devices Inc., Toronto, Canada). The tourniquet was attached to patients randomized to sham intervention but never inflated.

Blood samples were collected from all patients 1) just before surgery, 2) just before administration of study intervention, 3) 3 h post-intervention, 4) 6 h post-intervention, and 5) on the first postoperative day at 06:30 AM. At each time point, 31 ml blood was collected using the following tubes: serum, lithium heparin, EDTA (all BD Vacutainer from Becton, Dickinson and Company, Franklin Lakes, NJ, USA), sodium citrate 3.2% (BD Vacutainer and Vacuette, Greiner Bio-One International GmbH, Kremsmünster, Austria), and hirudin (Roche Diagnostics, Basel, Switzerland). Blood samples were analyzed immediately or centrifuged for 25 min at 2,960 relative centrifugal force with the platelet-poor plasma subsequently stored at -80° Celsius.

Thromboprophylaxis was routinely administered with subcutaneous low-molecular-weight heparins: dalteparin 2,500 IU or tinzaparin 3,500 IU preoperatively after the first blood sample was collected; dalteparin 2,500 IU or tinzaparin 3,500 IU 6 h postoperatively; and dalteparin 5,000 IU or tinzaparin 4,500 IU daily from the first postoperative day continued for 28 days.

### Laboratory analyses

Platelet aggregation was measured in hirudin-anticoagulated whole blood using Multiplate impedance aggregometry (Roche Diagnostics) as previously described [14]. Multiplate area-under-the curve (AUC, AU x min) was reported for the agonists collagen (COLtest, Roche Diagnostics, final concentration 3.2 μg/ml), adenosine diphosphate (ADP) (ADPtest, Roche Diagnostics, final concentration 6.5 μM) and thrombin receptor activating peptide-6 (TRAP) (TRAPtest, Roche Diagnostics, final concentration 32 μM). Because the manufacturer failed to deliver COLtest when 38 patients had been included, this agonist was substituted with Bio/Data Collagen (Bio/Data Corporation, Horsham, PA, USA, final concentration 61.3 μg/mL) in the remaining 22 patients. This new reagent has previously been tested with the Multiplate Analyzer [15].

Fibrin clot lysis was measured in citrated plasma samples with our in-house assay. Samples were analyzed in duplicate using tissue factor diluted 1:5,000 and tPA at a final concentration of 116 ng/ml as previously described [16]. The following parameters were calculated: 50% lysis time (sec) and AUC (AU x sec). The intra-assay variation was below 15%.

Thrombin generation was measured in citrated plasma samples with the Calibrated Automated Thrombogram assay [17]. Samples were analyzed in duplicate using the standard Platelet-Poor Plasma Reagent (Thrombinoscope BV, Maastricht, The Netherlands) containing tissue factor (final concentration 5 pmol/l) and phospholipids (final concentration 4 μmol/l) and Thrombin Calibrator (Thrombinoscope BV) as previously described [18]. Thrombin generation was measured continuously for 60 min. The two thrombograms from each sample were compared and flat curves were deleted manually. AUC termed the endogenous thrombin potential (ETP, nM x min) was reported.

The following commercial ELISA kits were applied on citrated plasma samples: thrombin-antithrombin complex (TAT) (Enzygnost TAT ELISA, Siemens Healthcare GmbH, Erlangen, Germany), prothrombin fragment 1+2 (F1+2) (Enzygnost F1+2 ELISA, Siemens Healthcare GmbH), tissue-type plasminogen activator (tPA) (TECHNOZYM t-PA EDTA ELISA Kit, Technoclone GmbH, Vienna, Austria), plasminogen activator inhibitor-1 (PAI-1) (TECHNOZYM PAI-1 Antigen ELISA Kit, Technoclone GmbH), and thrombin activatable fibrinolysis inhibitor (TAFI) (IMUCLONE Total TAFI ELISA, Sekisui Diagnostics, LLC, Lexington, MA, USA). Soluble P-selectin (sP-selectin) was measured in EDTA plasma (Human P-Selectin/CD62P Immunoassay, R&D Systems, Inc., Minneapolis, MN, USA). All samples were analyzed in duplicate, and the intra-assay variation was below 10%.

Fibrinogen (Claus’ method), antithrombin (activity), prothrombin time (PT, ratio), activated partial thromboplastin time (aPTT), von Willebrand factor (antigen), plasminogen (activity), and protein C (activity) were measured in citrated plasma on CS-2100i (Sysmex Corporation, Kobe, Japan). Hemoglobin, erythrocyte volume fraction (EVF), platelet count, and leukocytes were measured in EDTA-anticoagulated whole blood on Sysmex XE-5000 (Sysmex Corporation). C-reactive protein (CRP), albumin, and creatinine were measured in lithium-heparin plasma on Cobas 6000 (Roche Diagnostics).

### Outcomes

The primary endpoint was collagen-induced platelet aggregation on the first postoperative day. Secondary laboratory endpoints were ADP- and TRAP-induced platelet aggregation, platelet count, von Willebrand factor, sP-selectin, fibrin clot lysis, fibrin D-dimer, plasminogen, tPA, PAI-1, TAFI, ETP, fibrinogen, F1+2, TAT, PT, aPTT, antithrombin, and protein C during and after surgery until the first postoperative day.

The following clinical endpoints were measured: 1) pulmonary embolism diagnosed with chest CT angiography in symptomatic patients, 2) deep venous thrombosis diagnosed with lower extremity vein ultrasonography in symptomatic patients, 3) myocardial infarction diagnosed by relevant electrocardiographic changes and elevated plasma troponins in symptomatic patients, 4) intraoperative bleeding estimated by the volume in suction canisters minus irrigation fluids plus the weight of blood in gauzes plus estimation of blood volume in surgical drapes and on the floor, 5) postoperative drainage until the first postoperative day at 06:30 AM, 6) blood component transfusions intraoperatively and postoperatively, 7) re-operations for hematomas under general anesthesia, and 8) mortality. These clinical data were prospectively collected from medical chart review at 30-day postoperative follow-up unless otherwise specified. The intraoperative period was defined from induction of general anesthesia until the patient was awake after the primary surgical procedure. The postoperative period was defined from the end of general anesthesia until the 30^th^ postoperative day including any re-operations during that period.

### Randomization

The randomization sequence was generated in Microsoft Excel 2013 (Microsoft Corporation, Redmond, WA, USA) with a 1:1 allocation using varying block sizes of 2, 4, 6, and 8. The allocation cards were packed in opaque, sealed envelopes containing aluminum foil and carbon paper. The envelopes were sequentially numbered according the randomization sequence, and the carbon paper transferred the randomization number to the allocation card, ensuring that the randomization sequence could not be violated. Patients were randomized intraoperatively just before administration of the allocated intervention. A scientist not involved with this trial generated the randomization sequence and numbered the envelopes. Patients were enrolled and randomized by investigators from this trial. Patients were blinded to the study intervention. The investigators who performed postoperative follow-up by medical chart review and the surgical team were not blinded to the intervention. Information on allocation was not available to care providers postoperatively.

### Statistical analyses

The sample size was calculated with collagen-induced platelet aggregation (COLtest, Roche Diagnostics) on the first postoperative day as primary endpoint. The mean value for this endpoint is 815 AU x min in healthy individuals with standard deviation (SD) 130 AU x min [14]. A sample size of 23 patients per group was necessary to detect a reduction of 125 AU x min with α = 5% (two-sided) and 1-β = 90%, and we included 30 patients per group to compensate for missing data. Collagen-induced platelet aggregation data were analyzed as relative changes compared with baseline values because two different collagen reagents were used during the trial.

All analyses were performed as intention-to-treat. Distribution of continuous data was assessed with Q-Q plots grouped after study intervention (S5 File). Skewed data underwent logarithmic transformation to evaluate if normality could be obtained before statistical analyses. Normally distributed variables are presented as mean with SD or 95% confidence interval (CI). These variables were tested with the unpaired t-test when comparing two groups at a single time point. Welch’s approximation was used for variables with unequal variances between groups. Variables that did not follow normal distribution are presented as median with range or interquartile range (IQR). These data were tested with the Wilcoxon rank-sum test if normality was not obtained after logarithmic transformation. The differences between groups over time in laboratory markers were tested using the multivariate repeated measures analysis of variance (ANOVA) testing for parallel mean curves. This ANOVA model was validated by Q-Q plots of the residuals which should follow the normal distribution, and by testing for equal SDs and correlations in the two groups. Due to missing values, fibrin clot lysis and thrombin generation data were tested using the mixed-model multivariate repeated measures ANOVA. Categorical variables grouped after study intervention were tested using Fischer’s exact test and the relative risk with 95% CI was calculated for outcomes. *P* < 0.05 was considered statistically significant. The statistical analyses were performed in Stata/IC 13.1 (StataCorp LP, College Station, TX, USA). Figures were produced in GraphPad Prism 7.0 (La Jolla, CA, USA).

## Results

**Fig 1:**
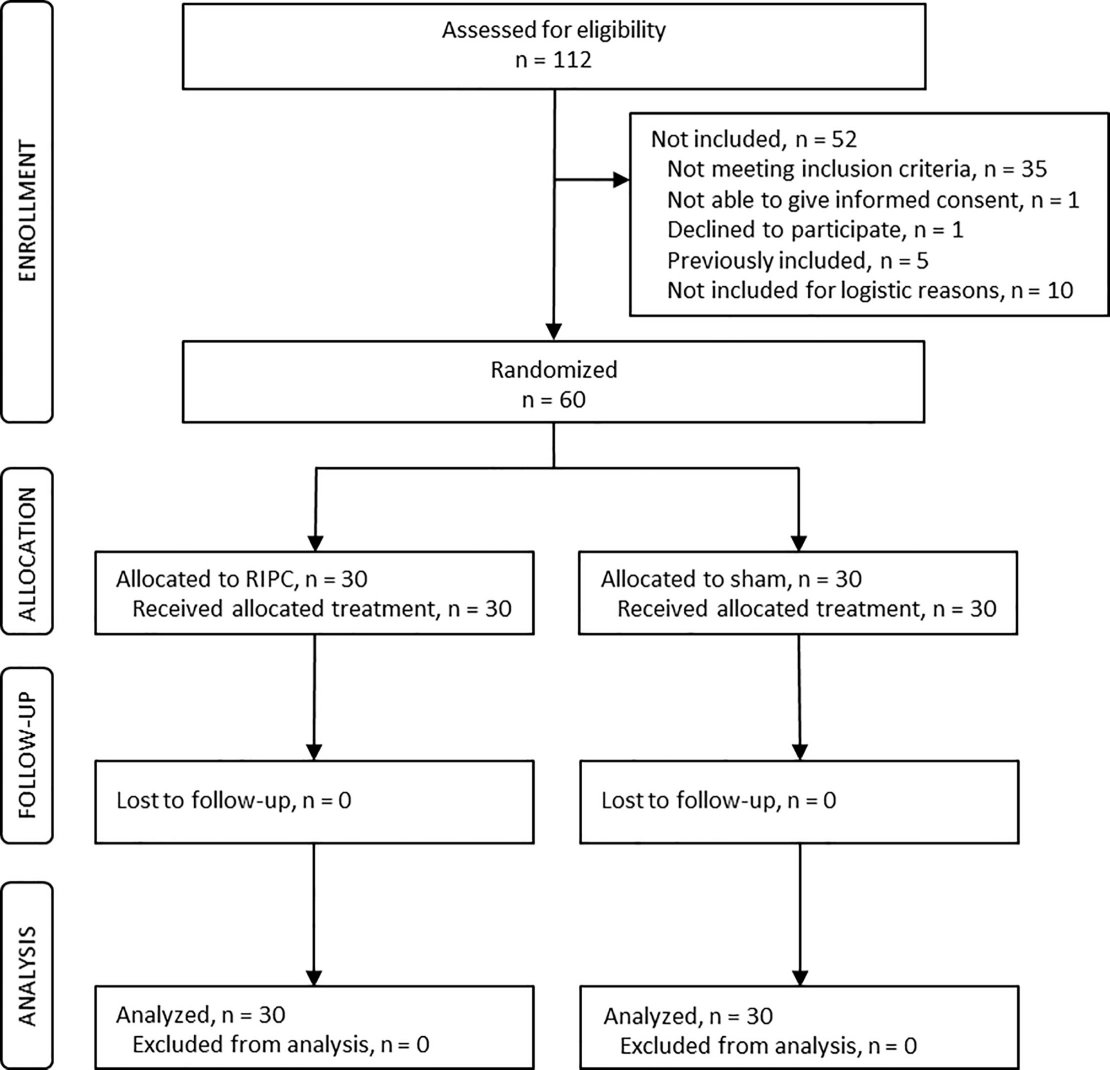
Flow diagram of patient inclusion. Sixty patients were randomized (1:1) to remote ischemic preconditioning (RIPC) or sham intervention. All patients received the allocated intervention and no patients were lost to follow-up. All patients were included in the intention-to-treat analyses.

Fig 1 is a flow diagram featuring patient screening and inclusion. Sixty patients were included in the trial of whom 30 were assigned to RIPC and 30 to sham intervention. All patients received the allocated treatment and were followed for 30 days postoperatively. Patients were recruited from August 2015 to November 2017 and follow-up was completed in December 2017. The following protocol deviations occurred: three patients were converted to reconstruction with two free flaps intraoperatively due to extensive tumor resection (two RIPC, one sham); the pathology report showed no residual tumor in excised tissue in two patients who had undergone preoperative radiotherapy (one RIPC, one sham); and one patient had a benign ameloblastoma (sham). However, all patients were included in the intention-to-treat analyses in the original groups for all endpoints with available data. All blood samples were collected as planned, but assay failure resulted in missing data in fibrin clot lysis and thrombin generation as described in the figure legends. No patients were lost to follow-up. The two groups did not differ in preoperative characteristics, operative characteristics, or anesthesia (all *P* ≥ 0.07) (Table 1).

**Table 1:** Baseline and operative characteristics.

Variable	RIPC (n = 30)	Sham (n = 30)	*P*-value
Sex:			
Male	18 (60%)	19 (63%)	1.0
Female	12 (40%)	11 (37%)	
Age (years)	67 ± 10	64 ± 12	0.22
Body mass index (kg/m^2^)	25 ± 4	23 ± 4	0.18
Smoking status:			
Current	12 (40%)	16 (53%)	0.45
Past	15 (50%)	10 (33%)	
Never	3 (10%)	4 (13%)	
Alcohol consumption (1 unit = 12 g):			
< 21 units per week	21 (70%)	23 (77%)	0.77
≥ 21 units per week	9 (30%)	7 (23%)	
ASA classification:			
1	1 (3%)	0	0.20
2	13 (43%)	19 (63%)	
3	16 (53%)	11 (37%)	
**Co-morbidities**			
Charlson’s co-morbidity score, median(IQR)	5 (4–6)	5 (4–6)	0.98
Diabetes mellitus type 1+2	3 (10%)	3 (10%)	1.0
Arterial hypertension	18 (60%)	14 (47%)	0.44
Atrial fibrillation	5 (17%)	2 (7%)	0.42
**Preoperative biochemistry**			
Platelet count (10^9^/l)	234 ± 65	245 ± 80	0.54
Albumin (g/l)	32 ± 3	32 ± 3	0.66
Creatinine (μmol/l), median(IQR)	68 (60–94)	64 (53–70)	0.07
**Antiplatelet medication**			
Aspirin	4 (13%)	3 (10%)	1.0
Discontinuation (days), median(range)	1 (0–6)	6 (5–6)	0.14
Clopidogrel	2 (7%)	4 (13%)	0.67
Discontinuation (days), median(range)	6 (2–10)	10 (4–30)	0.35
NSAIDs	6 (20%)	7 (23%)	1.0
Discontinuation (days), median(range)	6 (2–10)	10 (4–30)	0.48
**Thromboprophylaxis**			
Preoperative LMWH	29 (97%)	30 (100%)	1.0
Postoperative LMWH	29 (97%)	30 (100%)	1.0
**Operative characteristics**			
Surgery time (min)	398 ± 78	417 ± 95	0.41
General anesthesia time (min)	515 ± 66	518 ± 106	0.90
Anesthetic used for maintenance:			
Sevoflurane	26 (87%)	29 (97%)	0.35
Propofol	4 (13%)	1 (3%)	
**Cancer status**			
Tumor histology:			
Squamous cell carcinoma	25 (83%)	24 (80%)	1.0
Carcinoma, other	3 (10%)	3 (10%)	
Osteosarcoma	1 (3%)	1 (3%)	
Ameloblastoma	0	1 (3%)	
No residual tumor	1 (3%)	1 (3%)	
Secondary malignancy	4 (13%)	3 (10%)	1.0
Neoadjuvant chemotherapy	0	2 (7%)	0.49

Categorical variables are shown as number of patients and frequencies. Continuous variables are shown as mean ± SD except when indicated otherwise. P-values represent unpaired t-test or Wilcoxon rank sum test for continuous variables and Fischer’s exact test for categorical variables.

Abbreviations: ASA: American Society of Anesthesiologists, IQR: interquartile range, LMWH: low-molecular-weight heparin, RIPC: remote ischemic preconditioning.

**Fig 2:**
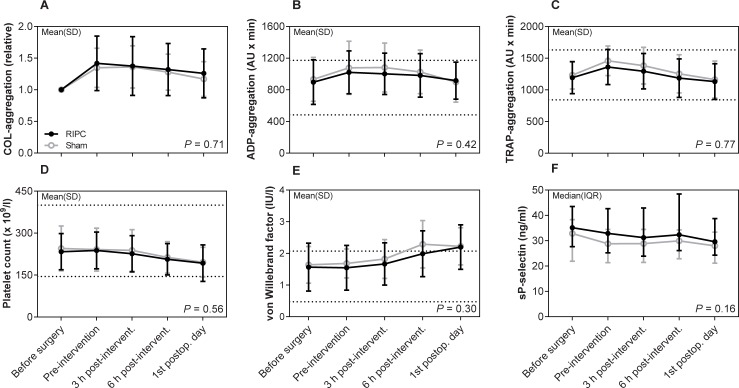
Platelet aggregation and primary hemostasis biomarkers. Data are shown as mean ± SD or median with IQR. Collagen-induced platelet aggregation data are reported as relative changes compared with baseline values because two different collagen reagents were used during the trial. Lines indicate common reference intervals for healthy men and women from our laboratory if available. *P*-values represent the multivariate repeated measures ANOVA. Abbreviations: COL: collagen, ADP: adenosine diphosphate, RIPC: remote ischemic preconditioning, sP-selectin: soluble P-selectin, TRAP: thrombin receptor activating peptide-6.

**Fig 3:**
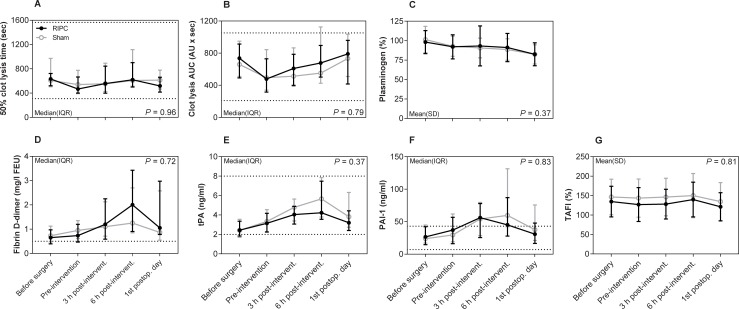
Fibrin clot lysis assay and fibrinolysis biomarkers. Data are shown as mean ± SD or median with IQR. Lines indicate common reference intervals for healthy men and women from our laboratory if available. Reference intervals for tPA and PAI-1 were provided by the manufacturer. *P*-values represent the multivariate repeated measures ANOVA. Mixed-model was used for fibrin clot lysis data. Missing data: Fibrin clot lysis produced flat curves in 18 samples obtained pre-intervention (8 RIPC, 10 sham), 5 samples obtained 3 h post-intervention (3 RIPC, 2 sham), and 14 samples obtained on the first postoperative day (7 RIPC, 7 sham). Fibrin clot lysis curves did not lyse in 4 samples obtained 3 h post-intervention (2 RIPC, 2 sham) and 6 samples obtained 6 h post-intervention (3 RIPC, 3 sham). Abbreviations: PAI-1: plasminogen activator inhibitor-1, RIPC: remote ischemic preconditioning, TAFI: thrombin activatable fibrinolysis inhibitor, tPA: tissue-type plasminogen activator.

**Fig 4:**
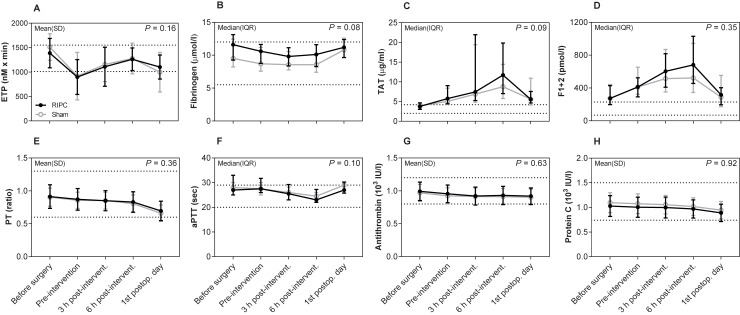
Thrombin generation, secondary hemostasis, and natural anticoagulants. Data are shown as mean ± SD or median with IQR. Lines indicate common reference intervals for healthy men and women from our laboratory if available. Reference intervals for TAT and F1+2 were provided by the manufacturer. *P*-values represent the multivariate repeated measures ANOVA. Mixed-model was used for ETP. Missing data: Thrombin generation produced flat curves in 4 samples obtained pre-intervention (3 RIPC, 1 sham) and in 4 samples obtained on the first postoperative day (3 RIPC, 1 sham). Abbreviations: aPTT: activated partial thromboplastin time, ETP: endogenous thrombin potential, F1+2: prothrombin fragment 1+2, PT: prothrombin time, RIPC: remote ischemic preconditioning, TAT: thrombin-antithrombin complex.

Regarding the primary endpoint, collagen-induced platelet aggregation was not significantly different between the RIPC and sham group on the first postoperative day as shown in the mean AUC ratios with 95% CI 1.26 [1.11;1.40] vs. 1.17 [1.07;1.27] (*P* = 0.30) (Fig 2A). Nor did collagen-induced platelet aggregation differ between the two groups over time (*P* = 0.71). Regarding fibrinolysis, 50% fibrin clot lysis time was shortened in the RIPC group on the first postoperative day with the median (IQR) values 517 (417–660) sec compared with 614 (468–779) sec in the sham group, but statistical significance was not reached (*P* = 0.25) (Fig 3A). Furthermore, 50% lysis time did not differ between the two groups over time (*P* = 0.96). Finally, the remaining markers of primary hemostasis (Fig 2B–2F), fibrinolysis (Fig 3B–3G), and secondary hemostasis (Fig 4A–4H) did not differ between groups over time (all *P* ≥ 0.08). There was no significant difference between the two groups over time in hemoglobin, EVF, leukocytes, and CRP (Table 2).

**Table 2:** Hematology and immunology.

Biomarker	Reference interval	Group	Before surgery	Pre-intervention	3 h post-intervention	6 h post-intervention	1st postop. day	*P*-value
Hemoglobin (mmol/l) (Mean±SD)	7.3–10.5	RIPC	7.7 ± 1.0	7.3 ± 1.1	7.0 ± 1.1	6.9 ± 1.0	6.3 ± 0.9	0.20
		Sham	7.6 ± 0.9	7.1 ± 0.9	6.9 ± 0.9	6.7 ± 0.9	6.2 ± 0.8	
EVF (Mean±SD)	0.35–0.50	RIPC	0.37 ± 0.05	0.36 ± 0.05	0.34 ± 0.05	0.33 ± 0.05	0.30 ± 0.05	0.17
		Sham	0.37 ± 0.04	0.35 ± 0.04	0.33 ± 0.04	0.32 ± 0.04	0.30 ± 0.04	
Leukocytes (x 10^9^ / l) (Mean±SD)	3.5–10.0	RIPC	7.4 ± 2.4	10.3 ± 3.6	13.4 ± 4.4	14.7 ± 4.7	11.9 ± 3.9	0.63
		Sham	7.8 ± 2.5	10.6 ± 4.0	14.5 ± 3.5	15.2 ± 3.9	12.3 ± 3.6	
CRP (mg/l) Median(IQR)	< 8.0	RIPC	6.4 (2.7–10.9)	5.5 (2.8–10.6)	6.1 (3.4–11.5)	12.2 (8.7–22.2)	79.3 (47.1–101.9)	0.20
		Sham	3.8 (2.0–7.3)	3.9 (1.7–7.2)	4.6 (2.8–12.5)	10.0 (6.1–16.7)	77.7 (59.4–91.1)	

The reference intervals are common for healthy men and women from our laboratory. P-values represent the multivariate repeated measures ANOVA testing the difference between groups over time.

Abbreviations: CRP: C-reactive protein, EVF: erythrocyte volume fraction, IQR: interquartile range, RIPC: remote ischemic preconditioning, SD: standard deviation.

Clinical outcomes data are presented in Table 3. The overall prevalence of symptomatic venous thromboembolism was 8%. All events were pulmonary embolism and there was no difference between the RIPC group (*n* = 2) and sham group (*n* = 3), relative risk 0.67 with 95% CI [0.12;3.71] (*P* = 1.0). The two groups were similar regarding estimated intraoperative bleeding (*P* = 0.64) and postoperative drainage (*P* = 0.66). We observed that fewer patients received postoperative red blood cell transfusion in the RIPC group (*n* = 9) than in the sham group (*n* = 14) with a statistically non-significant relative risk of 0.64 with 95% CI [0.33;1.25] (*P* = 0.29). Also, fewer patients underwent re-operation for hematoma in the RIPC group (*n* = 3) than in the sham group (*n* = 6) with a statistically non-significant relative risk of 0.50 with 95% CI [0.14;1.82] (*P* = 0.47). One patient in the RIPC group died before hospital discharge after having been diagnosed with pulmonary embolism. Finally, no adverse effects related to study interventions were observed.

**Table 3:** Clinical outcomes at 30 days postoperatively.

	RIPC (n = 30)	Sham (n = 30)	Relative risk (95% CI)	*P*-value
**Thromboembolic complications**				
Pulmonary embolism	2 (7%)	3 (10%)	0.67 [0.12;3.71]	1.0
Myocardial infarction	0	1 (3%)		1.0
**Intraoperative bleeding**					
Estimated intraoperative bleeding (ml)	418 [276;560]	459 [353;564]		0.64
Intraoperative red blood cell transfusion	1 (3%)	2 (7%)	0.50 [0.05;5.22]	1.0
Intraoperative platelet transfusion	1 (3%)	0		1.0
**Postoperative bleeding**				
Drainage (ml), median(IQR)	50 (20–100)	60 (25–120)		0.66
Postoperative red blood cell transfusion	9 (30%)	14 (47%)	0.64 [0.33;1.25]	0.29
Postoperative platelet transfusion	0	1 (3%)		1.0
Re-operation for hematoma	3 (10%)	6 (20%)	0.50 [0.14;1.82]	0.47
**Mortality**	1 (3%)	0		1.0

Categorical variables are shown as number of patients with frequencies and relative risk with 95% CI and *P*-values from Fischer’s exact test. Continuous variables are shown as mean with 95% CI or median with IQR and *P*-values from unpaired t-test. Postoperative drainage was measured until the first postoperative day at 06:30 AM.

Abbreviations: CI: confidence interval, IQR: interquartile range, n.a.: not available, RIPC: remote ischemic preconditioning.

## Discussion

In the present randomized controlled trial, we investigated the effects of RIPC on hemostasis and fibrinolysis in head and neck cancer patients undergoing microsurgical reconstruction. RIPC failed to reduce platelet aggregation and did not significantly increase fibrinolysis during or after surgery. Furthermore, RIPC did not influence secondary hemostasis and it did not reduce postoperative thromboembolic complications.

This is the first study investigating the effects of RIPC on platelet aggregation in cancer patients undergoing surgery. Platelet aggregation on the first postoperative day was primary endpoint to evaluate the effect of the study intervention with minimal interference from surgery-induced hemostatic activation. Previous studies showed that RIPC reduced platelet activation, as monocyte-platelet aggregates were reduced measured with flow cytometry in healthy men [19] and in randomized trials of cardiovascular disease patients undergoing exercise stress test [20] and minimally invasive heart procedures [5, 6]. However, remote ischemic conditioning did not affect platelet aggregation, when measured with Multiplate impedance aggregometry in healthy men with no ischemic event [21]. Contrary to these studies on cardiovascular disease patients, the present study included head and neck cancer patients with various co-morbidities undergoing a major surgical intervention under general anesthesia. While previous studies measured platelet activation with flow cytometry, we measured platelet aggregation with Multiplate impedance aggregometry which is more affected by platelet count and has a higher coefficient of variation [22–24].

The influence of RIPC on fibrinolysis in patients undergoing surgery has not previously been studied. In the present study, RIPC did not affect fibrinolysis which was measured using the fibrin clot lysis assay and the biomarkers fibrin d-dimer, plasminogen, tPA, PAI-1, and TAFI. In a previous randomized controlled trial, bilateral remote ischemic conditioning twice daily increased tPA and reduced PAI-1 after 15 days of treatment in elderly Chinese patients with a previous ischemic stroke [8]. Further, remote ischemic conditioning once daily shortened fibrin clot lysis time in healthy individuals and increased tPA in patients with chronic ischemic heart failure after 28 days of treatment in another study [7]. Contrary to this, two studies of healthy men reported that a single administration of remote ischemic conditioning did not influence fibrinolysis [25, 26]. Hence, long-term administration of remote ischemic conditioning may be necessary to increase fibrinolysis based on these previous studies and the present study.

Finally, RIPC did not influence thrombin generation or secondary hemostasis markers in the present study. In a porcine model, we have shown that remote ischemic conditioning increased ETP but also aPTT during the reperfusion phase of tissue flap ischemia-reperfusion injury [27]. In human studies, remote ischemic conditioning did not influence thrombin generation in healthy men with no ischemic event [26]. But RIPC increased the expression of fibrinogen preproprotein in children undergoing congenital heart surgery [28], and administration of remote ischemic conditioning up to four times on non-consecutive days prolonged PT but not aPTT in patients who had undergone surgical clipping or coiling for subarachnoid hemorrhage [29]. Hence, remote ischemic conditioning does not seem to influence secondary hemostasis in a clear hypo- or hypercoagulable direction in humans.

A previous prospective study on head and neck cancer surgery reported pulmonary embolism in 1%, deep venous thrombosis in 7%, and asymptomatic superficial lower extremity vein thrombosis in 5% of cases without routine use of pharmacologic thromboprophylaxis. That study employed postoperative screening for thrombosis by lower extremity vein ultrasonography [30]. We found a substantially higher rate of pulmonary embolism occurring in 8% of the total population despite of pre- and postoperative administration of low-molecular-weight heparins. This variation may be explained by different diagnostic strategies, as we performed chest CT angiography only in patients with postoperative respiratory symptoms. Thus, RIPC did not reduce postoperative thromboembolic complications in the present study.

In the present study, we observed a trend towards fewer patients receiving postoperative red blood cell transfusion and fewer patients undergoing re-operations for hematoma in the RIPC group. A previous randomized controlled trial on heart valve replacement surgery reported that remote ischemic perconditioning significantly reduced postoperative drain output, but it did not reduce postoperative red blood cell transfusions [31]. Thus, RIPC did not reduce postoperative thromboembolic complications in the present study. However, further studies are needed to prove if RIPC induces protection against operative bleeding.

The major strength of the present study is its randomized controlled trial design. Biochemical and clinical data were prospectively collected, and no patients were lost to follow-up. The present study is a comprehensive analysis of the acute effects of RIPC on hemostasis and fibrinolysis in head and neck cancer patients undergoing surgery. Several of the biomarkers used in our study can predict venous thromboembolism in cancer patients [32–35]. Experimental studies have shown that tissue-protection induced by remote ischemic conditioning occurs in an early phase lasting 4–5 h after the intervention [36, 37] and a late phase starting 24–72 h after the intervention [38]. Hence, we administered RIPC intraoperatively aiming at placing the acute phase of potential protection from RIPC in the critical early postoperative period.

The following limitations should be considered. General anesthesia was induced with propofol which was continued until temporary tracheostomy was established in the present study. Subsequently, general anesthesia was maintained with sevoflurane, unless contraindicated. However, propofol has been shown to diminish cardio protection from RIPC, measured with postoperative serum troponin I release, when administered during cardiac surgery [39]. Contrary to this, RIPC by intermittent cross-clamping of the common iliac artery, significantly reduced postoperative serum troponin I release and the risk of postoperative myocardial infarction in elective abdominal aortic aneurysm repair patients, who were anesthetized with propofol induction and desflurane gas for maintenance [40]. Hence, in that study propofol induction did not influence cardio protection mediated by RIPC. It is unknown how anesthetics affect RIPC-mediated modulation of hemostasis, and the pathway may differ from RIPC-mediated cardio protection and thereby not be influenced by propofol. Also, the cessation of collagen delivery from the manufacturer compromised the study as conversion to relative collagen-induced platelet aggregation AUC values was necessary due to substantial differences in absolute AUC values between the two different collagen reagents. In addition, we have missing fibrin clot lysis and thrombin generation values due to assay failure. Hence, we analyzed these data using the mixed-model univariate repeated measures ANOVA which is not influenced by missing data. As only symptomatic patients underwent diagnostic imaging for venous thromboembolism there is a risk of outcome bias for this endpoint in our trial. Furthermore, it was not possible to blind investigators and surgeons for the intervention, but patients and care providers were blinded. Animal studies have shown that local ischemic preconditioning prolonged bleeding time measured 72 h after the intervention [41, 42]. Hence, RIPC may modulate hemostasis in a late phase response occurring more than 24 h after the intervention which could not be shown in the present study. However, we decided only to collect blood samples during the first 24 h of surgery where patients were admitted to the surgical observation unit and received standardized care.

This is the first randomized controlled trial investigating the effects of RIPC on hemostasis and fibrinolysis in cancer surgery. As we observed a trend towards fewer postoperative bleeding complications in RIPC treated patients, future studies should investigate the potential hemostatic effect of remote ischemic conditioning in, e.g., surgical patients or patients with intracerebral hemorrhage. Because remote ischemic conditioning is safe, non-invasive, and low-cost, even small improvements in outcome should be considered important.

In conclusion, RIPC did not influence hemostasis and fibrinolysis in head and neck cancer patients undergoing surgery and it did not reduce the risk of postoperative thromboembolism. RIPC may reduce postoperative bleeding complications in surgery.

## Supporting information

S1 File Study data set. (XLSX)

S2 File CONSORT 2010 checklist. (DOC)

S3 File Original Danish study protocol. (PDF)

S4 File Translated English study protocol. (PDF)

S5 File Q-Q plots. (PDF)
