# Supplementary material for: Effect of remote ischemic preconditioning on hemostasis and fibrinolysis in head and neck cancer surgery: A randomized controlled trial
Source: PLoS One. 2019 Jul 8;14(7):e0219496. doi: 10.1371/journal.pone.0219496 (PMC6613699; doi:10.1371/journal.pone.0219496)
Supplement: S3 File — (PDF) [file pone.0219496.s003.pdf]

Behandling med blodtryksmanchet ved hoved-halsrekonstruktion

Overordnet formål

Det overordnede formål er at undersøge, om en ny behandling kaldet 'remote iskæmisk prækonditionering' øger sandsynligheden for succesfuld mikrokirurgisk rekonstruktion.

Der udføres et randomiseret klinisk forsøg: *"Behandling med blodtryksmanchet ved hoved-halsrekonstruktion"*. I forsøget undersøges effekten af remote iskæmisk prækonditionering, hos patienter som undergår resektion af malign tumor i hoved-halsområdet og rekonstruktion med fri lap.

Litteratursøgning

Der er foretaget litteratursøgning i PubMed/MEDLINE med følgende søgninger:

Søgning (7. april 2015)	*Antal fundne referencer*
"Ischemic Preconditioning"[Mesh] AND "Hemostasis"[Mesh]	20
"Blood Coagulation"[Mesh] AND "Ischemic Preconditioning"[Mesh]	4
"Ischemic Preconditioning"[Mesh] AND "Fibrinolysis"[Mesh]	2
"Thrombosis"[Mesh] AND "Ischemic Preconditioning"[Mesh]	20
ischemic preconditioning coagulation	35
"Surgical Flaps"[Mesh] AND "Ischemic Preconditioning"[Mesh]	78
"Surgical Flaps"[Mesh] AND "Ischemic Preconditioning"[Mesh] AND "Hemostasis"[Mesh]	0
"Surgical Flaps"[Mesh] AND "Ischemic Preconditioning"[Mesh] AND "Thrombosis"[Mesh]	0

Desuden er relevant litteratur identificeret ved gennemgang af litteraturlisten i de fundne referencer.

Baggrund

Ved mikrokirurgisk rekonstruktion anvendes patientens eget væv til rekonstruktion af defekter efter bl.a. tumorfjernelse eller traumer. Vævsblokke høstes fra et donorsted og transplanteres til defekten, hvor blodforsyningen genoprettes ved sammensyning af små kar under operationsmikroskopet. Disse vævsblokke betegnes frie lapper. Eksempelvis anvendes fibula med hud fra underbenet til rekonstruktion af kæbeknogle og mundslimhinde. Trombose i karanastomoserne eller lappens mikrokredsløb er den primære årsag til total lapnekrose, som finder sted i 1-9 % af tilfældene og kan være fatalt for patienten. [1-3] Derfor er der brug for udvikling af nye behandlingsmetoder til at reducere tromboserisikoen og dermed risikoen for laptab.

Ved mikrokirurgisk rekonstruktion opstår der pga. det operative indgreb en hyperkoagulabel tilstand, endotheldysfunktion og ændring af blodgennemstrømningen. Det er velbeskrevet, at onkologiske patienter har hyperkoagulabelt blod og dermed øget tromboserisiko. [4] Endotheldysfunktion opstår i lappens mikrokredsløb efter iskæmi og i anastomoserne pga. overklipning af karvæggen og suturering. I patientens kredsløb fører stase og hypoxi til skade på endothelet. Disse ændringer i koagulationssystemet medfører øget tromboserisiko og har afgørende betydning for udfaldet af rekonstruktion med fri lap.

Iskæmisk prækonditionering, hvor korte episoder af iskæmi og reperfusion induceres før langvarig iskæmi, beskytter væv og organer mod akut iskæmi-reperfusionsskade. [5]

Prækonditionering kan enten foretages direkte på organet før iskæmi, eller ved afklemning af væv anatomisk adskilt fra det iskæmiske væv – såkaldt *remote iskæmisk prækonditionering*. [6] Desuden findes hhv. *remote iskæmisk perkonditionering*, som udføres efter at akut iskæmi er opstået, men før reperfusion, og *postkonditionering* som udføres ved reperfusion af det iskæmiske organ. [7, 8] Randomiserede kliniske forsøg med akut myokardie infarkt- og apopleksipatienter har vist gavnlig effekt af remote iskæmisk perkonditionering udført ved afklemning af overekstremiteten med en oppustelig manchet. [9, 10]

Mekanismen bag effekten af remote iskæmisk prækonditionering er ukendt, men undersøgelser har fokuseret på frisætning af humorale faktorer eller et neurogent respons. [11-13] Desuden er der beskrevet systemiske effekter, som omfatter koagulationssystemet og immunsystemet. [14-16] Iskæmisk prækonditionering og remote iskæmisk prækonditionering af lapper er tidligere beskrevet i eksperimentelle studier. [13, 17-20] Der er hidtil kun publiceret én klinisk serie med iskæmisk prækonditionering af seks stilkede lapper til brystrekonstruktion, og to humane studier som viste, at remote iskæmisk konditionering forbedrer blodforsyningen i det kutane kredsløb hos raske forsøgspersoner. [21-23] Der er således ikke publiceret kliniske studier med højt evidensniveau om den mulige gavnlige effekt af remote iskæmisk prækonditionering ved rekonstruktionskirurgi.

Forskningsgruppen
Forsøget gennemføres på Plastikkirurgisk Afdeling Z og Center for Hæmofili og Trombose, Klinisk Biokemisk Afdeling ved Aarhus Universitetshospital (AUH). Forsøget er del af et ph.d.-projekt ved Health, Aarhus Universitet. Forskningsgruppen består af:

Forsøgsansvarlig og sponsor: Anne-Mette Hvas, Professor, Overlæge, ph.d., Center for Hæmofili og Trombose, Klinisk Biokemisk Afdeling, AUH.
Ph.d.-studerende: Andreas Engel Krag, stud.med., Center for Hæmofili og Trombose, Klinisk Biokemisk Afdeling, AUH.
Christine Lodberg Hvas, Læge, ph.d., Anæstesiologisk Afdeling, AUH.
Birgitte Jul Kiil, Overlæge, Plastikkirurgisk Afdeling Z, AUH.
Gete Toft Eschen, Overlæge, Plastikkirurgisk Afdeling Z, AUH.

Specifikke formål
- At undersøge om remote iskæmisk prækonditionering nedsætter trombocyt-aggregationen og dermed tendens til mikrotrombosering ved mikrokirurgisk hoved-halsrekonstruktion.
- At undersøge om remote iskæmisk prækonditionering fremkalder fibrinolyse under operation hos patienter med hoved-halscancer.
- At undersøge om remote iskæmisk prækonditionering forbedrer blodforsyningen i frie lapper.

Specifikke hypoteser
- Remote iskæmisk prækonditionering nedsætter trombocytaggregationen.
- Remote iskæmisk prækonditionering øger fibrinolysen.
- Remote iskæmisk prækonditionering forbedrer blodforsyningen i den frie lap.

Forsøgspersoner, metoder og materialer
Design: Randomiseret, kontrolleret, enkeltblindet forsøg.

Forsøgspersonen randomiseres på operationsstuen, efter at de er lagt i general anæstesi til enten: Remote iskæmisk prækonditionering eller ingen remote iskæmisk prækonditionering (kontrolgruppe). Behandlingen er blindet for forsøgspersonen.
Remote iskæmisk prækonditionering: Udføres ved hjælp af en blodtryksmanchet påsat forsøgspersonens højre eller venstre overarm. Der gives fire cykler med fem minutters iskæmi ved oppustning af blodtryksmanchetten til et tryk på 200 mmHg. Hver periode med iskæmi adskilles af en periode på fem minutters reperfusion.
Hvis forsøgspersonen randomiseres til kontrolgruppen påsættes blodtryksmanchetten, men den oppustes ikke.

Inklusionskriterier:

- Alder over 18 år.
- I stand til at give informeret samtykke.
- Skal undergå resektion af malign tumor og rekonstruktion med én fri lap i hoved-halsområdet på Plastikkirurgisk Afdeling Z, AUH.

Eksklusionskriterier: Venøs eller arteriel tromboemboli indenfor de seneste tre måneder.

Datakilder
Laboratorieanalyser:

- *Primær hæmostase:* Trombocyttal og trombocytaggregation målt ved Multiplate®.
- *Sekundær hæmostase:* APTT, INR, antitrombin og trombin-antitrombinkompleks.
- *Fibrinolyse:* Clotlyse assay, protein C, solubelt trombomodulin, fibrin d-dimer, protrombin fragment 1+2, plasminogen aktivator inhibitor-1, tissue plasminogen aktivator-antigen, plasminogen og trombin aktiveret fibrinolyse inhibitor.
- *Globalt mål for hæmostase:* Trombingeneration.
- *Systemisk inflammation:* CRP, leukocytter, fibrinogen, interleukin-6, von Willebrand faktor (antigen), tissue faktor, komplementproteiner og DNA-histonkomplekser.

På operationsdagen udtages i alt fire blodprøver under/efter operationen via en arteriekanyle, som anlægges i forbindelse med bedøvelsen.
Den første postoperative dag tages yderligere én blodprøve via arteriekanyle eller ved venepunktur i forsøgspersonens arm. Ved hver blodprøvetagning udtages 30,8 mL blod.
Dermed udtages i alt 123,2 mL blod under operationen og 30,8 mL blod den første postoperative dag. I alt udtages således 154 mL blod i hele forsøget.

Lappens blodforsyning:
Ved anvendelse af metoden dynamisk termografi[24] beskrives lappens blodforsyning under operationen ved måling før og efter remote iskæmisk prækonditionering/sham. Metoden består i kortvarig lokal nedkøling af vævet, hvorefter vævet filmes med et infrarødt kamera under genopvarmning til normal perifer kropstemperatur.

Kliniske oplysninger:

- Tidligere behandling for hoved-halscancer
- Co-morbiditeter
- Medicin
- Forløbet af den aktuelle operation
- Re-operationer
- Morbiditet og mortalitet efter akutelle operation

Follow-up for indsamling af kliniske data er 90 dage (+/- 14 dage). Data indsamles ved videregivelse af oplysninger fra patientjournalen og Fælles Medicinkort samt ved ambulant kontrol i Plastikkirurgisk Ambulatorium, AUH.

Indhentelse af informeret samtykke

Den første kontakt foregår i DHOZ-ambulatorium, AUH, hvor nyhenviste patienter med hoved-halscancer vurderes af øre-næse-halskirurg, plastikkirurg, kæbekirurg og onkolog med henblik på operation. Patienter som indstilles til operation, og som opfylder inklusionskriterierne, vil modtage mundtlig og skriftlig information om forsøget ved overlæge Birgitte Jul Kiil, overlæge Gete Toft Eschen, overlæge Jens Bengaard, 1. reservelæge Hans Henrik Møller Nielsen (Plastikkirurgisk Afdeling Z, AUH) eller ph.d.-studerende Andreas Engel Krag.
Samtalen vil foregå uforstyrret i ambulatoriekonsultationen, hvor patienten også kan have en bisidder med. Forsøgspersonen kan enten afgive informeret samtykke med det samme, eller få betænkningstid indtil indlæggelsesdagen på Plastikkirurgisk Afdeling Z, AUH, som normalt finder sted fem dage før operationen. Mellem patientens besøg i DHOZ-ambulatoriet og operation vil der som udgangspunkt maksimalt gå fjorten dage.

Biobanker

<u>Forskningsbiobank</u>:
Ved hver blodprøvetagning analyseres ca. 14 mL blod med det samme, mens resten nedfryses som plasma med henblik på senere analyse i større partier. Forskningsbiobanken oprettes på Klinisk Biokemisk Afdeling, AUH. Disse analyser udføres inden projektperiodens udløb d. 31.1.2020. Samtykke til forskningsbiobanken er en forudsætning for at deltage i forsøget. Ved forsøgets afslutning vil evt. overskydende materiale blive anonymiseret og overført til en biobank til fremtidig forskning.

<u>Biobank til fremtidig forskning</u>:
Forsøgspersonerne spørges separat om der må udtages blod til en biobank til fremtidig forskning.

Styrkeberegning og statistiske analyser

I følgende styrkeberegning anvendes α på 0,05 (to-sidet) en styrke (1- β) på 90 %. Hovedeffektmål er ændring i trombocytaggregationen målt ved Multiplate®. Fra et tidligere studie er middelværdi for areal under kurven ved collageninduceret trombocyt-aggregation fundet til 815 (AU*min) med standarddeviation på 130 hos raske forsøgspersoner. [25] Den mindste relevante ændring er 125, hvilket fører til 23 forsøgspersoner i hver gruppe. For at tage højde for missing data og stor spredning på trombocytaggregation hos cancerpatienter inkluderes ~~25~~ 30 forsøgspersoner i hver gruppe.

Ændringen i biokemiske markører i grupperne over tid testes med ANOVA for parametriske data eller Friedmans test for nonparametriske data. Forskellen mellem de to grupper sammenlignes til hvert prøvetagningstidspunkt med en uparret t-test for parametriske data eller med Wilcoxon rank-sum test for nonparametriske data.

Etiske overvejelser, risici, bivirkninger og ulemper

Forsøget søges godkendt hos De Videnskabsetiske Komitéer for Region Midtjylland og anmeldes til Region Midtjyllands fælles paraplyanmeldelse til Datatilsynet. Forsøget vil blive gennemført i overensstemmelse med Helsinki-deklarationen.

Der er ikke tidligere beskrevet risici eller bivirkninger ved remote iskæmisk prækonditionering. De fire blodprøver som udtages under operationen, vil blive taget via en arteriekanyle, som alligevel skal anlægges af anæstesien til invasiv blodtryksmonitorering. Ved blodprøven som skal tages den første postoperative dag, vil nogle af forsøgspersonerne stadig have deres arteriekanyle, hvorfra blodprøven kan tages. Hos forsøgspersoner hvor arteriekanylen er seponeret, vil blodprøven blive taget ved venepunktur i en arm. Ved venepunkturen kan der forekomme et hæmatom, og i meget sjældne tilfælde infektion.
Ved deltagelse i forsøget udtages i alt 154 mL blod fra forsøgspersonen. Forskningsgruppen vurderer ikke, at dette blodtab udgør en risiko for forsøgspersonerne.
Dynamisk termografi er en noninvasiv metode, som er uden risiko for patienten.

Hvis der mod forventning opstår uventede bivirkninger under forsøget, vil de blive indrapporteret til Videnskabsetisk Komite.

Den enkelte forsøgsperson kan ikke forventes at have gavn af at deltage i forsøget. Det er forskningsgruppens opfattelse, at den nye viden som forsøgets resultater vil bidrage med, opvejer forsøgspersonernes ulemper og risici forbundet med deltagelse i forsøget.

Omstændigheder hvor forsøget afbrydes
Den enkelte forsøgsperson udgår af forsøget i tilfælde af tilbagetrækning af samtykkeerklæring. Forskningsgruppen vurderer ikke, at der er omstændigheder hvorunder forsøgene som helhed kan blive afbrudt.

Håndtering og arkivering af data
Ved håndtering, bearbejdning og arkivering af indsamlede data følges Persondataloven og Datatilsynets retningslinjer. Alle personlige data anonymiseres ved projektets afslutning. Indsamlede data opbevares 15 år efter projektets afslutning på Klinisk Biokemisk Afdeling, AUH.

Videregivelse af patientoplysninger til den forsøgsansvarlige
Efter opnåelse af samtykke videregives oplysninger fra forsøgspersonens patientjournal og Fælles Medicinkort med henblik på indsamling af kliniske data, som er beskrevet under datakilder i afsnittet 'Forsøgspersoner, metoder og materialer'.

Økonomi
Forskerne er selv initiativtagere til projektet. Aarhus Universitet har bevilget et fuldt MD/PhD-stipendium til Andreas Engel Krag. Kræftens Bekæmpelse har doneret 840.000 kr. til driftsmidler og bioanalytikerløn i 2016-18. Bevillinger til betaling af øvrige driftsomkostninger søges hos private fonde og Aarhus Universitetshospital. Der udbetales ikke vederlag til forsøgspersonerne.

Perspektiver
Totalt laptab finder sted i op til 10 % af alle rekonstruktioner, og forløbet kan være fatalt ved hoved-halsrekonstruktion. Forsøget vil vise, om remote iskæmisk prækonditionering nedsætter risikoen for laptab hos disse patienter. Forsøgets resultater får således stor betydning for, om remote iskæmisk prækonditionering kan forbedre mikrokirurgisk rekonstruktion hos de meget syge patienter med hoved-halcancer.

Praktisk gennemførlighed
Forsøgets praktiske gennemførlighed er sikret ved, at de nødvendige rammer er tilstede både i forhold til inklusion af forsøgspersoner og udførsel af laboratorieanalyser. Der er i 2014 foretaget 58 rekonstruktioner med fri lap ved Plastikkirurgisk Afdeling Z, AUH. Standard laboratorieanalyser udføres ved Klinisk Biokemisk Afdeling, AUH og forskningsanalyserne udføres i forskningsenheden ved Center for Hæmofili og Trombose, Klinisk Biokemisk Afdeling, AUH.

Offentliggørelse af resultater
Uanset om resultaterne er positive, negative eller inkonklusive vil de blive publiceret i internationale medicinske tidsskrifter. Følgende publikation forventes:
Effekten af remote iskæmisk prækonditionering ved mikrokirurgisk hoved-halsrekonstruktion – randomiseret klinisk forsøg. *AE Krag, BJ Kiil, CL Hvas, GE Toft, AM Hvas.*

Tidsplan
Forsøget gennemføres som del af et MD/PhD-studium ved Health, Aarhus Universitet med indskrivning på den prægraduate del d. 1.9.2015, påbegyndelse af den postgraduate del d. 1.2.2018 og aflevering af afhandling d. 31.1.2020. Forsøgspersonerne forventes inkluderet ultimo 2018, og analysearbejdet foretages løbende.

Referencer

1. Selber JC, Angel Soto-Miranda M, Liu J, Robb G. The survival curve: Factors impacting the outcome of free flap take-backs*. Plast Reconstr Surg*. 2012;130:105-113.

2. Khouri RK, Cooley BC, Kunselman AR et al. A prospective study of microvascular free-flap surgery and outcome*. Plast Reconstr Surg*. 1998;102:711-721.

3. Culliford AT,4th, Spector J, Blank A, Karp NS, Kasabian A, Levine JP. The fate of lower extremities with failed free flaps: A single institution's experience over 25 years*. Ann Plast Surg*. 2007;59:18-21; discussion 21-2.

4. Olsson E, Svartling N, Asko-Seljavaara S, Lassila R. Activation of coagulation and fibrinolysis during reconstructive microsurgery in patients with cancer*. Microsurgery*. 2001;21:208-213.

5. Murry CE, Jennings RB, Reimer KA. Preconditioning with ischemia: A delay of lethal cell injury in ischemic myocardium*. Circulation*. 1986;74:1124-1136.

6. Kharbanda RK, Mortensen UM, White PA et al. Transient limb ischemia induces remote ischemic preconditioning in vivo*. Circulation*. 2002;106:2881-2883.

7. Schmidt MR, Smerup M, Konstantinov IE et al. Intermittent peripheral tissue ischemia during coronary ischemia reduces myocardial infarction through a KATP-dependent mechanism: First demonstration of remote ischemic perconditioning*. Am J Physiol Heart Circ Physiol*. 2007;292:H1883-90.

8. Kerendi F, Kin H, Halkos ME et al. Remote postconditioning. brief renal ischemia and reperfusion applied before coronary artery reperfusion reduces myocardial infarct size via endogenous activation of adenosine receptors*. Basic Res Cardiol*. 2005;100:404-412.

9. Bøtker HE, Kharbanda R, Schmidt MR et al. Remote ischaemic conditioning before hospital admission, as a complement to angioplasty, and effect on myocardial salvage in patients with acute myocardial infarction: A randomised trial. *The Lancet*;375:727-734.

10. Hougaard KD, Hjort N, Zeidler D et al. Remote ischemic perconditioning as an adjunct therapy to thrombolysis in patients with acute ischemic stroke: A randomized trial. *Stroke*. 2013.

11. Dickson EW, Reinhardt CP, Renzi FP, Becker RC, Porcaro WA, Heard SO. Ischemic preconditioning may be transferable via whole blood transfusion: Preliminary evidence. *J Thromb Thrombolysis*. 1999;8:123-129.

12. Lim SY, Yellon DM, Hausenloy DJ. The neural and humoral pathways in remote limb ischemic preconditioning. *Basic Res Cardiol*. 2010;105:651-655.

13. Addison PD, Neligan PC, Ashrafpour H et al. Noninvasive remote ischemic preconditioning for global protection of skeletal muscle against infarction. *Am J Physiol Heart Circ Physiol*. 2003;285:H1435-43.

14. Warzecha Z, Dembinski A, Ceranowicz P et al. Influence of ischemic preconditioning on blood coagulation, fibrinolytic activity and pancreatic repair in the course of caerulein-induced acute pancreatitis in rats. *J Physiol Pharmacol*. 2007;58:303-319.

15. Ropcke D, Hjortdal V, Toft G, Jensen M, Kristensen S. Remote ischemic preconditioning reduces thrombus formation in the rat. *J Thromb Haemost*. 2013.

16. Pedersen CM, Cruden NL, Schmidt MR et al. Remote ischemic preconditioning prevents systemic platelet activation associated with ischemia-reperfusion injury in humans. *J Thromb Haemost*. 2011;9:404-407.

17. Mounsey RA, Pang CY, Boyd JB, Forrest C. Augmentation of skeletal muscle survival in the latissimus dorsi porcine model using acute ischemic preconditioning. *J Otolaryngol*. 1992;21:315-320.

18. Kuntscher MV, Schirmbeck EU, Menke H, Klar E, Gebhard MM, Germann G. Ischemic preconditioning by brief extremity ischemia before flap ischemia in a rat model. *Plast Reconstr Surg*. 2002;109:2398-2404.

19. Kuntscher MV, Kastell T, Sauerbier M, Nobiling R, Gebhard MM, Germann G. Acute remote ischemic preconditioning on a rat cremasteric muscle flap model. *Microsurgery*. 2002;22:221-226.

20. Moses MA, Addison PD, Neligan PC et al. Mitochondrial KATP channels in hindlimb remote ischemic preconditioning of skeletal muscle against infarction. *Am J Physiol Heart Circ Physiol*. 2005;288:H559-67.

21. Restifo RJ, Thomson JG. The preconditioned TRAM flap: Preliminary clinical experience. *Ann Plast Surg*. 1998;41:343-347.

22. Kraemer R, Lorenzen J, Kabbani M et al. Acute effects of remote ischemic preconditioning on cutaneous microcirculation--a controlled prospective cohort study. *BMC Surg*. 2011;11:32-2482-11-32.

23. Kolbenschlag J, Sogorski A, Harati K et al. Upper extremity ischemia is superior to lower extremity ischemia for remote ischemic conditioning of antero-lateral thigh cutaneous blood flow*. Microsurgery*. 2014.

24. Chubb DP, Taylor GI, Ashton MW. True and 'choke' anastomoses between perforator angiosomes: Part II. dynamic thermographic identification*. Plast Reconstr Surg*. 2013;132:1457-1464.

25. Rubak P, Villadsen K, Hvas AM. Reference intervals for platelet aggregation assessed by multiple electrode platelet aggregometry*. Thromb Res*. 2012;130:420-423.
