# Supplementary material for: Effect of remote ischemic preconditioning on hemostasis and fibrinolysis in head and neck cancer surgery: A randomized controlled trial
Source: PLoS One. 2019 Jul 8;14(7):e0219496. doi: 10.1371/journal.pone.0219496 (PMC6613699; doi:10.1371/journal.pone.0219496)
Supplement: S4 File — (PDF) [file pone.0219496.s004.pdf]

This document is an English translation of the 2nd modified trial protocol submitted to the Central Denmark Region's Ethics Committee on December 17, 2015.

Remote Ischemic Preconditioning in Head and Neck Cancer Reconstruction - A Randomized Controlled Trial

Aim

The aim of the trial is to investigate if remote ischemic preconditioning improves the outcome of microsurgical reconstruction.

Literature review

Overview of systematic literature review performed in PubMed/MEDLINE:

Search terms (April 7, 2015)	*Identified references:*
"Ischemic Preconditioning"[Mesh] AND "Hemostasis"[Mesh]	20
"Blood Coagulation"[Mesh] AND "Ischemic Preconditioning"[Mesh]	4
"Ischemic Preconditioning"[Mesh] AND "Fibrinolysis"[Mesh]	2
"Thrombosis"[Mesh] AND "Ischemic Preconditioning"[Mesh]	20
ischemic preconditioning coagulation	35
"Surgical Flaps"[Mesh] AND "Ischemic Preconditioning"[Mesh]	78
"Surgical Flaps"[Mesh] AND "Ischemic Preconditioning"[Mesh] AND "Hemostasis"[Mesh]	0
"Surgical Flaps"[Mesh] AND "Ischemic Preconditioning"[Mesh] AND "Thrombosis"[Mesh]	0

Additional literature was identified through review of reference lists from relevant publications.

Background

In microsurgical reconstruction, the patient's own tissue is applied for reconstruction of defects following tumor resection or trauma. Blocks of tissue are harvested from a donor site and transferred to the defect, where the blood supply is restored by microvascular anastomoses under the operating microscope. These tissue blocks are termed 'free flaps'. For example, the fibula bone with a skin paddle from the lower leg is applied for reconstruction of the jawbone and oral cavity following resection of oral cancer.

Thrombosis to the vascular anastomoses and microcirculatory intravascular thrombosis are the main reasons for total flap necrosis. Total flap necrosis occurs in 1-9 per cent of cases with devastating consequences for the patient [1-3]. Therefore, research aimed at reducing the risk of thrombosis in free flap reconstruction is essential.

The surgical procedure results in a hypercoagulable state, endothelial dysfunction, and altered blood flow in the patient's circulation during free flap reconstruction. It is well known, that oncologic patients are in a hypercoagulable state with an increased risk of thrombosis [4]. Ischemia-reperfusion injury to the flap, and the suture line of the anastomoses, are the main reasons for endothelial dysfunction in the flap. Furthermore, stasis and hypoxia in the patient's circulation contribute to endothelial dysfunction. These changes in the coagulation system result in an increased risk of thrombosis and have major impact on the outcome of free flap reconstruction.

Ischemic preconditioning, where brief periods of ischemia and reperfusion are induced prior to prolonged ischemia, has been shown to attenuate ischemia-reperfusion injury of organs subject to acute ischemia [5]. Ischemic preconditioning can either be applied locally on the organ prior to ischemia, or anatomically remote by ischemic preconditioning of tissue separate from the target organ, termed remote ischemic preconditioning [6]. Furthermore, remote ischemic perconditioning, applied during target organ ischemia, and remote ischemic postconditioning, applied at reperfusion of the ischemic organ, have been described as methods to reduce ischemia-reperfusion injury [7-8]. Randomized controlled trials with remote ischemic perconditioning as an adjunct to revascularization in patients with myocardial infarction or ischemic stroke have shown promising results [9-10].

The mechanism behind remote ischemic conditioning is unknown, but the research has focused on a humoral response or a neurogenic response [11-13]. Furthermore, effects involving the immune system and coagulation system have been investigated [14-16]. Ischemic preconditioning and remote ischemic preconditioning of experimental free flaps have been shown to increase flap survival and attenuate ischemia-reperfusion injury [13,17-20]. However, just one clinical case series with six preconditioned abdominal flaps for breast reconstruction has been published. And two clinical studies with healthy subjects have shown, that remote ischemic conditioning improves the cutaneous microcirculation at a free flap donor site [21-23]. Hence, no randomized controlled trials have been conducted to show the efficacy of remote ischemic conditioning in free flap reconstruction.

Research team

This randomized controlled trial is performed at the Department of Plastic Surgery and Center for Hemophilia and Thrombosis, Department of Clinical Biochemistry at Aarhus University Hospital (AUH), Aarhus, Denmark. The trial is part of a Ph.D. project at the Graduate School of Health at Aarhus University.

The research team consists of:

- Study chair and sponsor: Professor Anne-Mette Hvas, M.D., Ph.D., Center for Hemophilia and Thrombosis, Department of Clinical Biochemistry, AUH.
- M.D./Ph.D.-student: Andreas Engel Krag, Center for Hemophilia and Thrombosis, Department of Clinical Biochemistry, AUH.
- Birgitte Jul Kiil, M.D., Department of Plastic Surgery, AUH.
- Christine Lodberg Hvas, M.D., Ph.D., Department of Anesthesiology, AUH.

Study aims

1) To investigate if remote ischemic preconditioning attenuates platelet aggregation in head and neck cancer patients undergoing microsurgical reconstruction.

2) To investigate if remote ischemic preconditioning induces fibrinolysis in head and neck cancer patients undergoing microsurgical reconstruction.

Hypotheses

1) Remote ischemic preconditioning attenuates platelet aggregation.

2) Remote ischemic preconditioning increases fibrinolysis.

Study participants, methods and materials

Design: Randomized controlled trial.

Study intervention: Included patients will be randomized in the operating room after induction of general anesthesia to remote ischemic preconditioning or no remote ischemic preconditioning (sham). Patients are blinded to the study intervention. Remote ischemic preconditioning will be carried out by upper extremity ischemia using an inflatable tourniquet. Four cycles of 5 min ischemia followed by 5 min of reperfusion will be administered. In patients randomized to sham, the tourniquet will be applied to the patient, but not inflated.

Eligibility:
Inclusion criteria:
- At least 18 years of age.
- Ability to give informed consent.
- Will undergo resection of a malignant tumor in the head and neck region immediately followed by microsurgical reconstruction with a single free flap at the Department of Plastic Surgery, AUH.

Exclusion criteria: Arterial or venous thromboembolism in the previous three months.

Outcomes:
Biochemical data:
- Primary hemostasis: Platelet count and platelet aggregation (Multiplate®).
- Secondary hemostasis: aPTT, INR, antithrombin, and thrombin-antithrombin complex.
- Fibrinolysis: Fibrin clot lysis, fibrin d-dimer, prothrombin fragment 1+2, plasminogen activator inhibitor-1, tissue-plasminogen activator, plasminogen, and thrombin-activatable fibrinolysis inhibitor.
- Global hemostasis: Thrombin generation.
- Systemic inflammation: C-reactive protein, leukocytes, fibrinogen, interleukin-6, and von Willebrand factor (antigen).

Blood samples will be drawn at:
1. Just before induction of general anesthesia.
2. At randomization (intraoperatively).
3. 3 h after RIPC/sham.
4. 6 h after RIPC/sham.
5. 06:30 AM on the 1st postoperative day.

Clinical data:
- Previous treatment for head and neck cancer
- Co-morbidities
- Medication
- Course of hospitalization
- Re-operations
- Morbidity and mortality

Follow-up for clinical data is 30 days postoperatively. Data will be collected from review of patients' electronic medical records and postoperative visits to the Plastic Surgery Clinic, AUH.

Obtaining informed consent
Potential study participants will be encountered in the multidisciplinary Head and Neck Cancer Clinic, AUH, where newly referred head and neck cancer patients are evaluated for surgery by head and neck surgeons, plastic surgeons, maxillofacial surgeons, and oncologists. Patients who are scheduled for surgery, and who meet the inclusion criteria, will receive verbal and written information about the clinical trial from Dr. Birgitte Jul Kiil, Dr. Gete Toft Eschen, Dr. Hans Henrik Nielsen, or M.D./Ph.D. student Andreas Engel Krag. This conservation will take place in a private clinic room with no interruptions, and the patient's companions are encouraged to join the conversation.
Informed consent for trial participation will either be obtained immediately, or the patient is offered time for deliberation until the day of admission before surgery. Under normal circumstances the patient will be scheduled for surgery within 14 days from their first visit to the Head and Neck Cancer Clinic.

Biobanks
Trial biobank: From each blood sample, 12 mL blood will be analyzed immediately, and the remaining blood will be stored as plasma at -80 degrees Celsius for batch analyses. These batch analyses will be performed before the project end date on January 31, 2020. This biobank will be established at the Department of Clinical Biochemistry, AUH.
Biobank for future research: Consent will be obtained from study participants for collection of extra blood at each blood sample time point, which can be used in future, unspecified research. Study participants who decline donating blood to this biobank for future research can still participate in the clinical trial. Collected material in the biobank for future research can only be used in future research projects after approval from the Ethics Committee and approval from the trial research team. The material will be stored at the Department of Clinical Biochemistry, AUH, for 15 years. After this time point leftover material will be destroyed.

Sample size calculation and statistics
An alpha (two-sided) of 0.05 and power (1 – beta) of 90 % have been applied for the sample size calculation. The primary endpoint is platelet aggregation measured by Multiplate®. From a previous study, the mean of the area under the curve for collagen-induced platelet aggregation was found to be 815 AU x min with standard deviation 130 AU x min [24]. We wish to detect a difference of at least 125 AU x min, which leads to inclusion of 23 patients in each of the two groups. We have chosen to include 30 patients in each group.
The difference in biochemical markers between groups over time will be tested using ANOVA models. The difference between groups at specific time points will be tested with the unpaired t-test for parametric data or the Wilcoxon rank-sum test for non-parametric data.

Ethical considerations
Study permission will be obtained from the Central Denmark Region's Ethics Committee and the Danish Data Protection Agency. The Declaration of Helsinki will be followed in all aspects. Risks or adverse effects related to remote ischemic preconditioning have not previously been reported.
Blood samples obtained during the day of surgery will be collected from an arterial cannula inserted by the anesthesiologist used for blood pressure monitoring during and after surgery. The blood sample obtained on the first postoperative day will be collected

from that same arterial cannula, or from a central venous line or venipuncture if the arterial cannula has been removed. There is a small risk of developing hematoma or infection after venipuncture.
At each blood sample 28.8 mL blood are collected, or 36.4 mL blood if the study participant has given consent to the biobank for future research. Hence, 144 mL or 182 mL blood will be collected in total from each patient participating in the trial. The research team has assessed that collecting this total blood volume does not impose a risk on study participants.
All adverse effects related to the study intervention will be reported to the Ethics Committee.
The individual study participant will not directly benefit from participation in the trial.
It is the research team's opinion that new knowledge generated by this trial counterbalances the potential inconveniences and risks related to trial participation.

Premature trial discontinuation
The individual study participant will be excluded from the trial if he/she withdraws consent. The research team does not anticipate any circumstances where the whole trial might be discontinued.

Data management
Handling, analysis, and filing of data will be in compliance with the Danish Data Protection Act and regulations from the Danish Data Protection Agency. Identifiable data will be anonymized at the end of the trial. Data will be filed for 15 years after the end of the trial at the Department of Clinical Biochemistry, AUH.

Disclosure of protected health information to the research team
After informed consent has been obtained, clinical data will be collected during the trial from the patient's electronic medical records. Only data described under "Study participants, methods and materials" will be collected.

Funding
The research team has initiated the trial. Aarhus University has granted a M.D./Ph.D. fellowship to Andreas Engel Krag. Running costs will be covered by grants from private foundations.
Study participants will not receive compensation for participating in the trial.

Perspectives
Total free flap necrosis occurs in up to 10% of cases, with potentially fatal consequences for patients who received head and neck reconstruction. The results of the trial will show if remote ischemic preconditioning reduces the risk of thrombosis, and thereby free flap loss in this group of patients.

Feasibility
Feasibility is secured in terms of patient recruitment and laboratory expertise. In 2013, 58 patients received microsurgical reconstruction at the Department of Plastic Surgery, AUH. The laboratory analyses will be performed at the accredited clinical laboratory and research unit at the Department of Clinical Biochemistry, AUH.

Publications
The results of the trial, whether positive, negative, or inconclusive, will be published in international peer-reviewed scientific journals.

Time line
The study period will be from September 1, 2015 to January 31, 2020.
All patients are expected to be included in the clinical trial between 2015 and 2018, and laboratory analyses will be performed continually during the study period.

References
1. Selber JC, Angel Soto-Miranda M, Liu J, Robb G. The survival curve: Factors impacting the outcome of free flap take-backs. Plast Reconstr Surg. 2012;130:105-113.
2. Khouri RK, Cooley BC, Kunselman AR et al. A prospective study of microvascular free-flap surgery and outcome. Plast Reconstr Surg. 1998;102:711-721.
3. Culliford AT,4th, Spector J, Blank A, Karp NS, Kasabian A, Levine JP. The fate of lower extremities with failed free flaps: A single institution's experience over 25 years. Ann Plast Surg. 2007;59:18-21; discussion 21-2.
4. Olsson E, Svartling N, Asko-Seljavaara S, Lassila R. Activation of coagulation and fibrinolysis during reconstructive microsurgery in patients with cancer. Microsurgery. 2001;21:208-213.
5. Murry CE, Jennings RB, Reimer KA. Preconditioning with ischemia: A delay of lethal cell injury in ischemic myocardium. Circulation. 1986;74:1124-1136.
6. Kharbanda RK, Mortensen UM, White PA et al. Transient limb ischemia induces remote ischemic preconditioning in vivo. Circulation. 2002;106:2881-2883.
7. Schmidt MR, Smerup M, Konstantinov IE et al. Intermittent peripheral tissue ischemia during coronary ischemia reduces myocardial infarction through a KATP-dependent mechanism: First demonstration of remote ischemic perconditioning. Am J Physiol Heart Circ Physiol. 2007;292:H1883-90.
8. Kerendi F, Kin H, Halkos ME et al. Remote postconditioning. brief renal ischemia and reperfusion applied before coronary artery reperfusion reduces myocardial infarct size via endogenous activation of adenosine receptors. Basic Res Cardiol. 2005;100:404-412.
9. Bøtker HE, Kharbanda R, Schmidt MR et al. Remote ischaemic conditioning before hospital admission, as a complement to angioplasty, and effect on myocardial salvage in patients with acute myocardial infarction: A randomised trial. The Lancet;375:727-734.
10. Hougaard KD, Hjort N, Zeidler D et al. Remote ischemic perconditioning as an adjunct therapy to thrombolysis in patients with acute ischemic stroke: A randomized trial. Stroke. 2013.
11. Dickson EW, Reinhardt CP, Renzi FP, Becker RC, Porcaro WA, Heard SO. Ischemic preconditioning may be transferable via whole blood transfusion: Preliminary evidence. J Thromb Thrombolysis. 1999;8:123-129.
12. Lim SY, Yellon DM, Hausenloy DJ. The neural and humoral pathways in remote limb ischemic preconditioning. Basic Res Cardiol. 2010;105:651-655.
13. Addison PD, Neligan PC, Ashrafpour H et al. Noninvasive remote ischemic preconditioning for global protection of skeletal muscle against infarction. Am J Physiol Heart Circ Physiol. 2003;285:H1435-43.
14. Warzecha Z, Dembinski A, Ceranowicz P et al. Influence of ischemic preconditioning on blood coagulation, fibrinolytic activity and pancreatic repair in the course of caerulein-induced acute pancreatitis in rats. J Physiol Pharmacol. 2007;58:303-319.
15. Ropcke D, Hjortdal V, Toft G, Jensen M, Kristensen S. Remote ischemic preconditioning reduces thrombus formation in the rat. J Thromb Haemost. 2013.

16. Pedersen CM, Cruden NL, Schmidt MR et al. Remote ischemic preconditioning prevents systemic platelet activation associated with ischemia-reperfusion injury in humans. J Thromb Haemost. 2011;9:404-407.
17. Mounsey RA, Pang CY, Boyd JB, Forrest C. Augmentation of skeletal muscle survival in the latissimus dorsi porcine model using acute ischemic preconditioning. J Otolaryngol. 1992;21:315-320.
18. Kuntscher MV, Schirmbeck EU, Menke H, Klar E, Gebhard MM, Germann G. Ischemic preconditioning by brief extremity ischemia before flap ischemia in a rat model. Plast Reconstr Surg. 2002;109:2398-2404.
19. Kuntscher MV, Kastell T, Sauerbier M, Nobiling R, Gebhard MM, Germann G. Acute remote ischemic preconditioning on a rat cremasteric muscle flap model. Microsurgery. 2002;22:221-226.
20. Moses MA, Addison PD, Neligan PC et al. Mitochondrial KATP channels in hindlimb remote ischemic preconditioning of skeletal muscle against infarction. Am J Physiol Heart Circ Physiol. 2005;288:H559-67.
21. Restifo RJ, Thomson JG. The preconditioned TRAM flap: Preliminary clinical experience. Ann Plast Surg. 1998;41:343-347.
22. Kraemer R, Lorenzen J, Kabbani M et al. Acute effects of remote ischemic preconditioning on cutaneous microcirculation--a controlled prospective cohort study. BMC Surg. 2011;11:32-2482-11-32.
23. Kolbenschlag J, Sogorski A, Harati K et al. Upper extremity ischemia is superior to lower extremity ischemia for remote ischemic conditioning of antero-lateral thigh cutaneous blood flow. Microsurgery. 2014.
24. Rubak P, Villadsen K, Hvas AM. Reference intervals for platelet aggregation assessed by multiple electrode platelet aggregometry. Thromb Res. 2012;130:420-423.
