# Supplementary material for: Effect of remote ischemic preconditioning on hemostasis and fibrinolysis in head and neck cancer surgery: A randomized controlled trial
Source: PLoS One. 2019 Jul 8;14(7):e0219496. doi: 10.1371/journal.pone.0219496 (PMC6613699; doi:10.1371/journal.pone.0219496)
Supplement: S5 File — (PDF) [file pone.0219496.s005.pdf]

QQ-PLOTS OF CONTINOUS VARIABLES WITH SKEWED DISTRIBUTION BEFORE AND AFTER LOGARITHMIC TRANSFORMATION

P-selectin

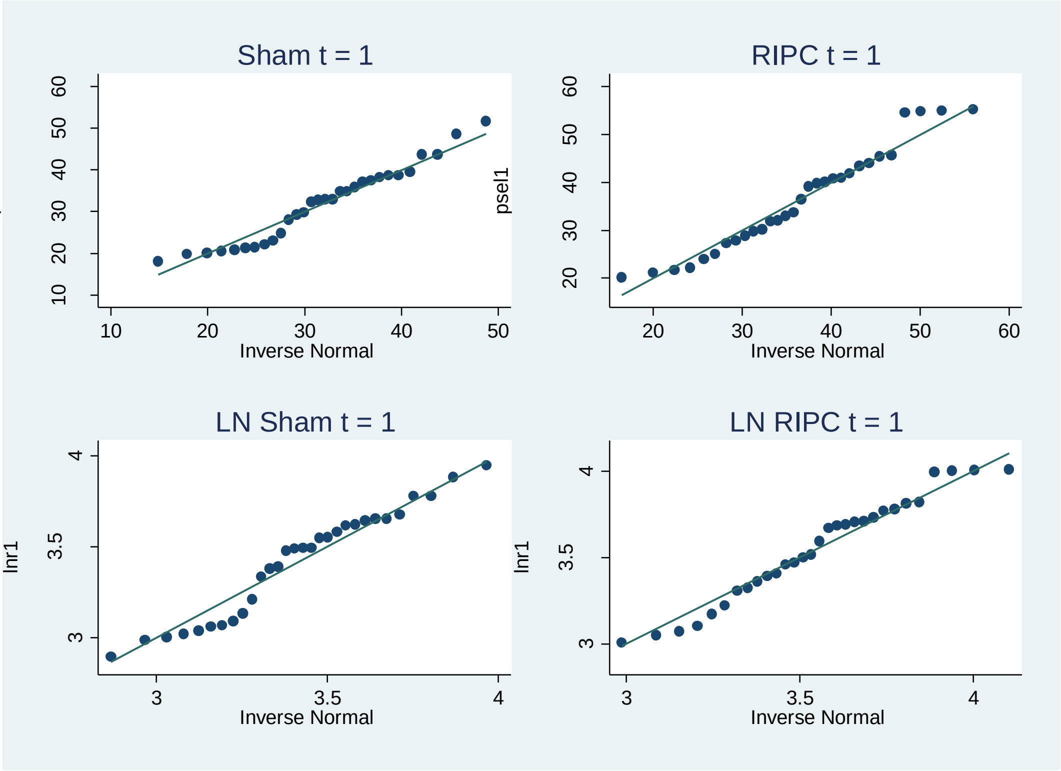

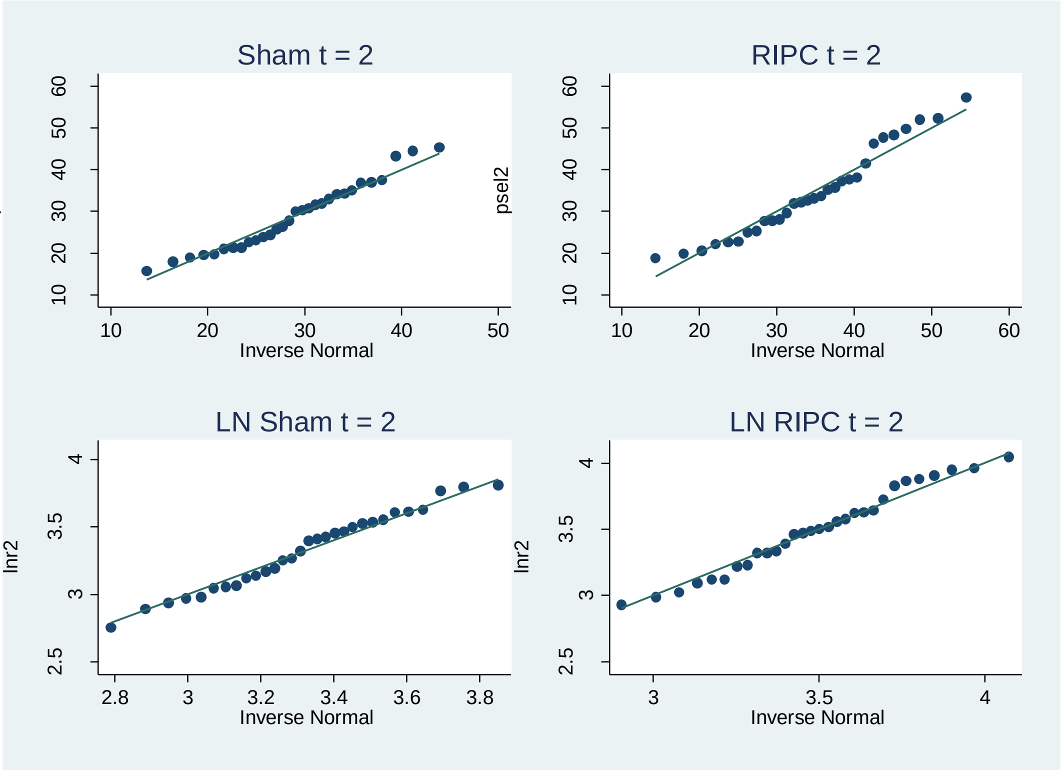

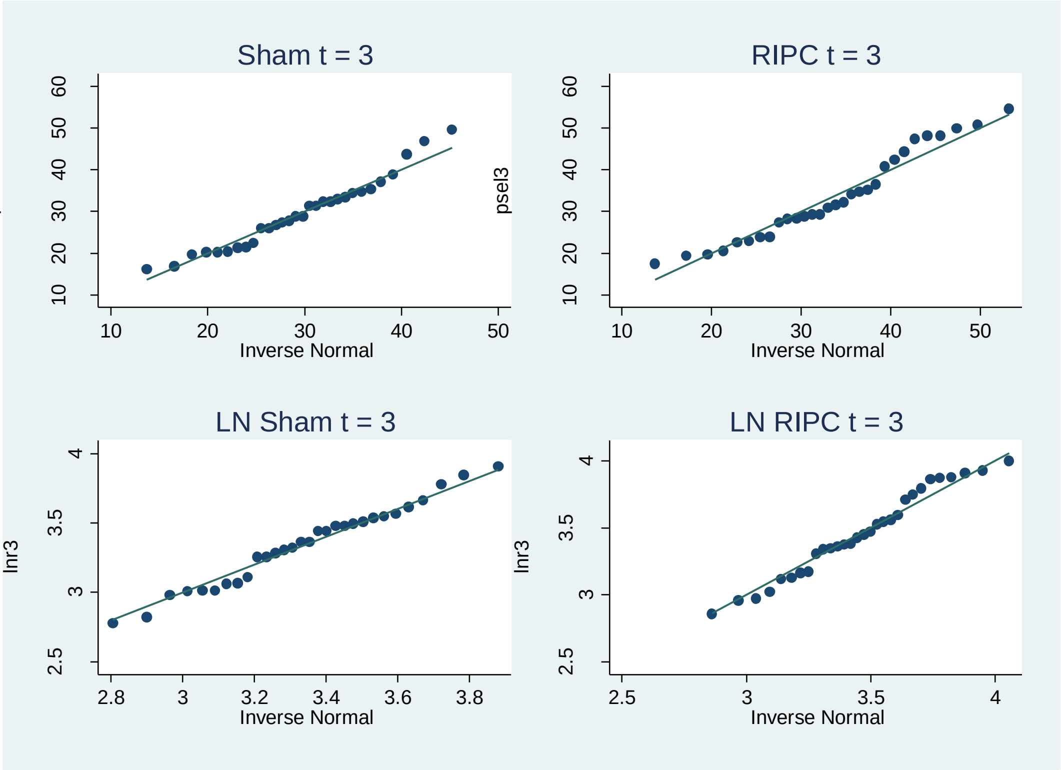
Sham t = 3
RIPC t = 3
psel3
Inverse Normal
LN Sham t = 3
LN RIPC t = 3
lnr3
Inverse Normal

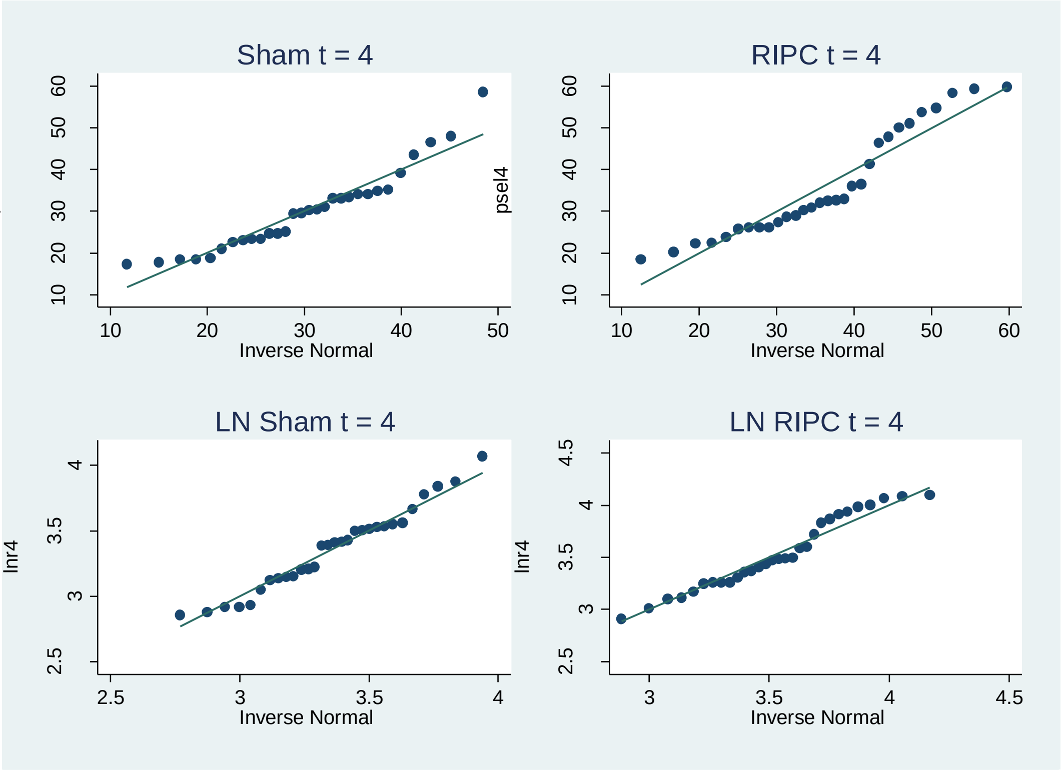
Sham t = 4
RIPC t = 4
psel4
Inverse Normal
LN Sham t = 4
LN RIPC t = 4
lnr4
Inverse Normal

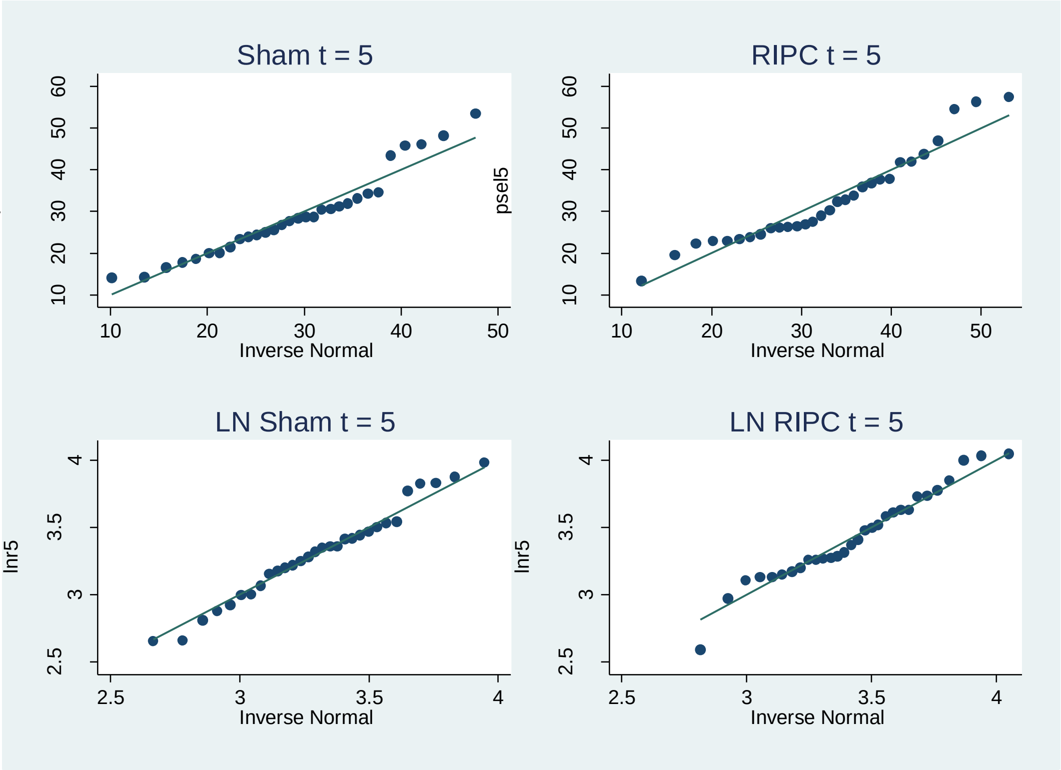

50% clot lysis time

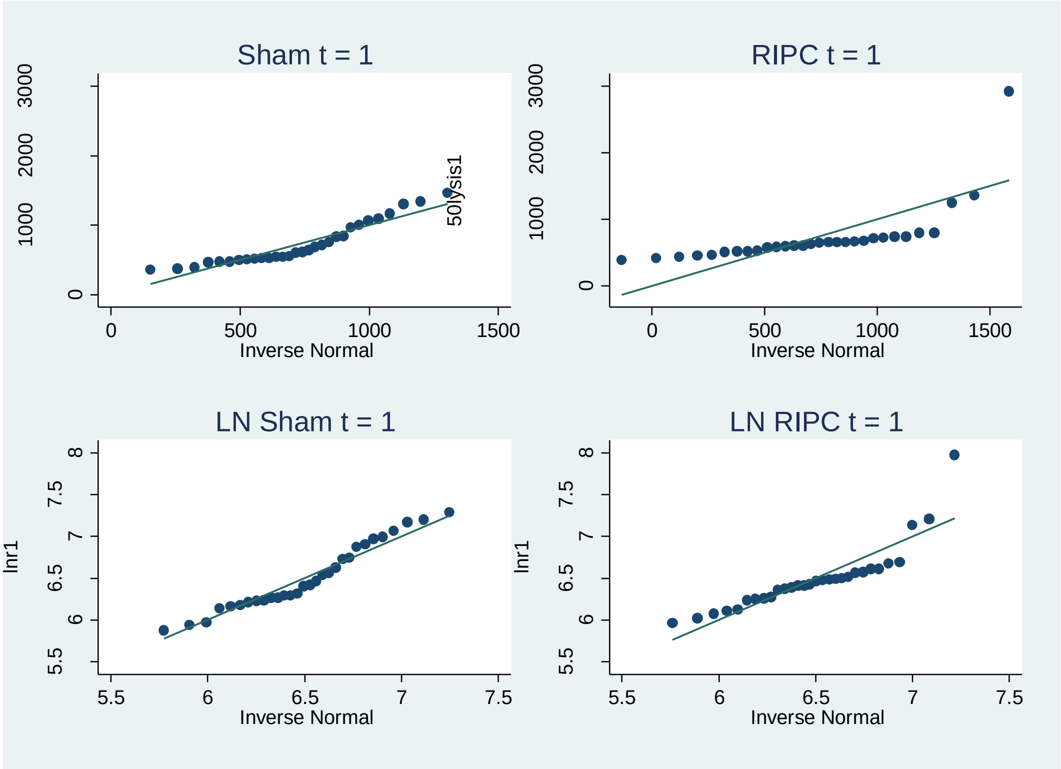

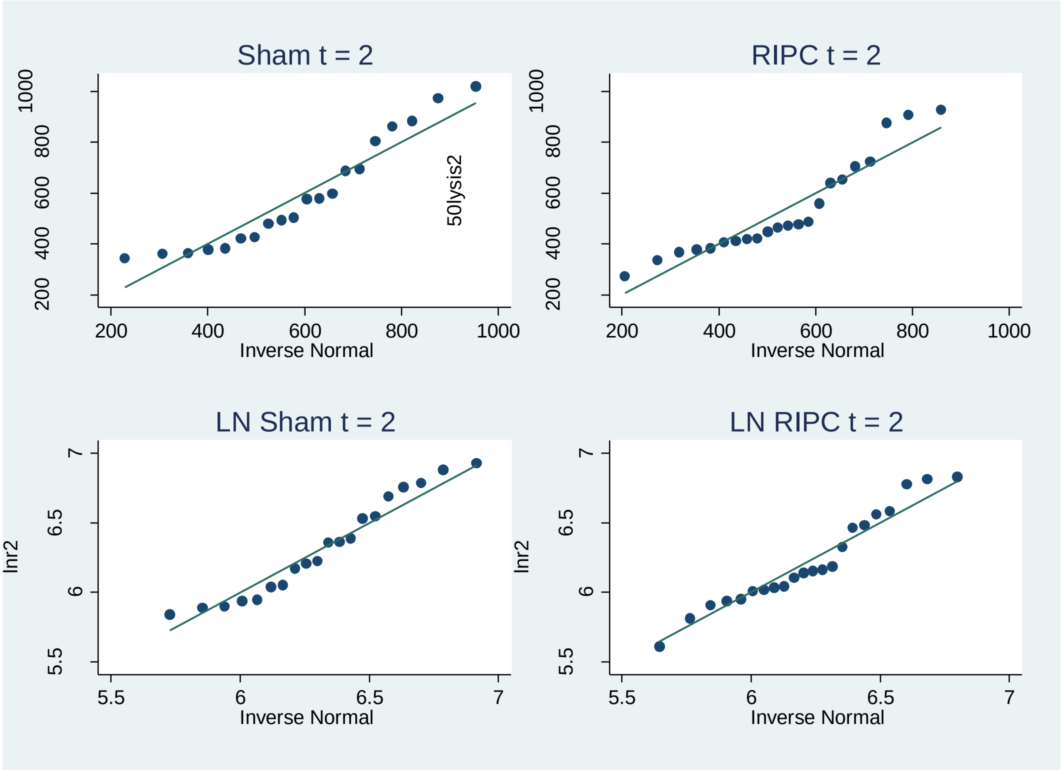

Sham t = 2
RIPC t = 2
50lysis2
Inverse Normal
Inverse Normal
LN Sham t = 2
LN RIPC t = 2
lnr2
lnr2
Inverse Normal
Inverse Normal

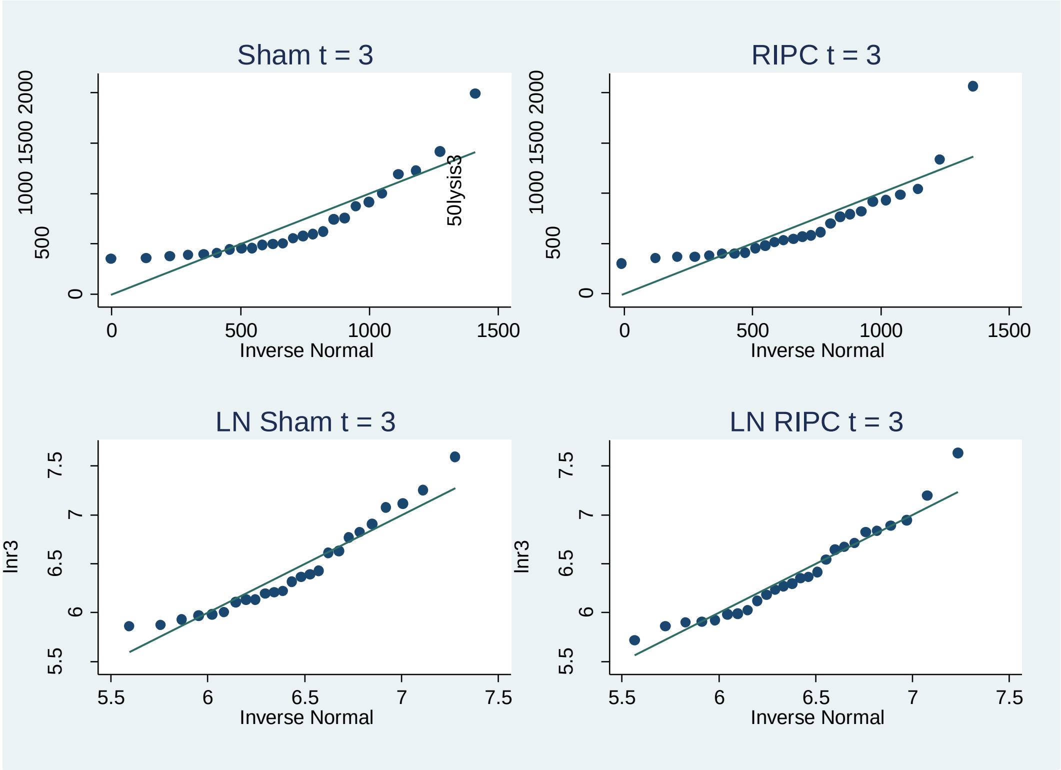

Sham t = 3
RIPC t = 3
50lysis3
Inverse Normal
Inverse Normal
LN Sham t = 3
LN RIPC t = 3
lnr3
lnr3
Inverse Normal
Inverse Normal

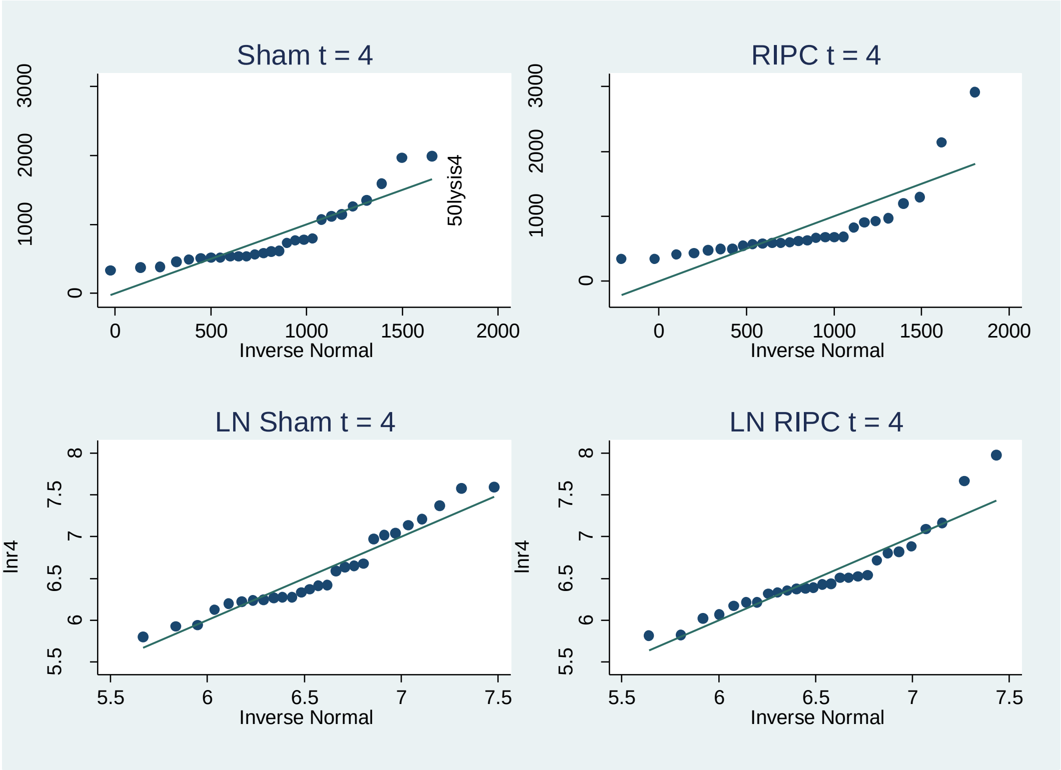
Sham t = 4
50lysis4
Inverse Normal
RIPC t = 4
Inverse Normal
LN Sham t = 4
lnr4
Inverse Normal
LN RIPC t = 4
lnr4
Inverse Normal

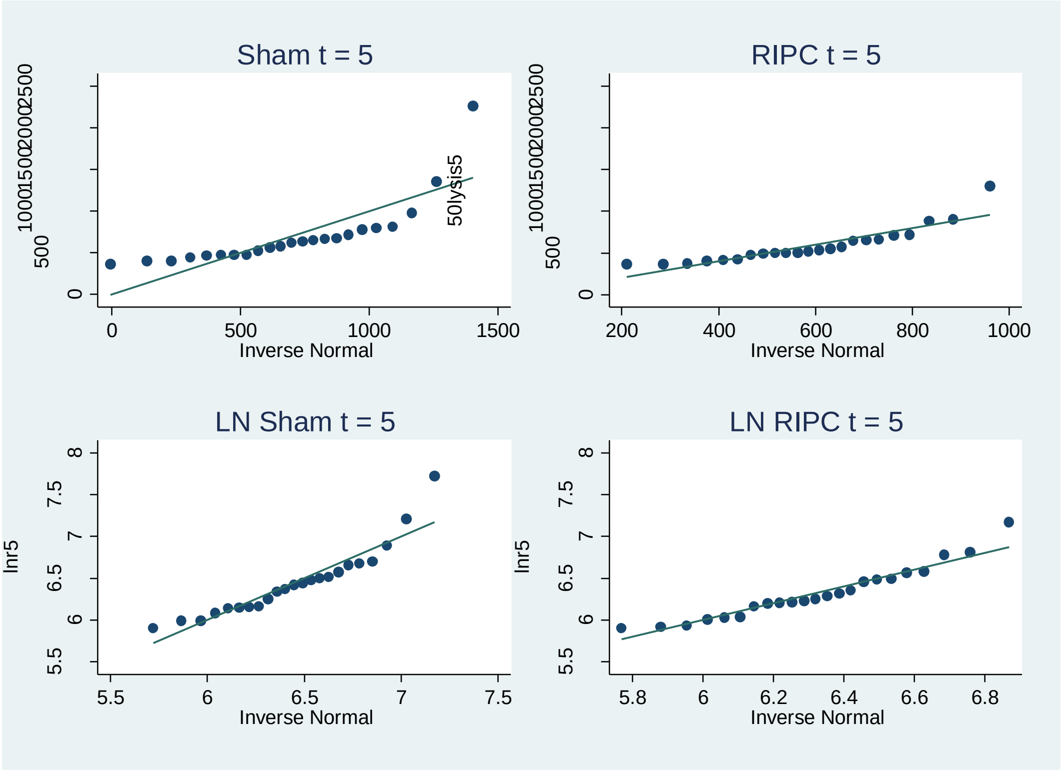
Sham t = 5
50lysis5
Inverse Normal
RIPC t = 5
Inverse Normal
LN Sham t = 5
lnr5
Inverse Normal
LN RIPC t = 5
lnr5
Inverse Normal

Clot lysis AUC

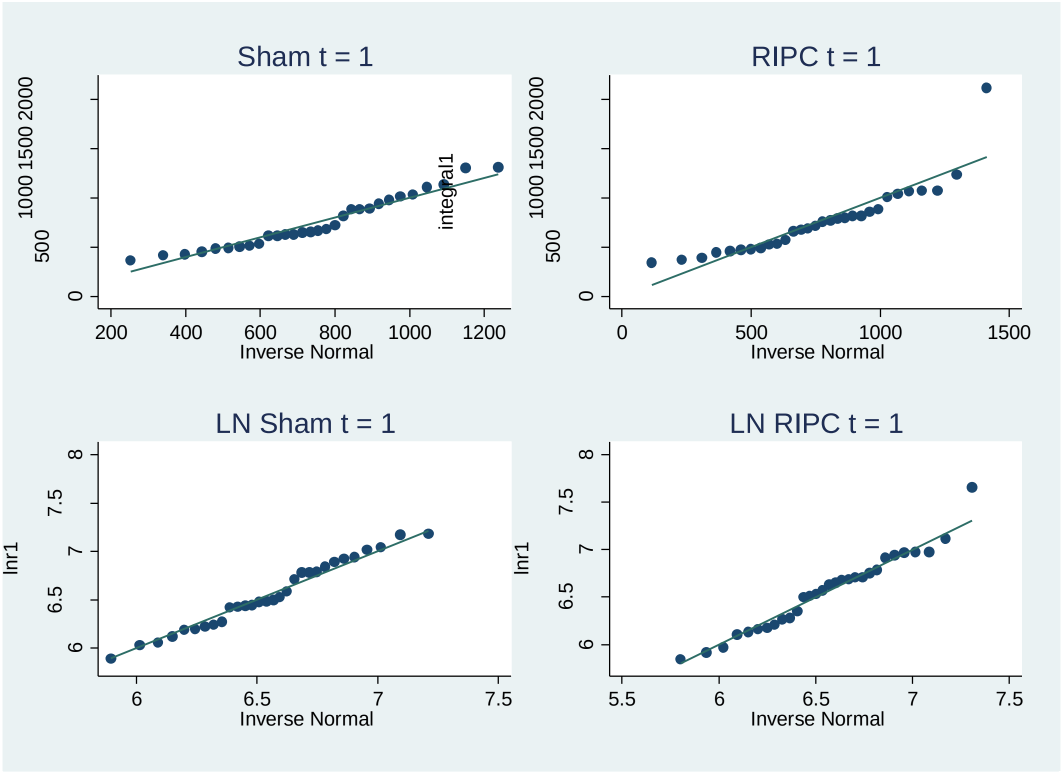

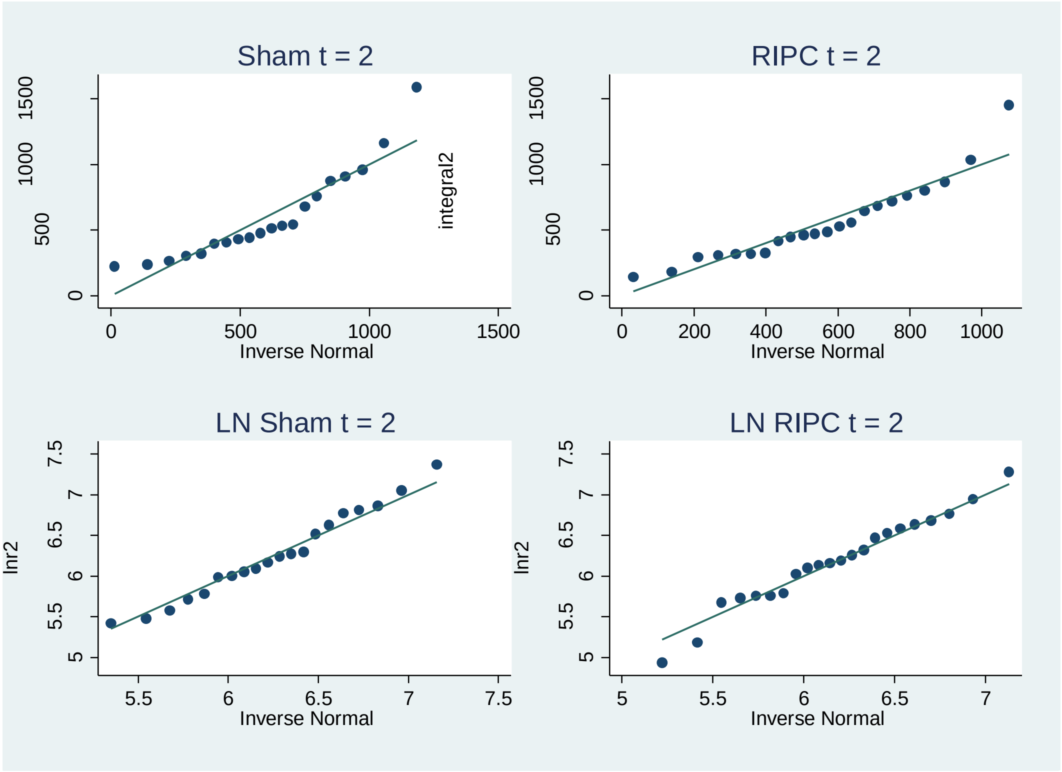

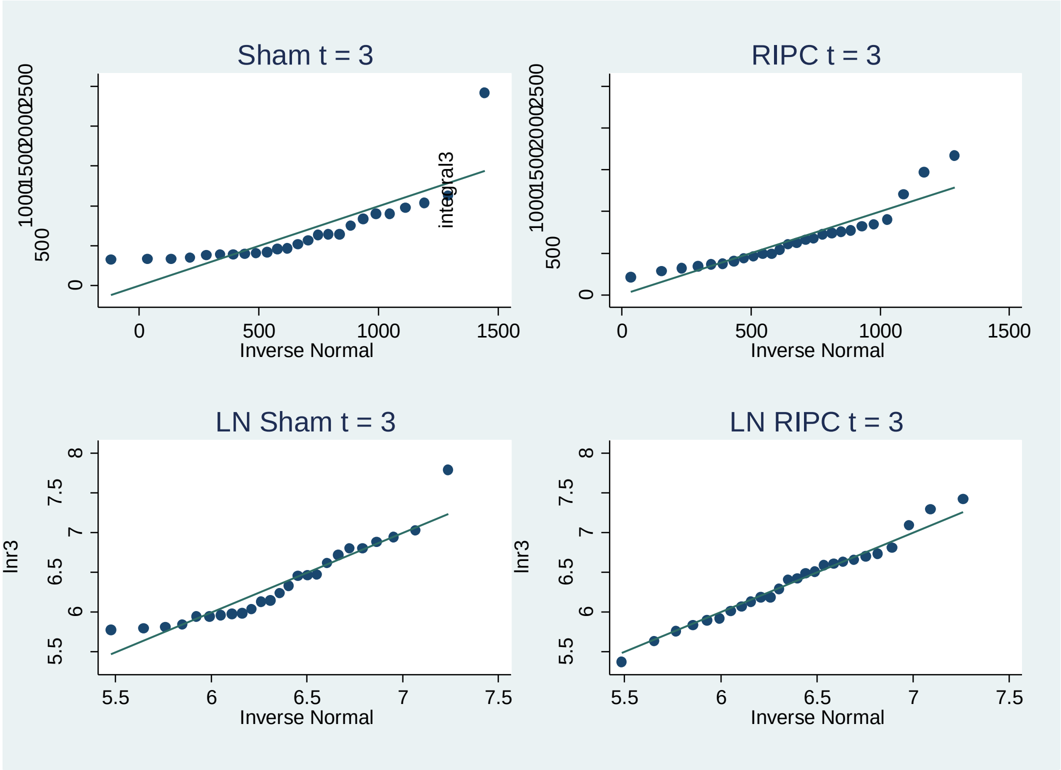
Sham t = 3
RIPC t = 3
integral3
Inverse Normal
LN Sham t = 3
LN RIPC t = 3
lnr3
Inverse Normal

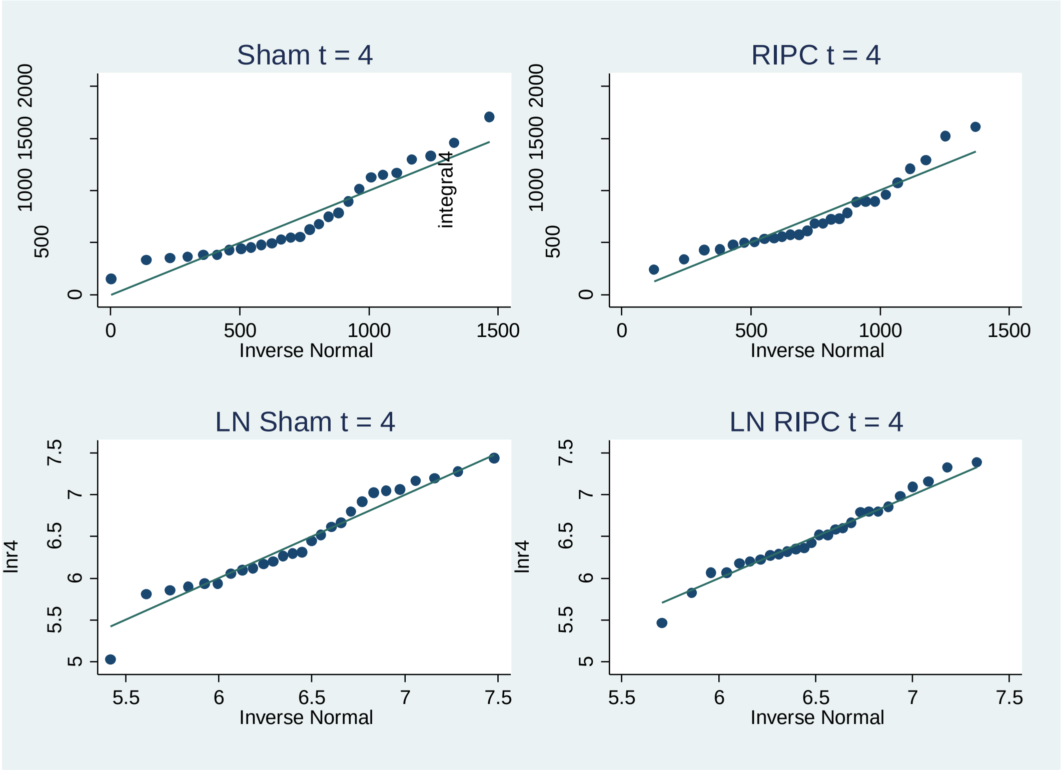
Sham t = 4
RIPC t = 4
integral4
Inverse Normal
LN Sham t = 4
LN RIPC t = 4
lnr4
Inverse Normal

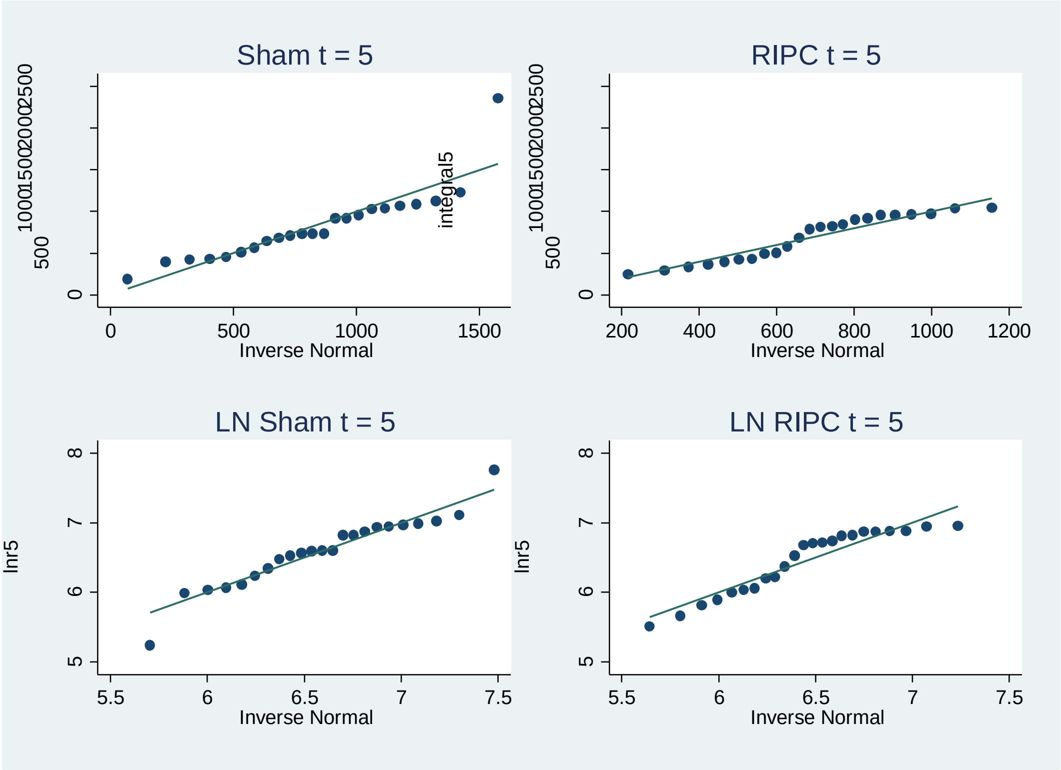

Fibrin D-dimer

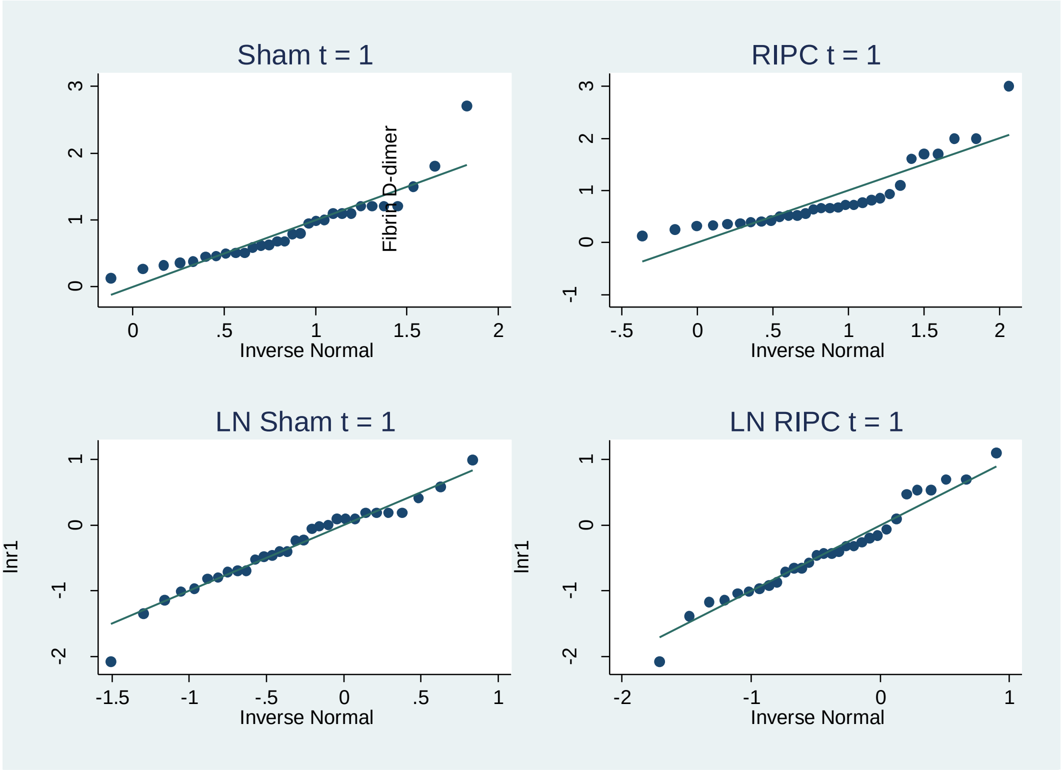

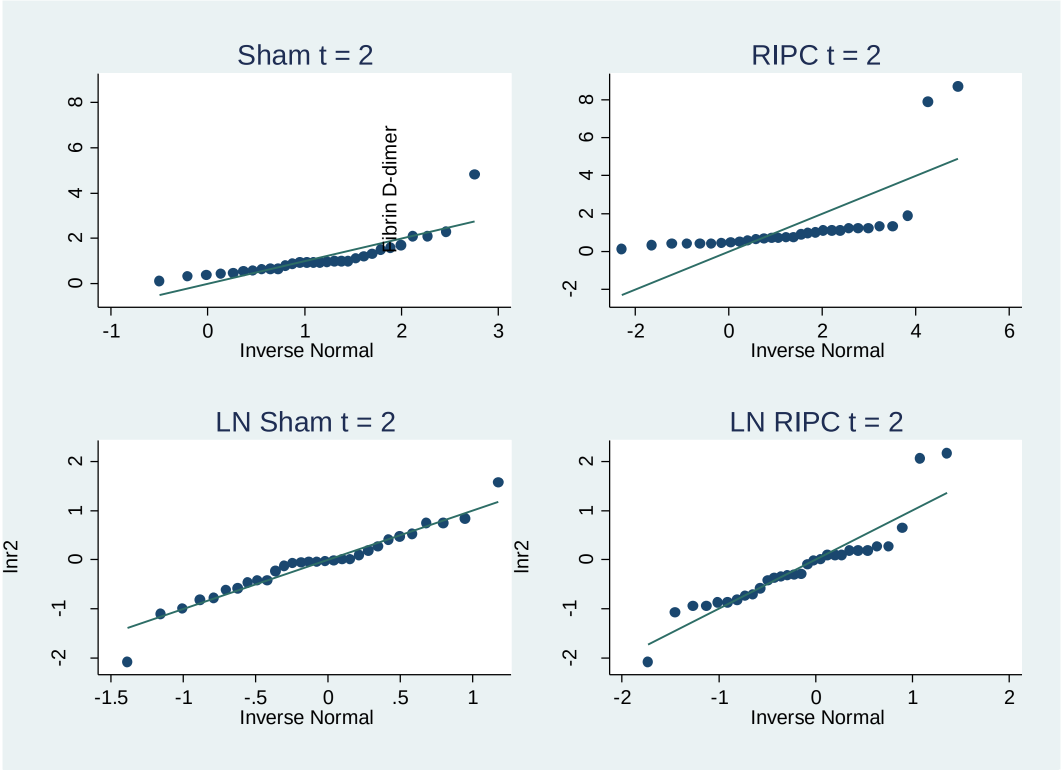
Sham t = 2
RIPC t = 2
Fibrin D-dimer
Inverse Normal
Inverse Normal
LN Sham t = 2
LN RIPC t = 2
lnr2
lnr2
Inverse Normal
Inverse Normal

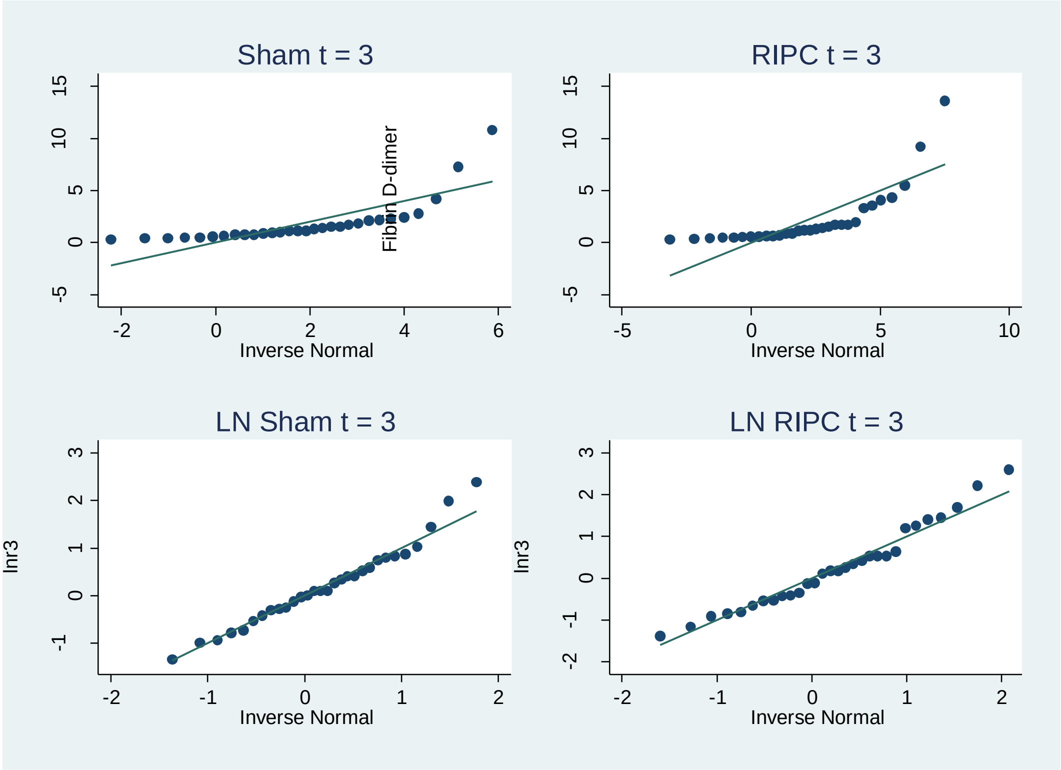
Sham t = 3
RIPC t = 3
Fibrin D-dimer
Inverse Normal
Inverse Normal
LN Sham t = 3
LN RIPC t = 3
lnr3
lnr3
Inverse Normal
Inverse Normal

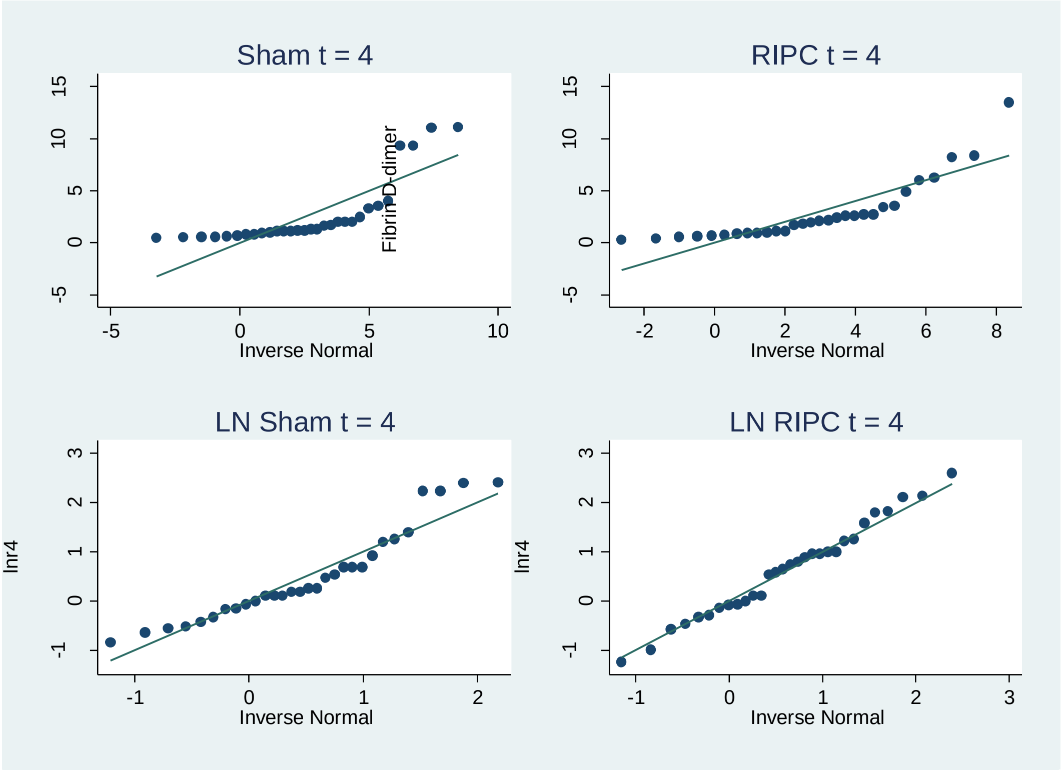
Sham t = 4
RIPC t = 4
Fibrin D-dimer
Inverse Normal
LN Sham t = 4
LN RIPC t = 4
lnr4
Inverse Normal

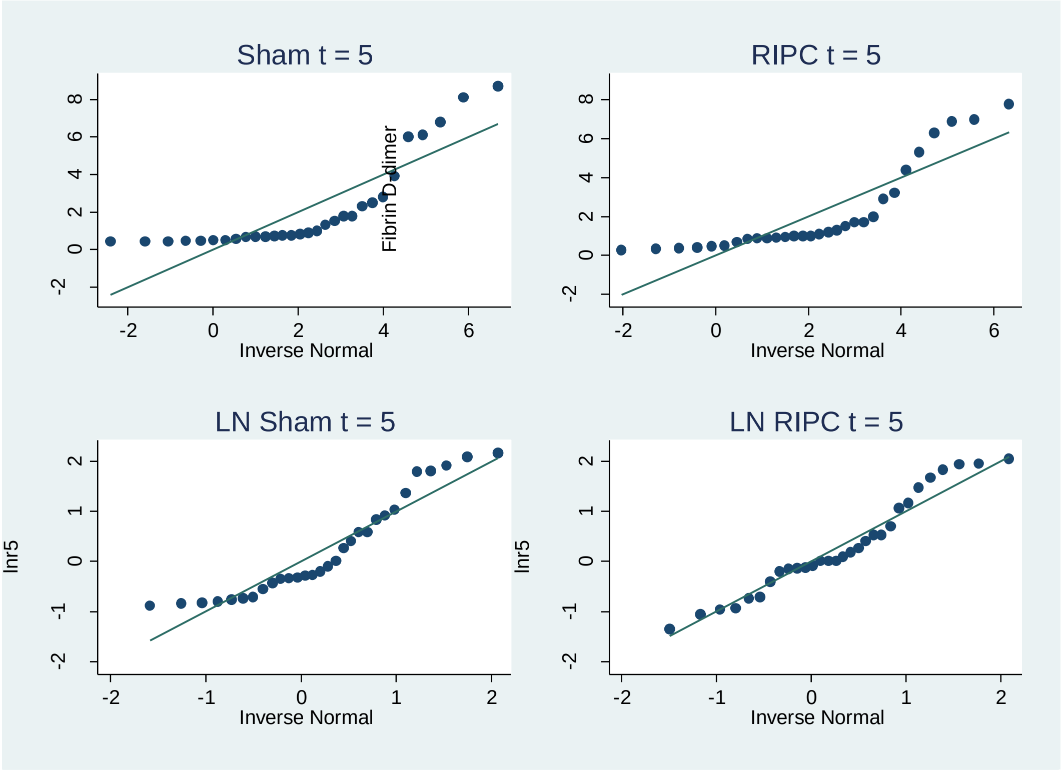
Sham t = 5
RIPC t = 5
Fibrin D-dimer
Inverse Normal
LN Sham t = 5
LN RIPC t = 5
lnr5
Inverse Normal

tPA

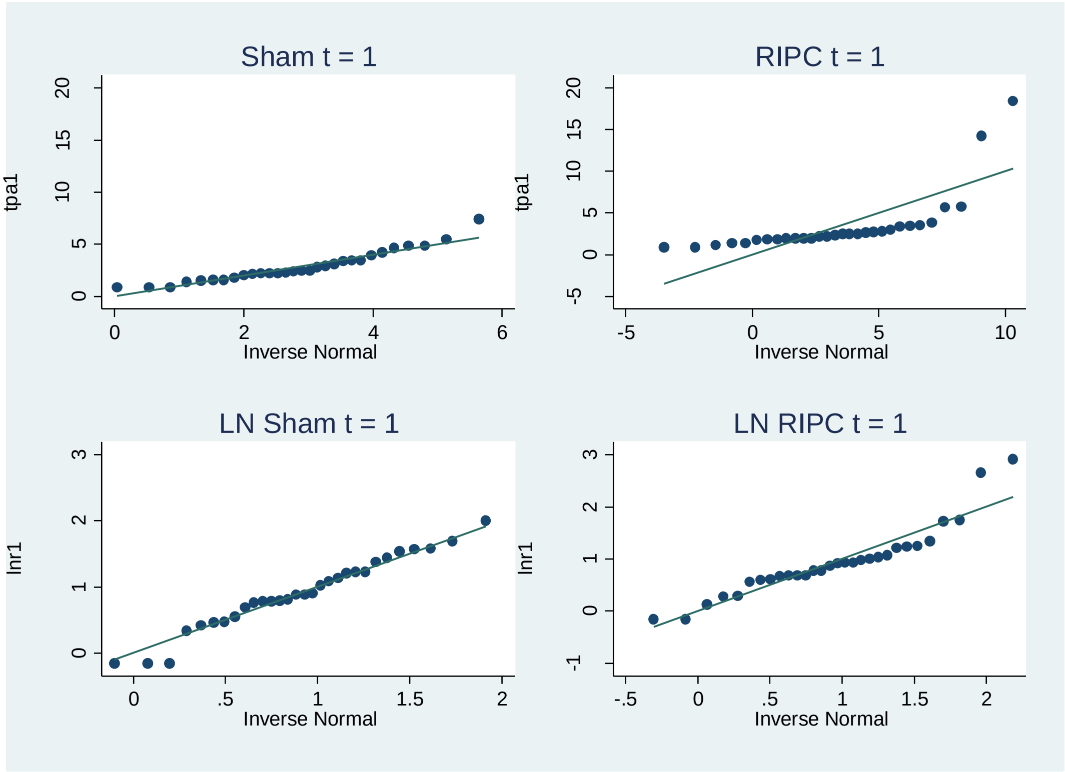

Sham t = 1
RIPC t = 1
LN Sham t = 1
LN RIPC t = 1
tpa1
lnr1
Inverse Normal

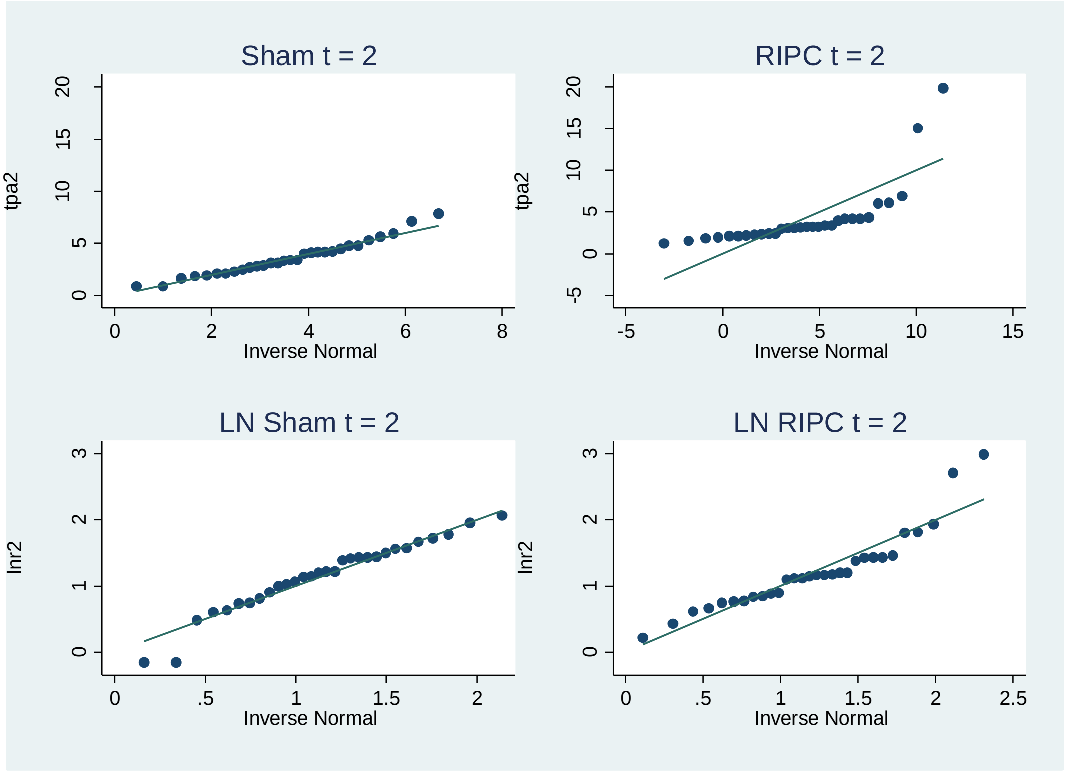

Sham t = 2
RIPC t = 2
LN Sham t = 2
LN RIPC t = 2
tpa2
lnr2
Inverse Normal

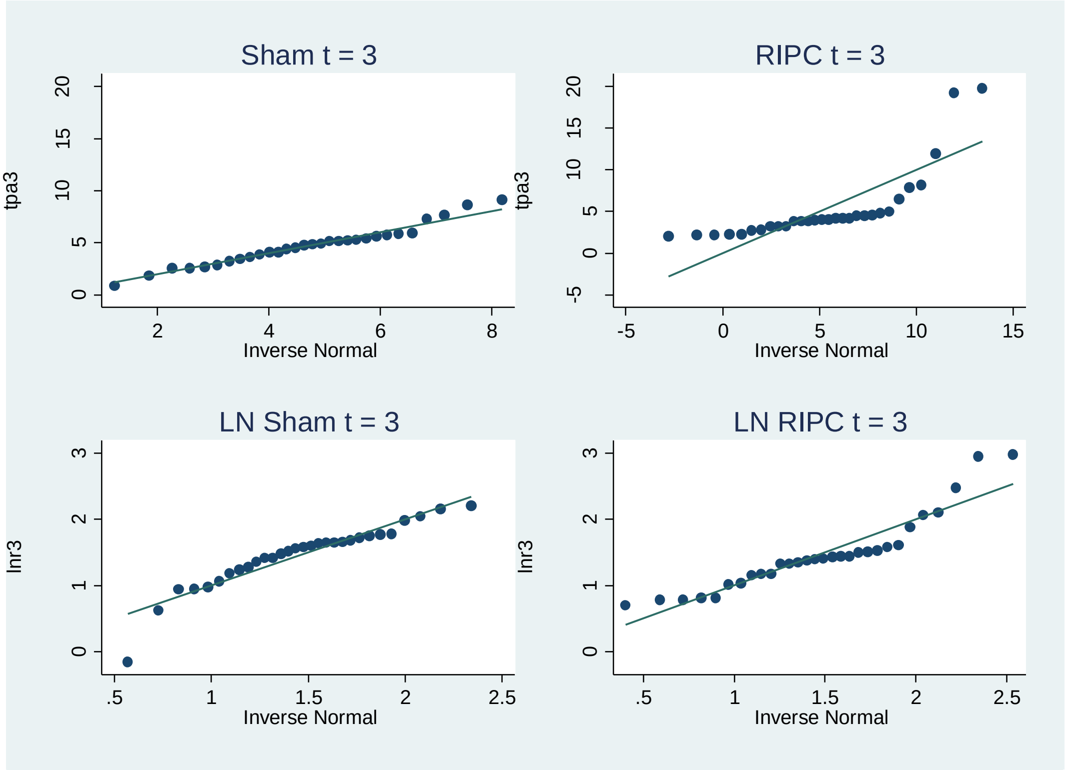
Sham t = 3
tpa3
Inverse Normal
RIPC t = 3
tpa3
Inverse Normal
LN Sham t = 3
lnr3
Inverse Normal
LN RIPC t = 3
lnr3
Inverse Normal

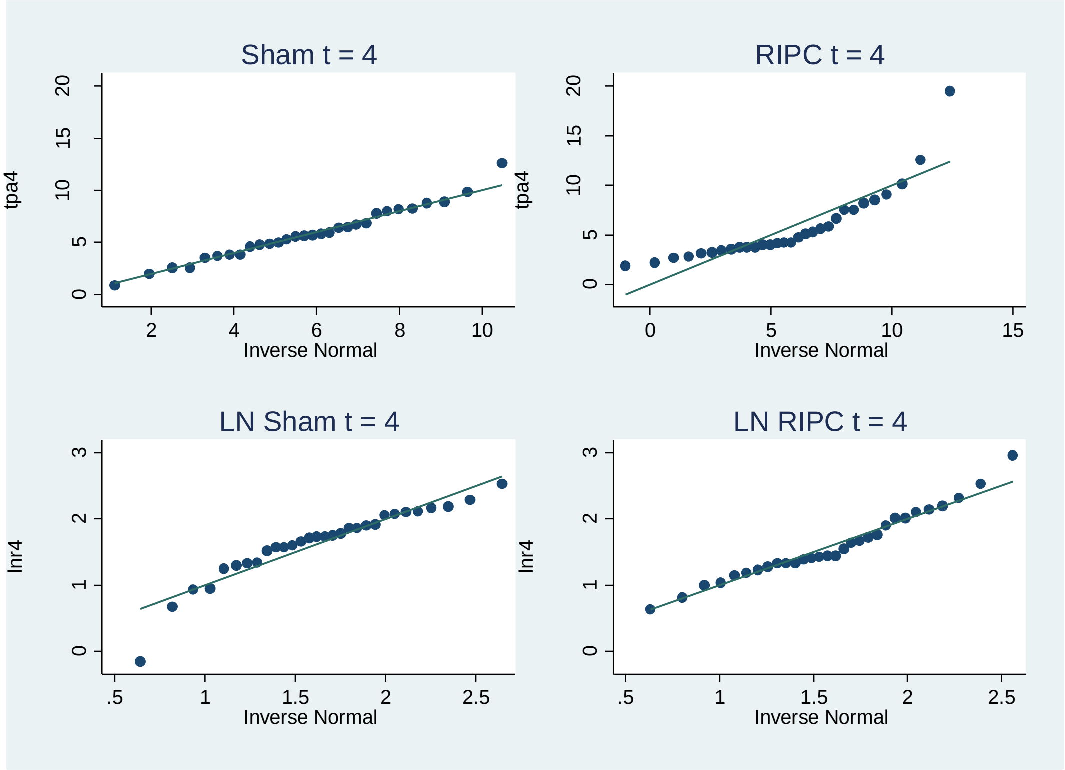
Sham t = 4
tpa4
Inverse Normal
RIPC t = 4
tpa4
Inverse Normal
LN Sham t = 4
lnr4
Inverse Normal
LN RIPC t = 4
lnr4
Inverse Normal

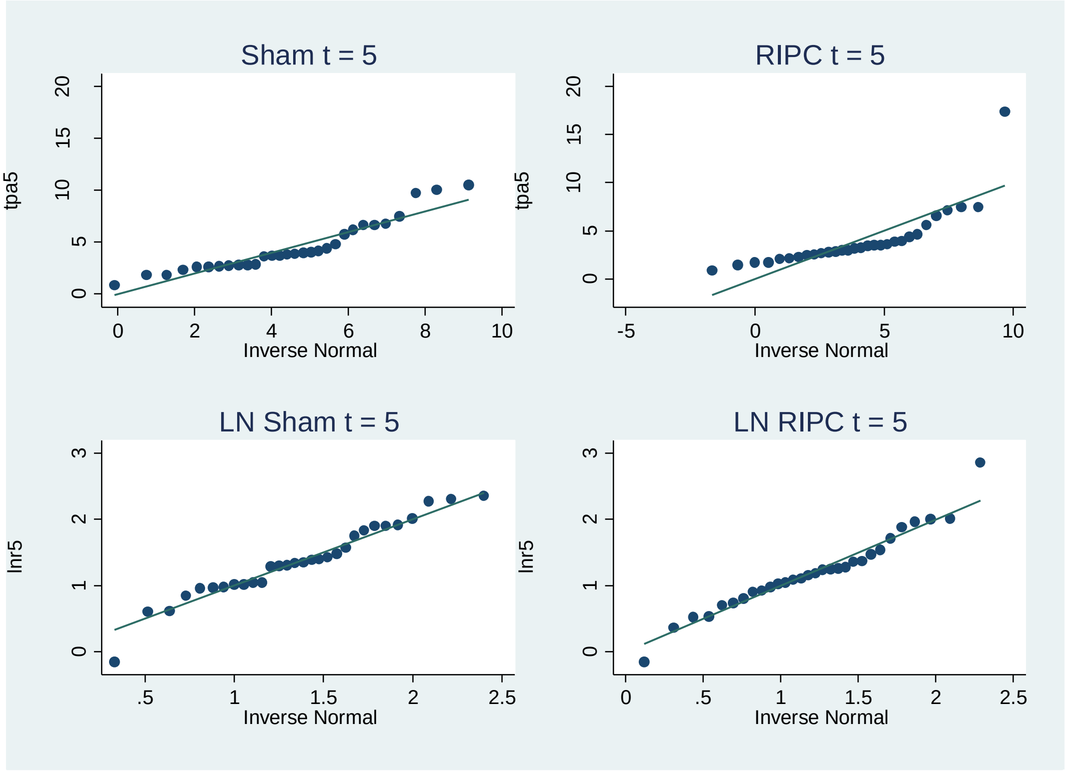

Sham t = 5
RIPC t = 5
LN Sham t = 5
LN RIPC t = 5
tpa5
lnr5
Inverse Normal

PAI-1

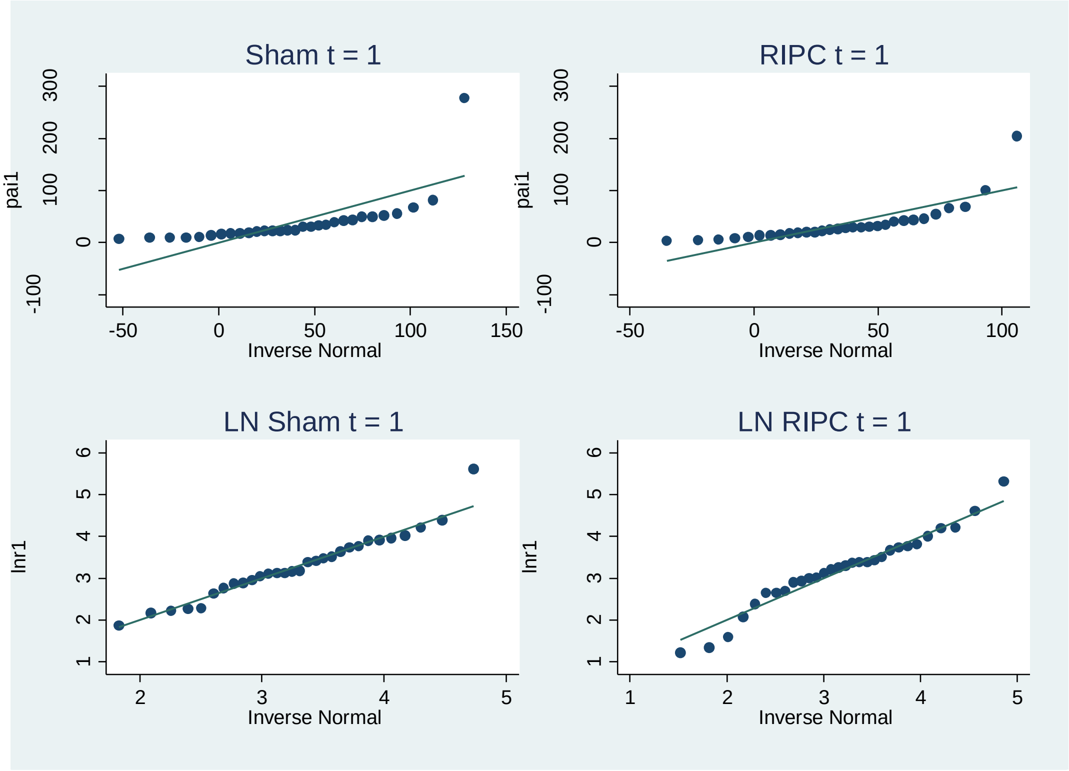

Sham t = 1
RIPC t = 1
LN Sham t = 1
LN RIPC t = 1
pai1
lnr1
Inverse Normal

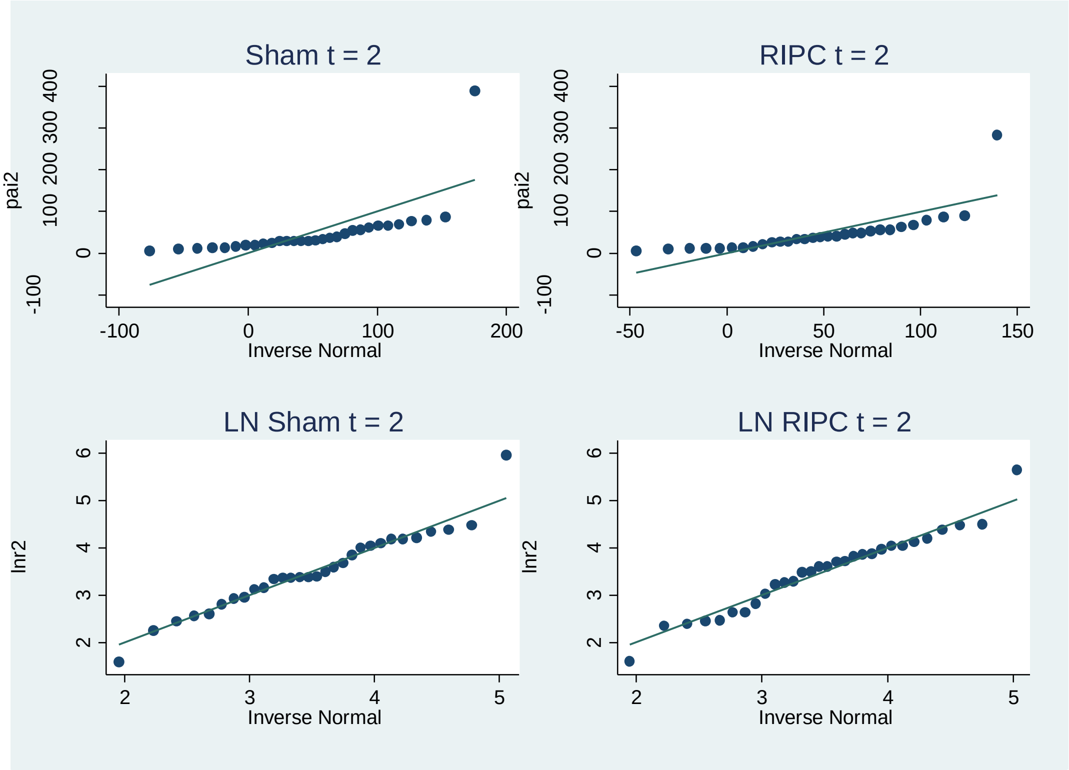
Sham t = 2
RIPC t = 2
pai2
-100
0
100 200 300 400
-100
0
100
200
Inverse Normal
-50
0
50
100
150
Inverse Normal
LN Sham t = 2
LN RIPC t = 2
lnr2
2
3
4
5
6
2
3
4
5
Inverse Normal

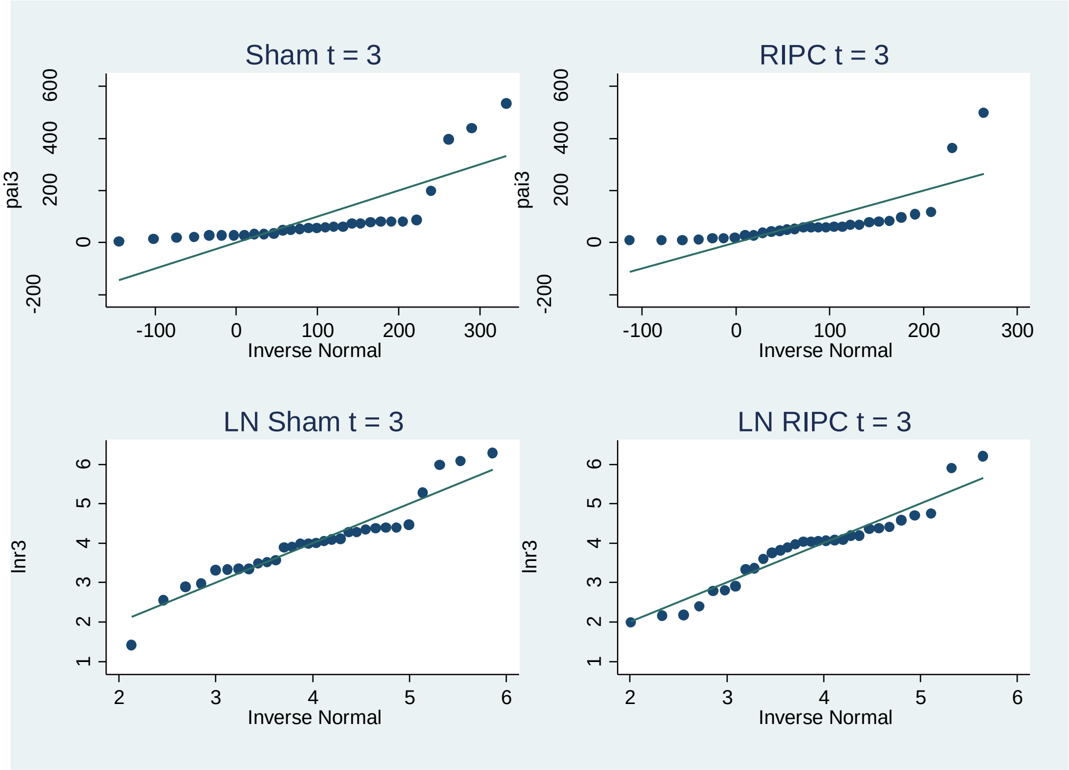
Sham t = 3
RIPC t = 3
pai3
-200
0
200
400
600
-100
0
100
200
300
Inverse Normal
LN Sham t = 3
LN RIPC t = 3
lnr3
1
2
3
4
5
6
2
3
4
5
6
Inverse Normal

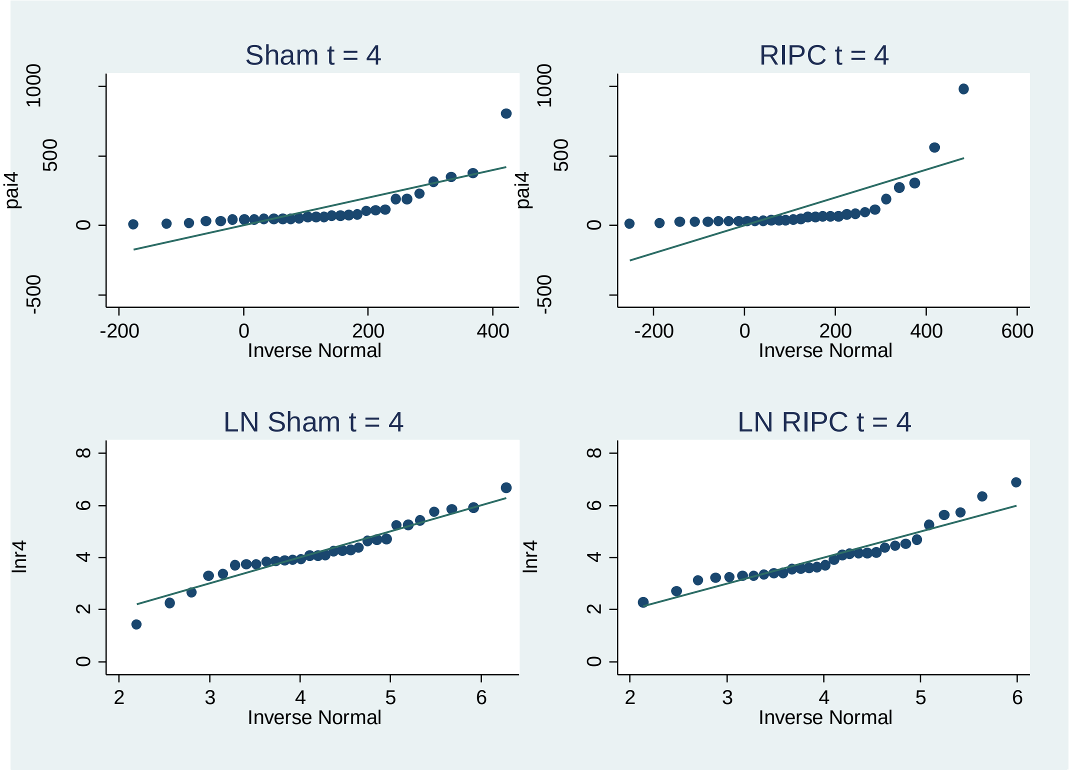
Sham t = 4
RIPC t = 4
LN Sham t = 4
LN RIPC t = 4
pai4
lnr4
Inverse Normal
-500 0 500 1000
-200 0 200 400
-200 0 200 400 600
0 2 4 6 8
2 3 4 5 6

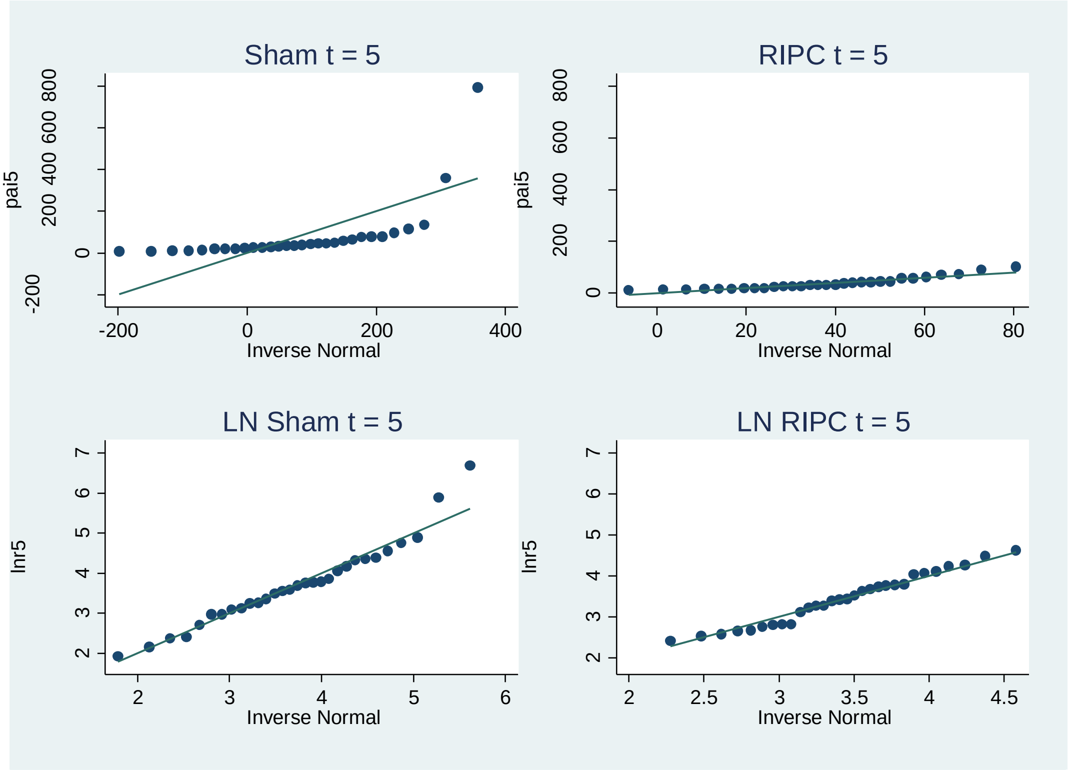
Sham t = 5
RIPC t = 5
LN Sham t = 5
LN RIPC t = 5
pai5
lnr5
Inverse Normal
-200 0 200 400 600 800
0 200 400 600 800
-200 0 200 400
0 20 40 60 80
2 3 4 5 6 7
2 2.5 3 3.5 4 4.5

Fibrinogen

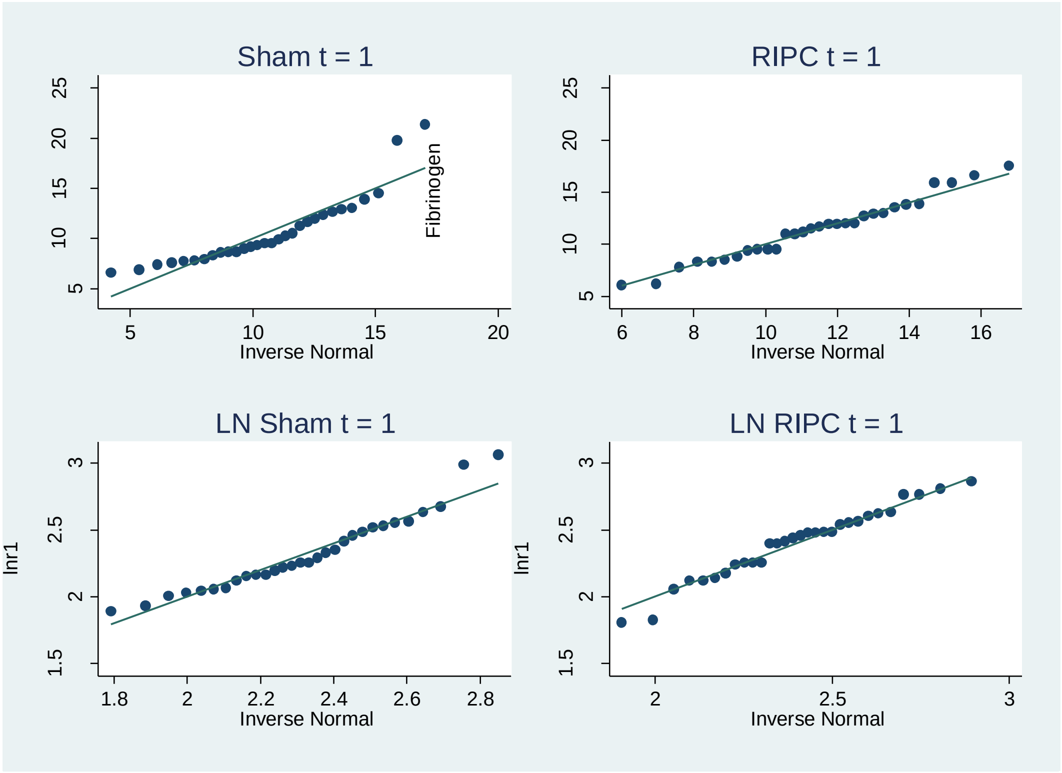
Sham t = 1
RIPC t = 1
LN Sham t = 1
LN RIPC t = 1
Fibrinogen
lnr1
Inverse Normal

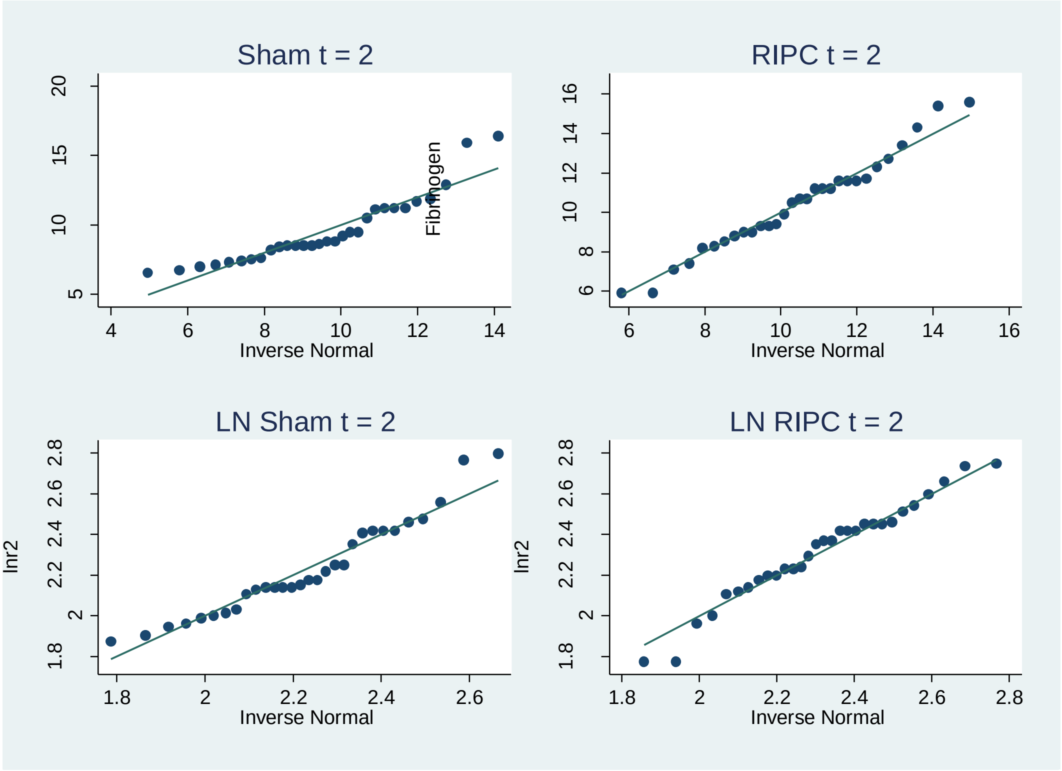
Sham t = 2
RIPC t = 2
LN Sham t = 2
LN RIPC t = 2
Fibrinogen
lnr2
Inverse Normal

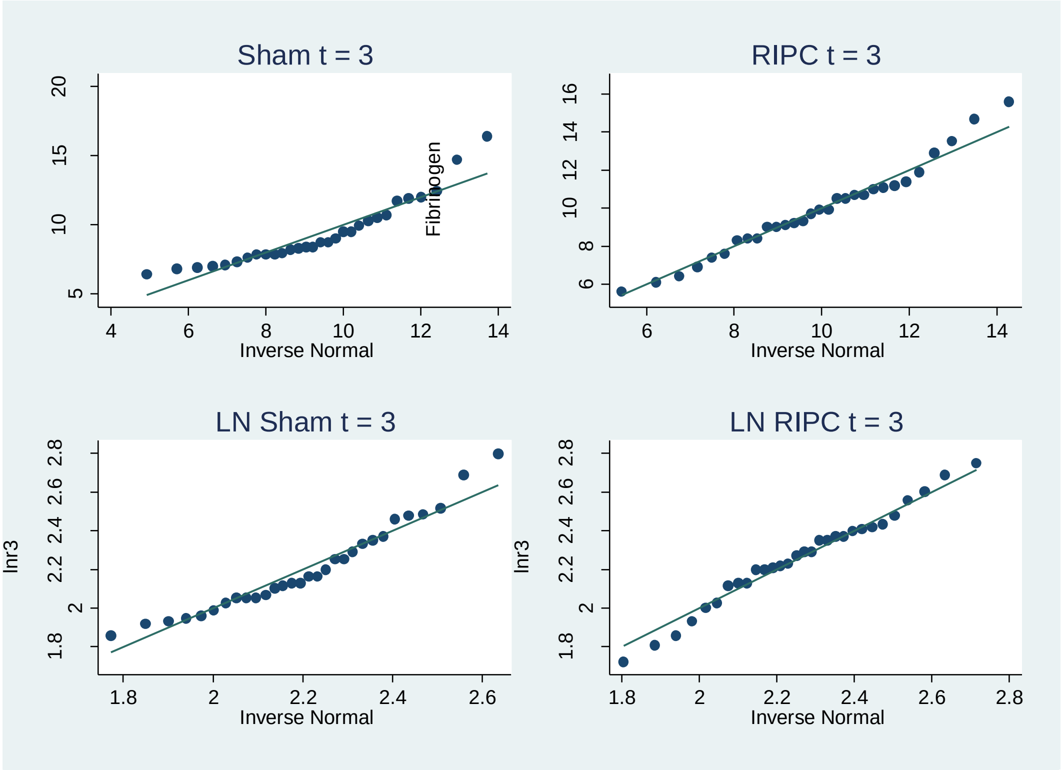
Sham t = 3
RIPC t = 3
LN Sham t = 3
LN RIPC t = 3
Fibrinogen
lnr3
Inverse Normal

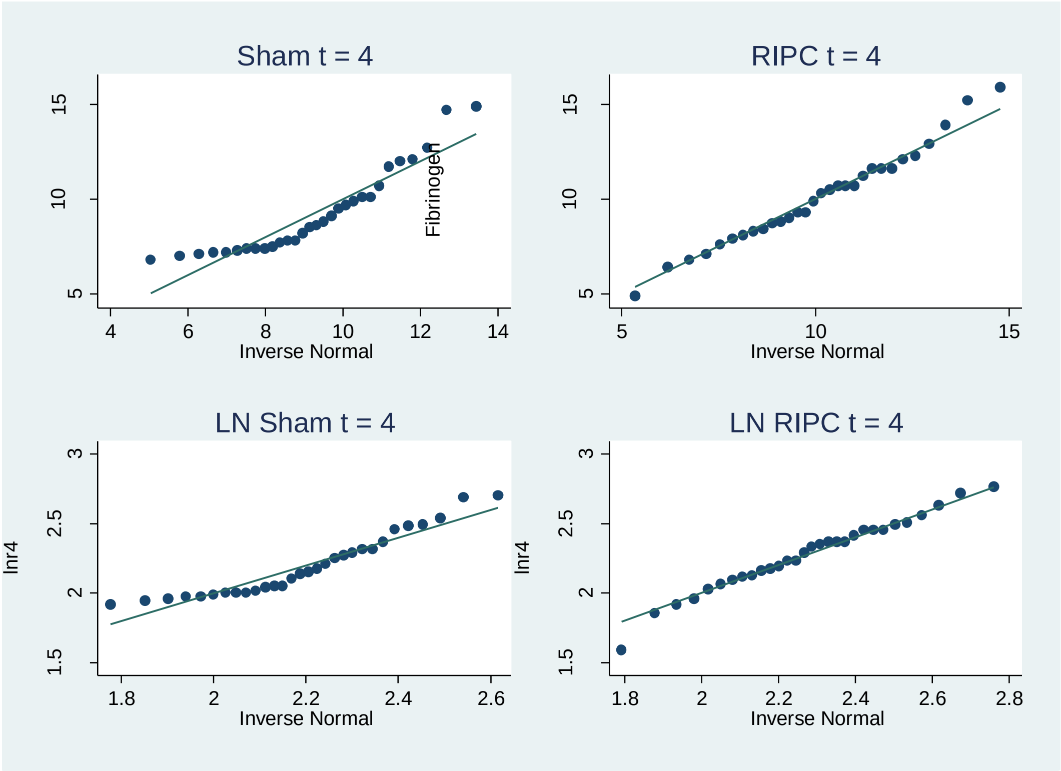
Sham t = 4
RIPC t = 4
LN Sham t = 4
LN RIPC t = 4
Fibrinogen
lnr4
Inverse Normal

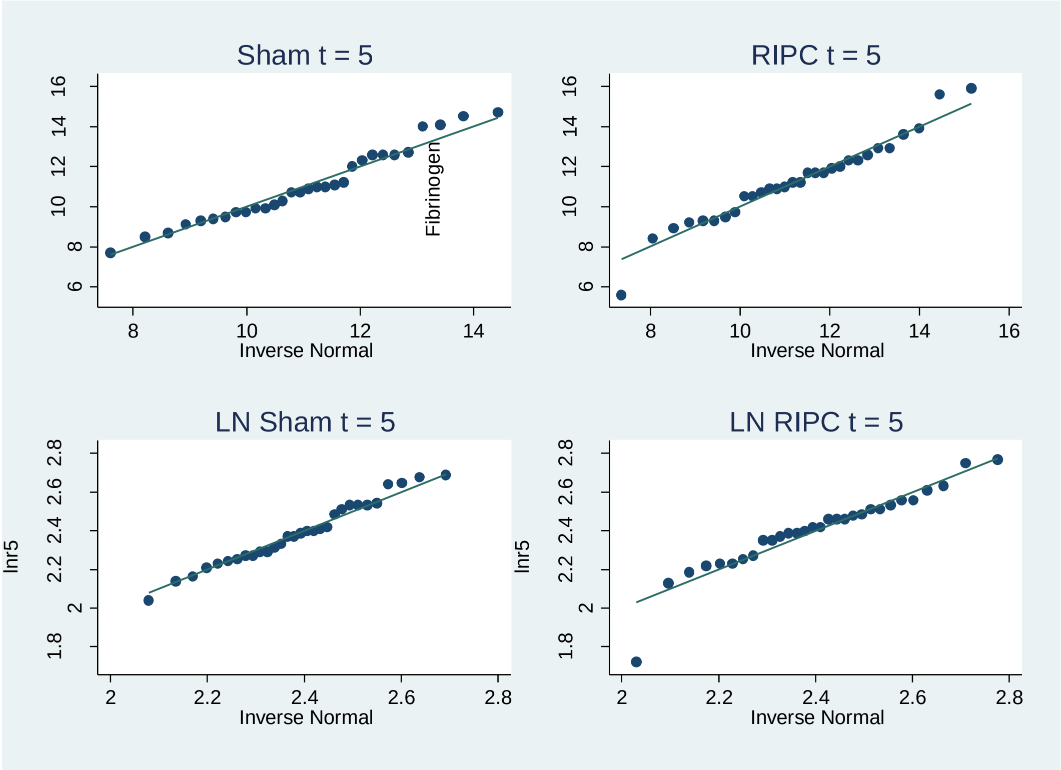
Sham t = 5
RIPC t = 5
Fibrinogen
Inverse Normal
Inverse Normal
LN Sham t = 5
LN RIPC t = 5
lnr5
lnr5
Inverse Normal
Inverse Normal

TAT

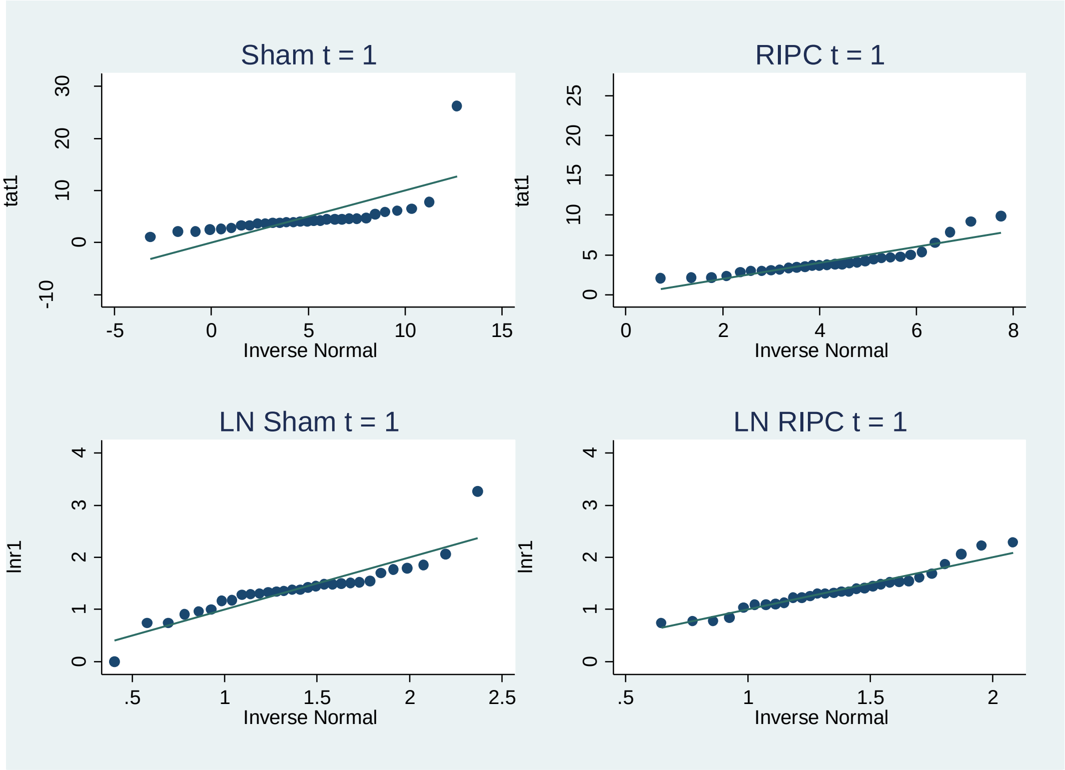
Sham t = 1
RIPC t = 1
tat1
tat1
Inverse Normal
Inverse Normal
LN Sham t = 1
LN RIPC t = 1
lnr1
lnr1
Inverse Normal
Inverse Normal

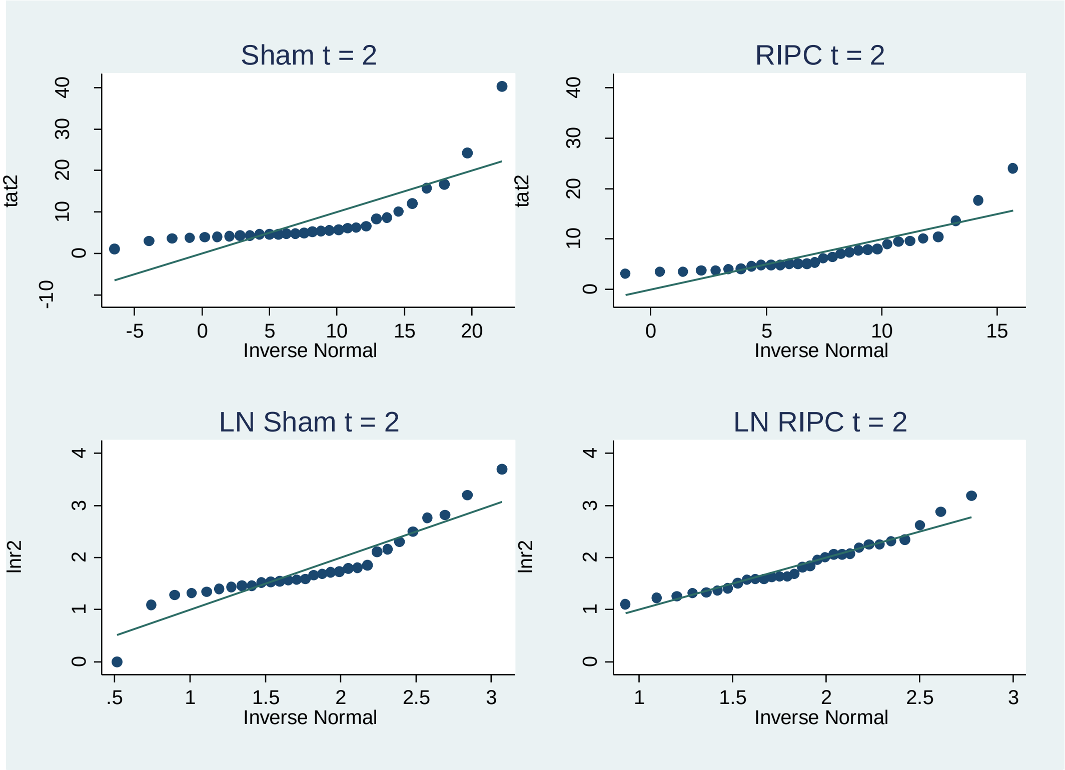
Sham t = 2
tat2
-10
0
10
20
30
40
-5
0
5
10
15
20
Inverse Normal
RIPC t = 2
tat2
0
10
20
30
40
0
5
10
15
Inverse Normal
LN Sham t = 2
lnr2
0
1
2
3
4
.5
1
1.5
2
2.5
3
Inverse Normal
LN RIPC t = 2
lnr2
0
1
2
3
4
1
1.5
2
2.5
3
Inverse Normal

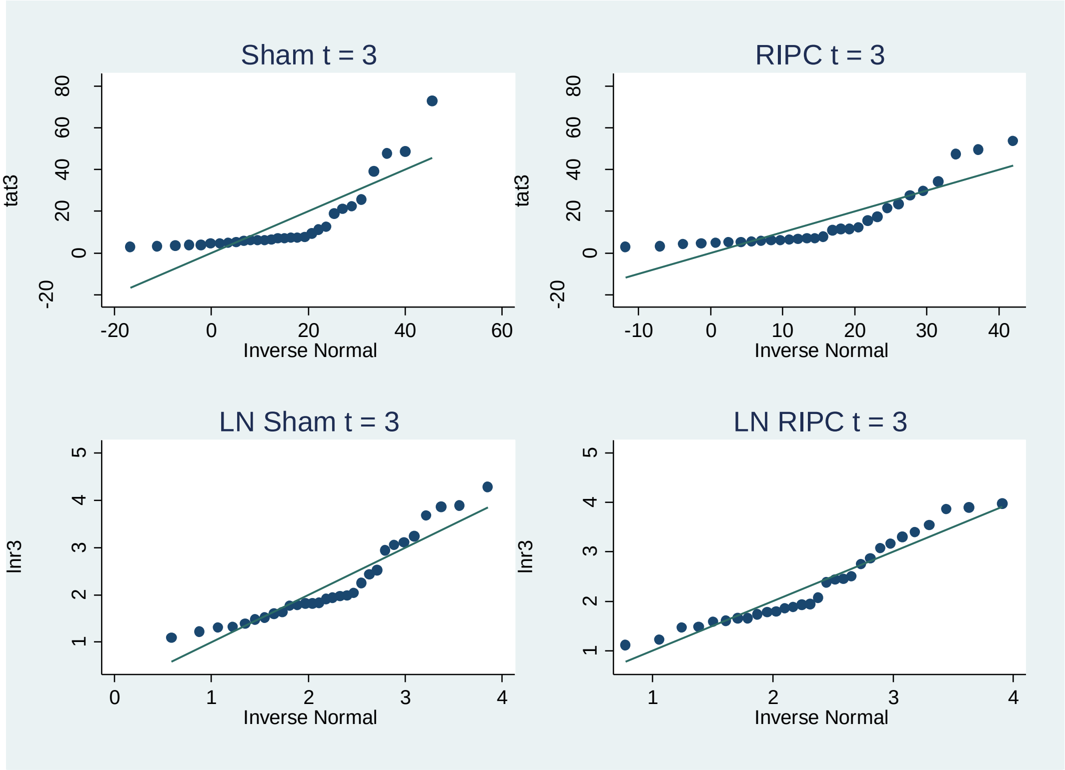
Sham t = 3
tat3
-20
0
20
40
60
80
-20
0
20
40
60
Inverse Normal
RIPC t = 3
tat3
-20
0
20
40
60
80
-10
0
10
20
30
40
Inverse Normal
LN Sham t = 3
lnr3
1
2
3
4
5
0
1
2
3
4
Inverse Normal
LN RIPC t = 3
lnr3
1
2
3
4
5
1
2
3
4
Inverse Normal

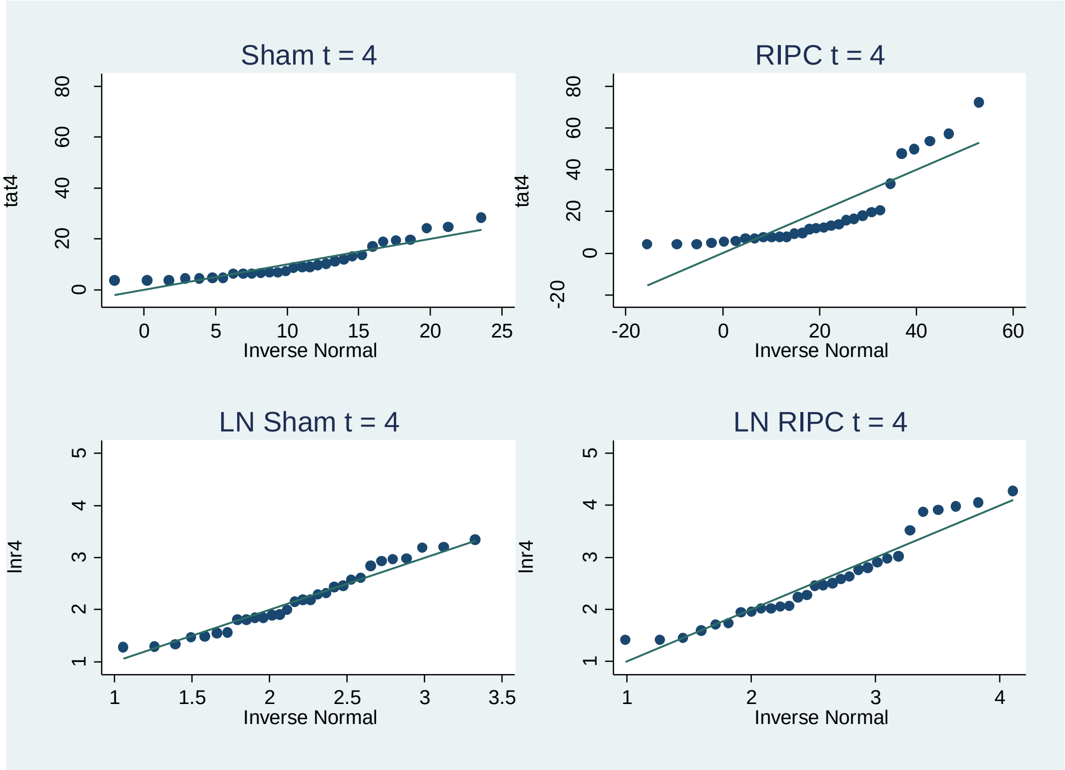
Sham t = 4
RIPC t = 4
LN Sham t = 4
LN RIPC t = 4
tat4
lnr4
Inverse Normal

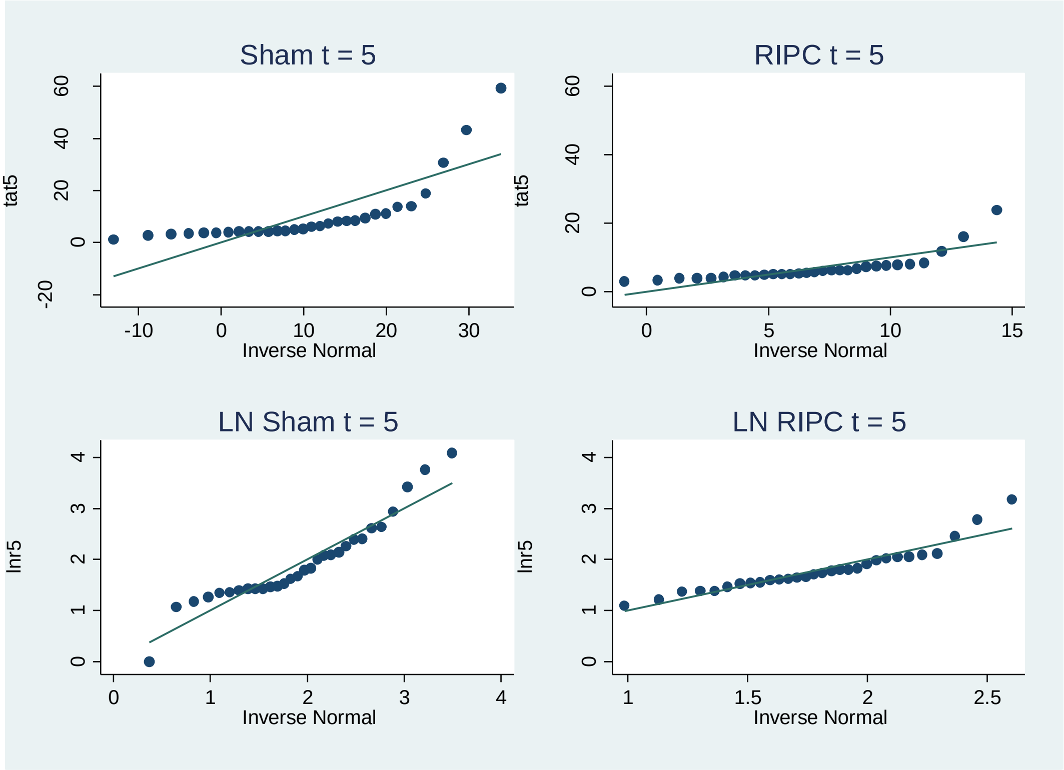
Sham t = 5
RIPC t = 5
LN Sham t = 5
LN RIPC t = 5
tat5
lnr5
Inverse Normal

F1+2

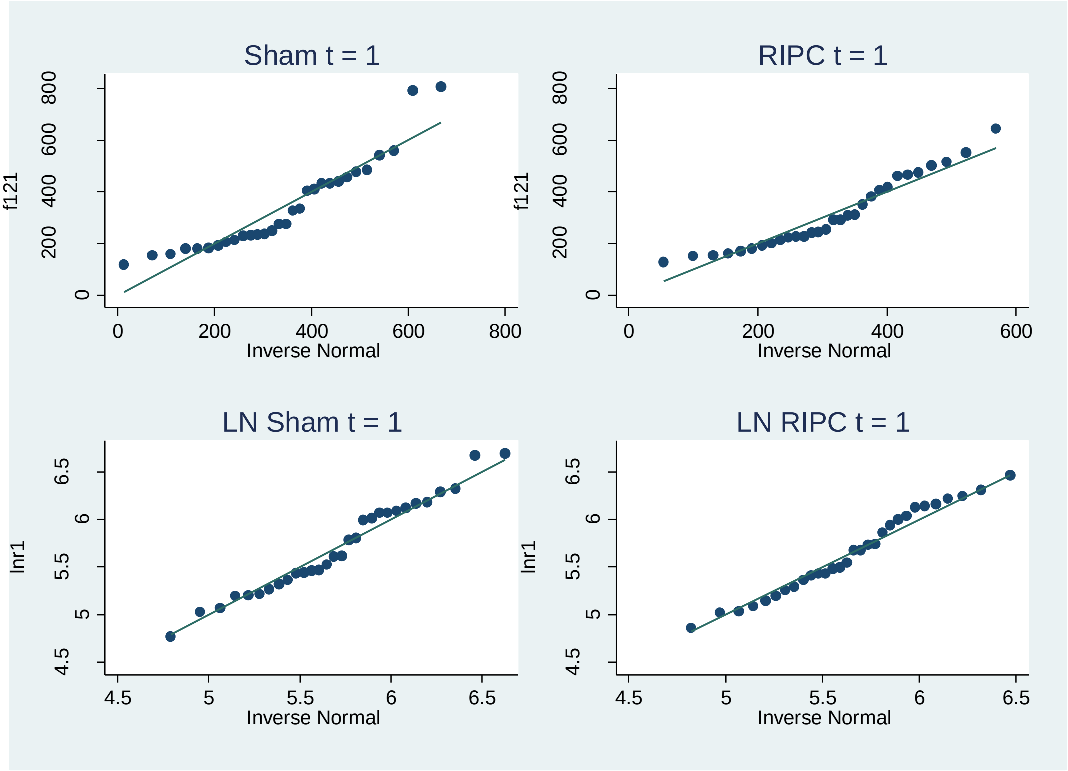
Sham t = 1
f121
Inverse Normal
RIPC t = 1
f121
Inverse Normal
LN Sham t = 1
lnr1
Inverse Normal
LN RIPC t = 1
lnr1
Inverse Normal

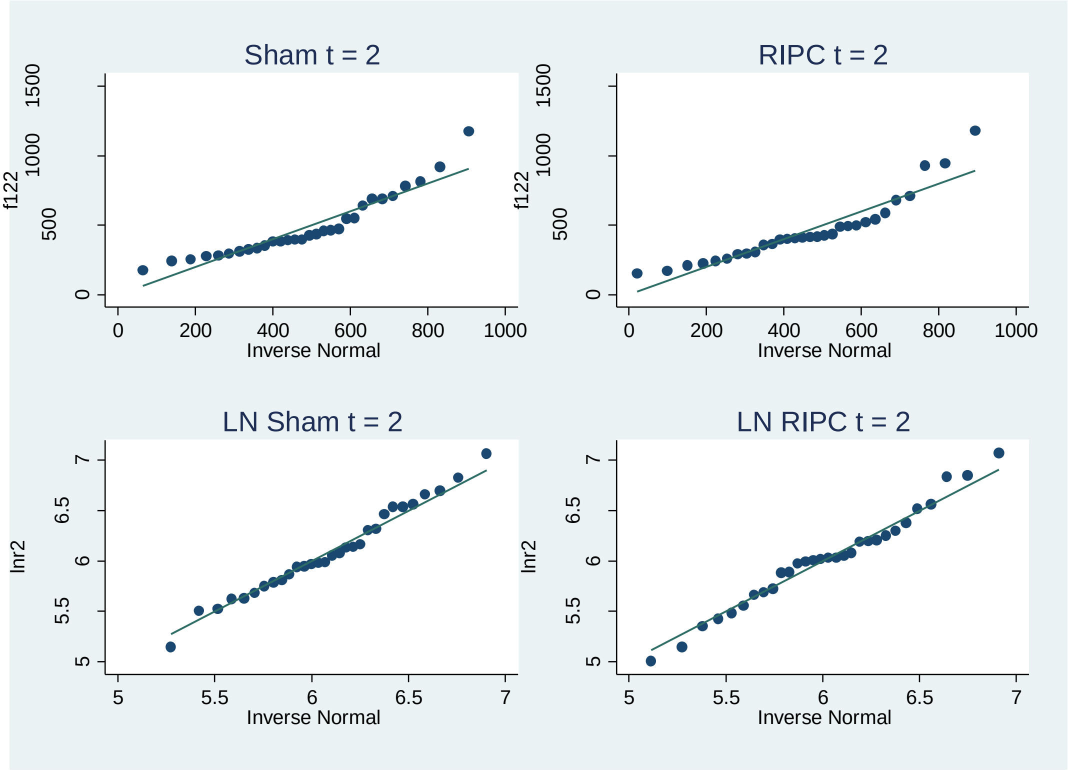
Sham t = 2
f122
Inverse Normal
RIPC t = 2
f122
Inverse Normal
LN Sham t = 2
lnr2
Inverse Normal
LN RIPC t = 2
lnr2
Inverse Normal

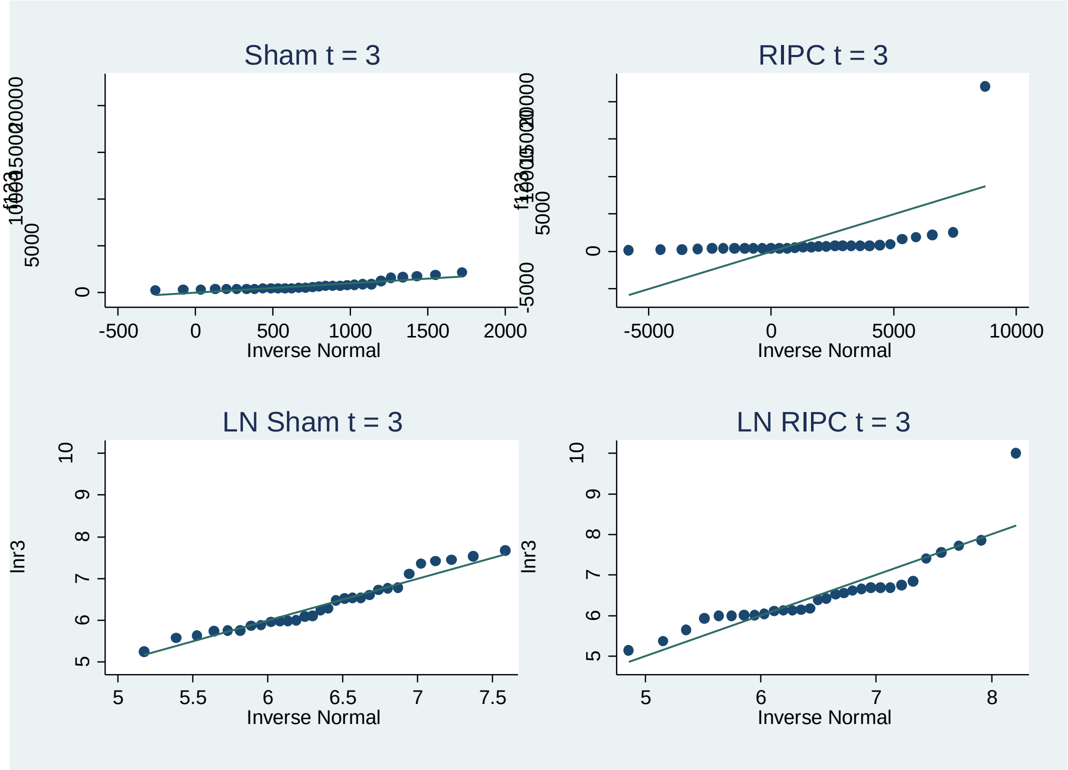
Sham t = 3
RIPC t = 3
f123
Inverse Normal
LN Sham t = 3
LN RIPC t = 3
lnr3
Inverse Normal

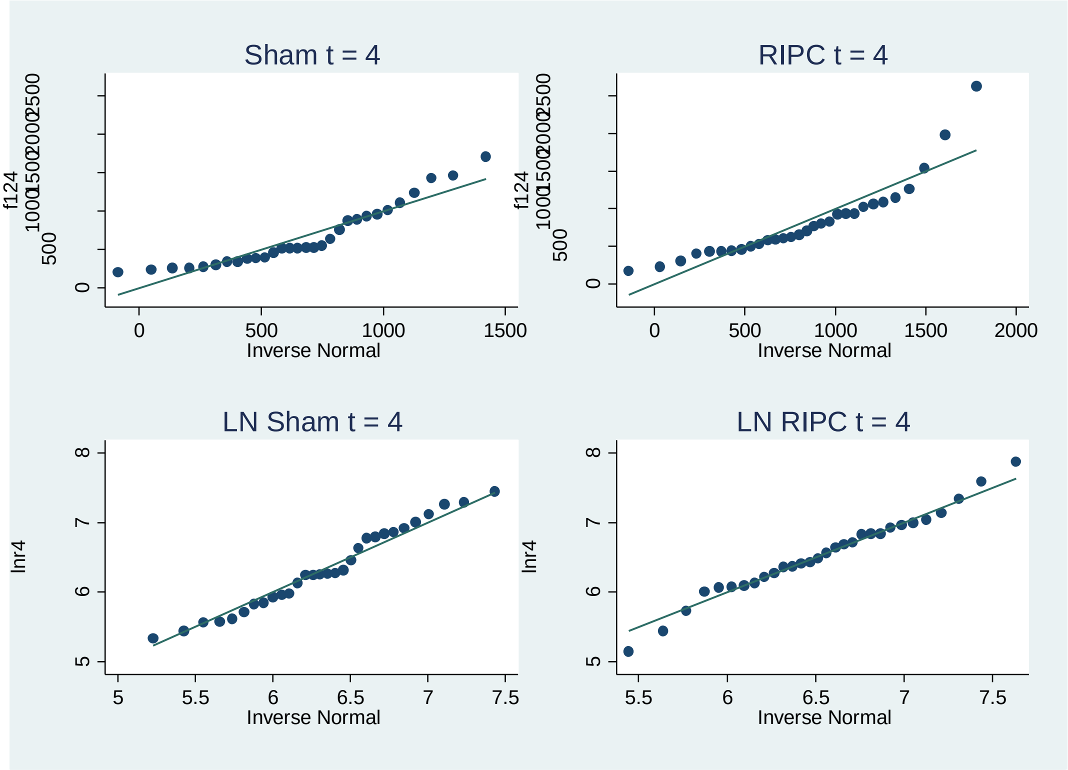
Sham t = 4
RIPC t = 4
f124
Inverse Normal
LN Sham t = 4
LN RIPC t = 4
lnr4
Inverse Normal

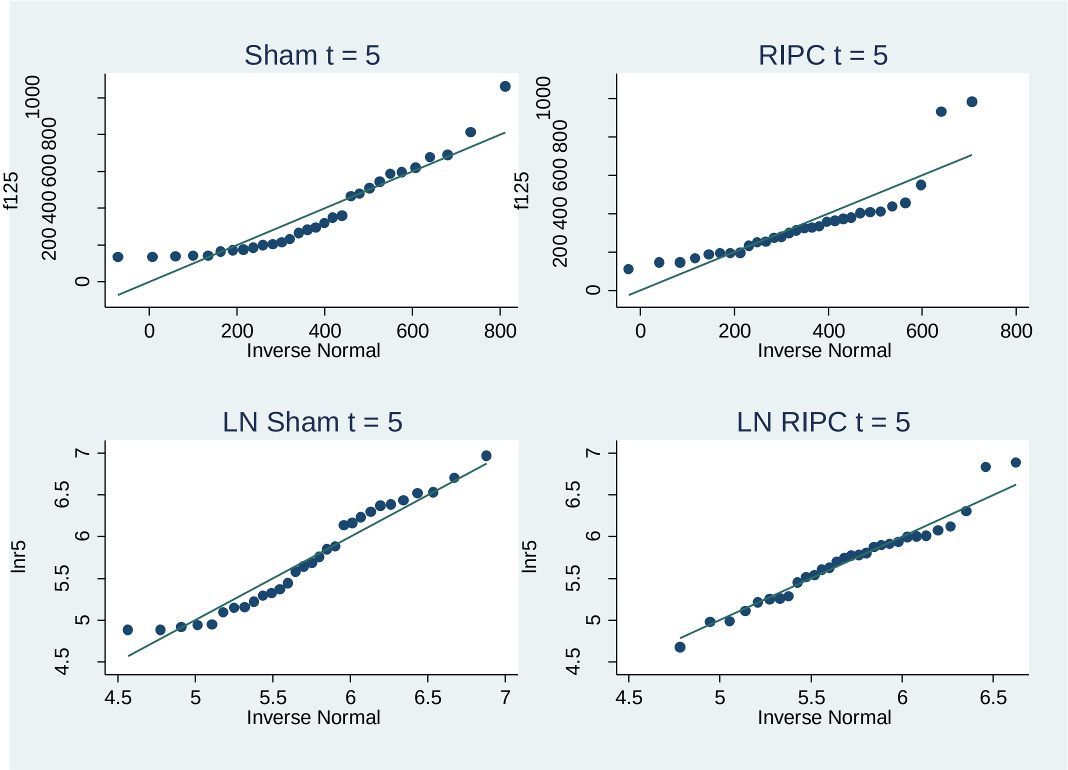
Sham t = 5
f125
0
200 400 600 800
1000
0
200
400
600
800
Inverse Normal
RIPC t = 5
f125
0
200 400 600 800
1000
0
200
400
600
800
Inverse Normal
LN Sham t = 5
lnr5
4.5
5
5.5
6
6.5
7
4.5
5
5.5
6
6.5
7
Inverse Normal
LN RIPC t = 5
lnr5
4.5
5
5.5
6
6.5
7
4.5
5
5.5
6
6.5
Inverse Normal

aPTT

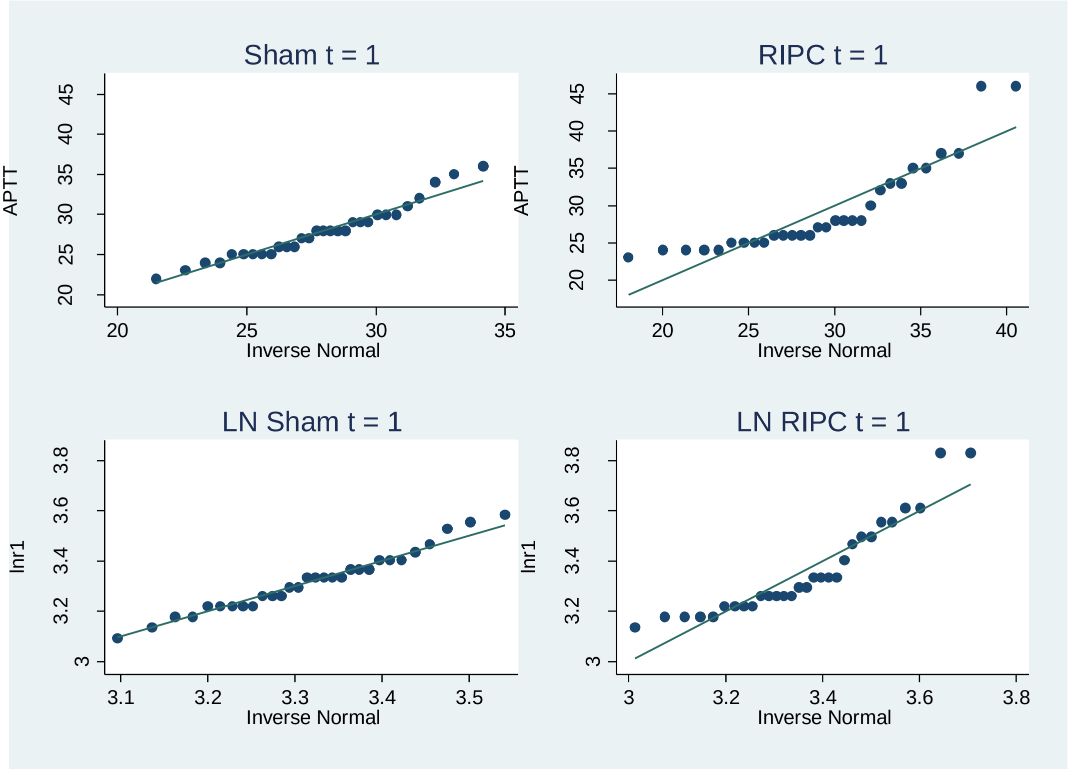
Sham t = 1
APTT
20
25
30
35
40
45
20
25
30
35
Inverse Normal
RIPC t = 1
APTT
20
25
30
35
40
45
20
25
30
35
40
Inverse Normal
LN Sham t = 1
lnr1
3
3.2
3.4
3.6
3.8
3.1
3.2
3.3
3.4
3.5
Inverse Normal
LN RIPC t = 1
lnr1
3
3.2
3.4
3.6
3.8
3
3.2
3.4
3.6
3.8
Inverse Normal

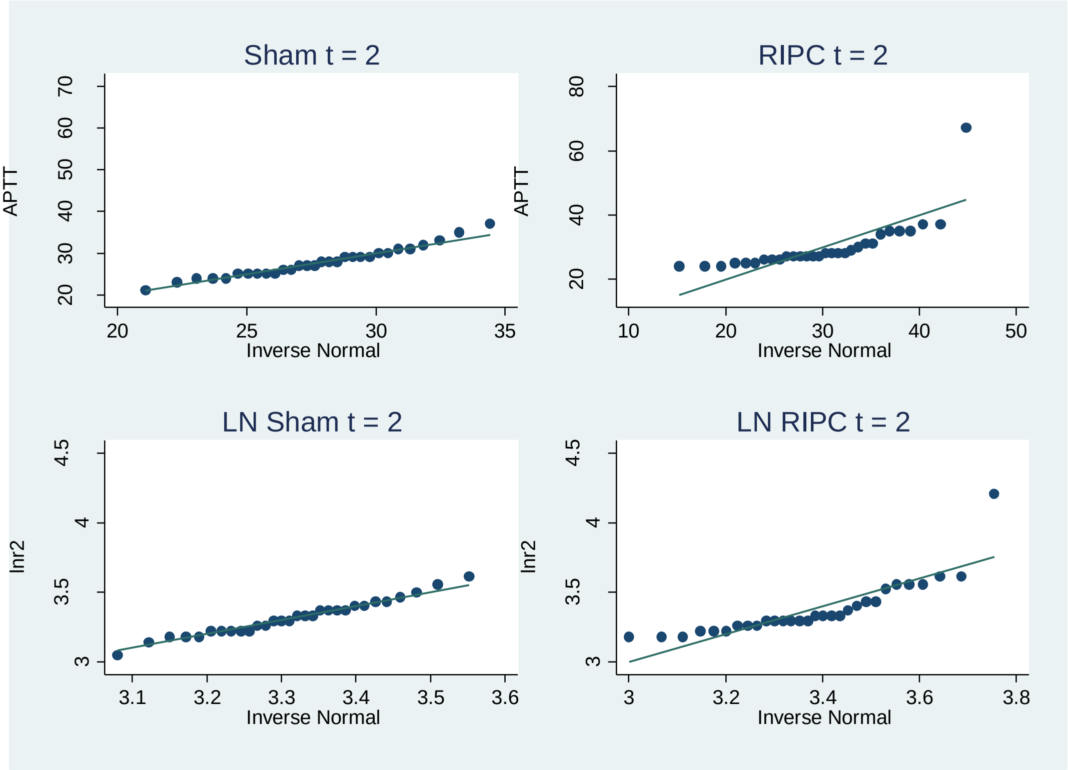
Sham t = 2
RIPC t = 2
LN Sham t = 2
LN RIPC t = 2
APTT
lnr2
Inverse Normal

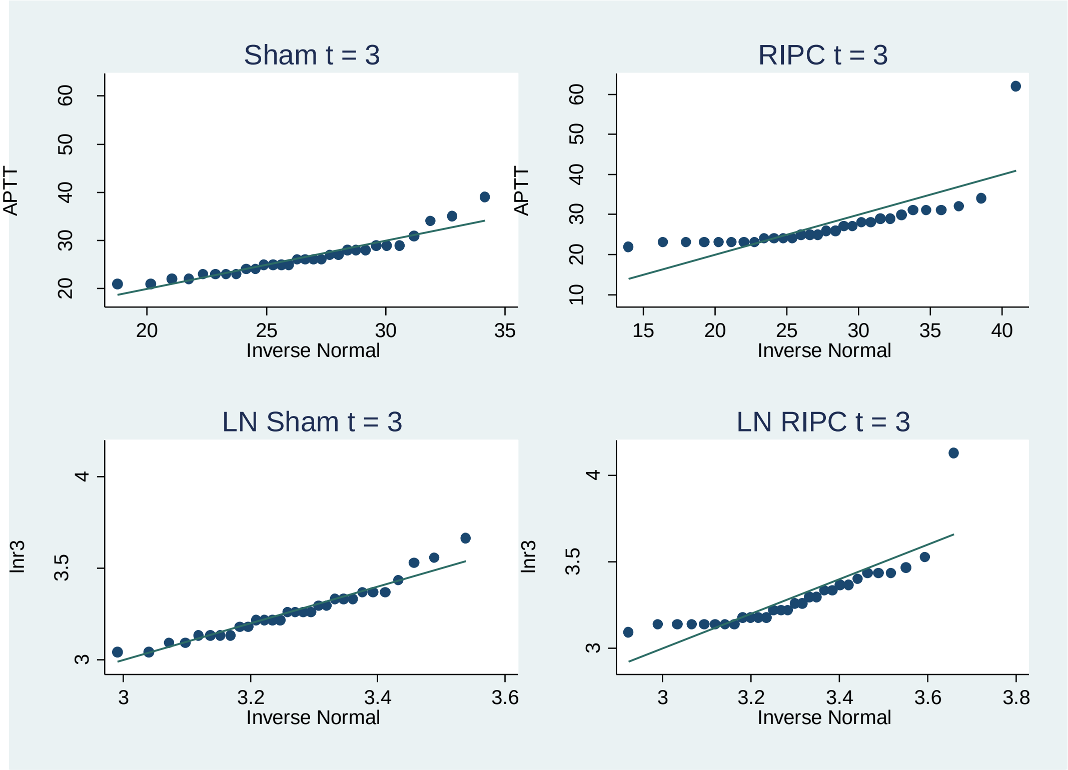
Sham t = 3
RIPC t = 3
LN Sham t = 3
LN RIPC t = 3
APTT
lnr3
Inverse Normal

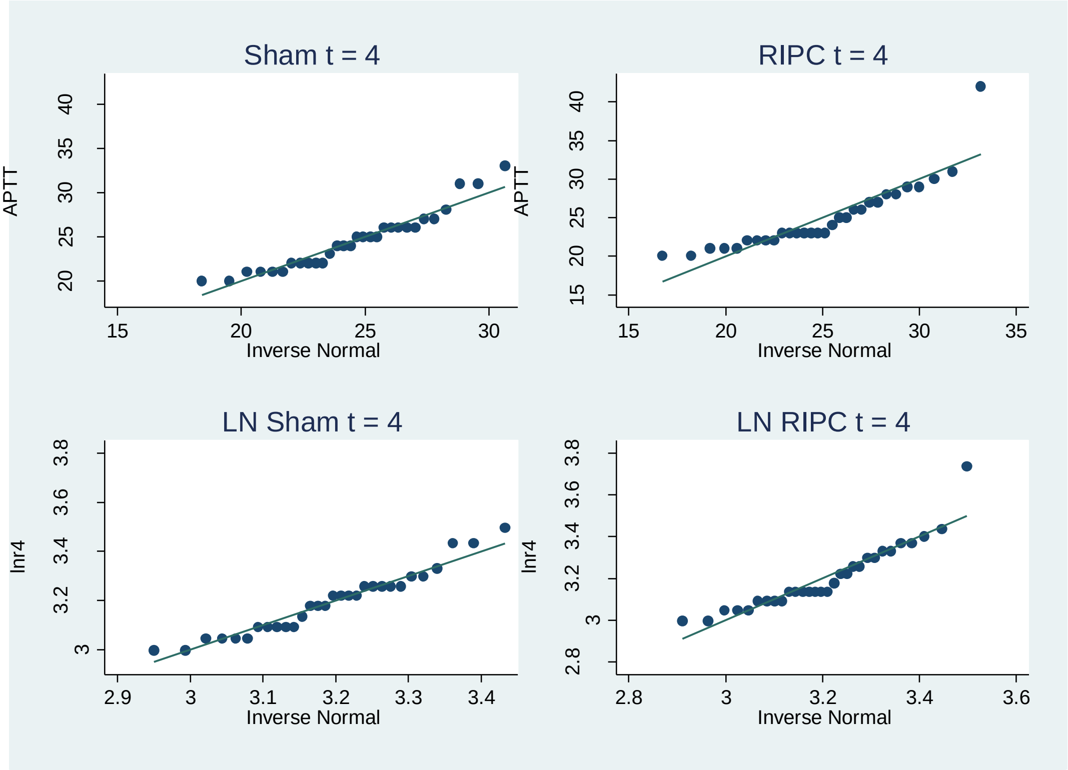
Sham t = 4
RIPC t = 4
LN Sham t = 4
LN RIPC t = 4
APTT
lnr4
Inverse Normal

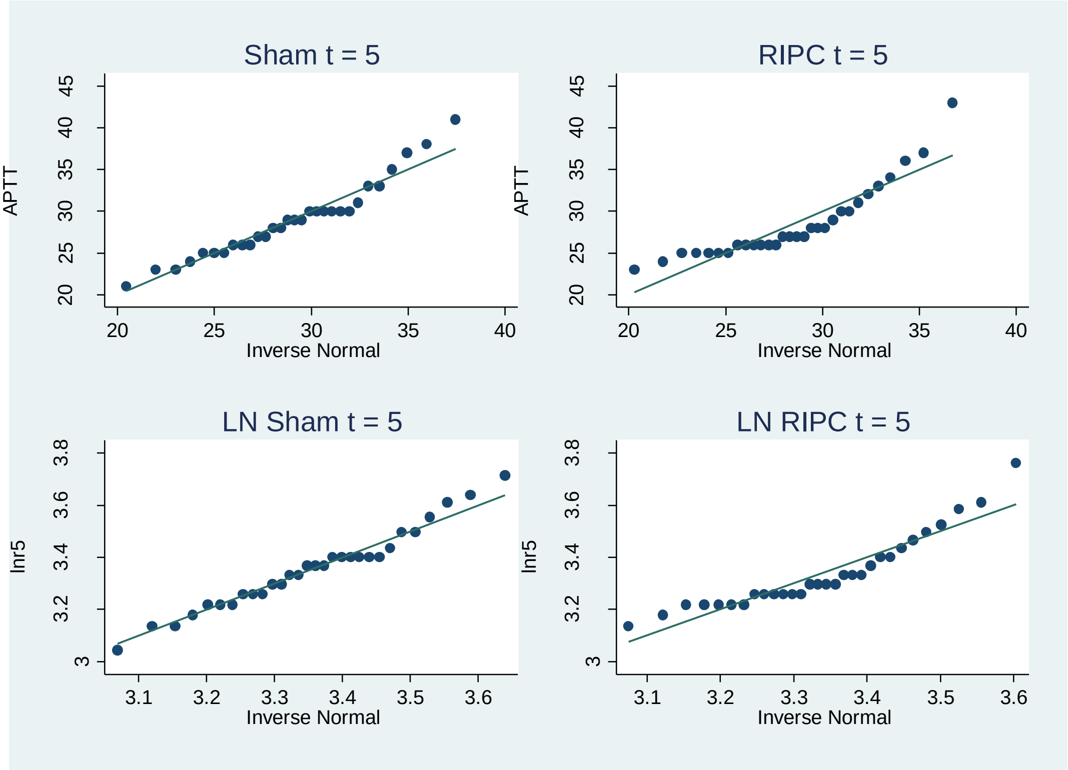
Sham t = 5
RIPC t = 5
LN Sham t = 5
LN RIPC t = 5
APTT
lnr5
Inverse Normal
